# Shuxuening injection for treating acute ischemic stroke: a PRISMA-compliant systematic review and meta-analysis of randomized controlled trials

**DOI:** 10.3389/fphar.2024.1407669

**Published:** 2024-10-22

**Authors:** Jie Zhan, Xiaowen Xu, Yanzhen Zhu, Lin Liu, Hongxia Chen, Lechang Zhan

**Affiliations:** ^1^ Department of Rehabilitation, Guangdong Provincial Hospital of Chinese Medicine, The Second Affiliated Hospital of Guangzhou University of Chinese Medicine, Guangzhou, China; ^2^ The Second Clinical College of Guangzhou University of Chinese Medicine, Guangzhou, China; ^3^ Medical College of Acu-Moxi and Rehabilitation, Guangzhou University of Chinese Medicine, Guangzhou, China

**Keywords:** shuxuening injection, acute ischemic stroke, traditional Chinese medicine, systematic review, meta-analysis

## Abstract

**Background:** Using Shuxuening injection (SXNI) for acute ischemic stroke (AIS) is popular in China, but its efficacy and safety remain controversial.

**Purpose:** This study aims to assess the efficacy and safety of SXNI as an add-on therapy for AIS.

**Study design:** Systematic review and meta-analysis.

**Methods:** We searched for randomized controlled trials (RCTs) on SXNI for AIS in seven databases and two clinical trial registration platforms from their inception to January 2023. We used the Cochrane risk of bias tool to assess the methodological quality of the included studies and performed the meta-analysis with R software. The primary outcome was clinical efficacy, assessed by the clinical effective rate (CER). The secondary outcomes were neurological function, activities of daily living (ADL), and adverse events (AEs).

**Results:** In total, 116 studies with 12,401 participants were included in this review. Fifteen (12.9%) studies were judged to be of moderate to high quality. SXNI plus conventional treatments (CTs) improved the CER compared with CTs alone (risk ratio [RR]: 1.21, 95% confidence interval [CI]: 1.17–1.25, *p* < 0.0001) or CTs plus other injections (RR: 1.18, 95% CI: 1.15–1.21, *p* < 0.0001). SXNI plus CTs reduced the National Institute of Health Stroke Scale score compared with CTs alone (mean difference [MD]: −4.00, 95% CI: −5.22 to −2.78, *p* < 0.0001) or CTs plus other injections (MD: −2.28, 95% CI: −3.41 to −1.16, *p* < 0.0001). SXNI plus CTs also decreased the Chinese Stroke Scale score compared with CTs alone (MD:

−5.01, 95% CI: −7.38 to −2.65, *p* < 0.0001) or CTs plus other injections (MD: −4.31, 95% CI: −5.75 to −2.88, *p* < 0.0001). SXNI plus CTs was superior for increasing the Barthel index score compared with CTs alone (MD: 11.58, 95% CI: 8.27–14.90, *p* < 0.0001) or CTs plus other injections (MD: 5.43, 95% CI: 0.48–10.39, *p* = 0.0317). The level of evidence for each outcome was assessed as low to very low. The most common AEs of SXNI were cardiovascular system events, and all these AEs were mild.

**Conclusion:** SXNI combined with CTs maybe better than CTs alone or CTs plus other injections for improving the clinical efficacy, neurological function, and ADL of AIS patients, with relatively reliable safety. However, due to the low quality of the included studies, more rigorously designed RCTs with large sample sizes should be conducted in the future.

**Systematic Review Registration:**

www.crd.york.ac.uk, identifier (CRD42023418565).

## 1 Introduction

Stroke is the third leading cause of death and disability globally, especially in low- and middle-income countries (GBD, 2019 Stroke Collaborators, 2021). From 1990 to 2019, the absolute number of global stroke events increased by 70.0%, and stroke mortality increased by 43.0% worldwide (GBD, 2019 Stroke Collaborators, 2021). Stroke patients often have motor dysfunction, loss of activities of daily living (ADL) (Jørgensen et al., 1999), and a significant decline in quality of life, which prevents them from returning to work, thereby resulting in economic losses for the country’s productivity (Rochmah et al., 2021). According to the American Heart Association, the total cost of stroke, including direct and indirect expenditures, is projected to increase from $105.2 billion in $2012 to $240.7 billion in 2030 (Ovbiagele et al., 2013).

Acute ischemic stroke (AIS) accounts for most of all strokes. The appropriate treatment for AIS is significantly associated with prognosis (Hasan et al., 2021; Rosa et al., 2022). The primary therapeutic goal of AIS is to restore perfusion of ischemic brain tissue, and the main treatments include intravenous thrombolysis (IVT) and/or endovascular thrombectomy (EVT) (Hasan et al., 2021). However, 3%–8% of patients receiving IVT experience symptomatic cerebral hemorrhage (Gumbinger et al., 2012). Due to the narrow time window, contraindications, and the relatively low recanalization rate of large artery occlusions, the number of AIS patients who benefit from IVT is not as large as expected (Bhatia et al., 2010). EVT can significantly improve the prognosis of stroke; however, most interventional neuroradiologists work in urban areas, while half of the world’s population lives in rural areas, resulting in limited access to EVT (Wassélius et al., 2022). Therefore, it is necessary to find an effective and safe therapy with a longer time window to improve the prognosis of stroke and address its burden.

Shuxuening injection (SXNI) extracted from *Ginkgo biloba* is a commercial Chinese polyherbal preparation that is widely used for stroke in China (Cui et al., 2020; Li et al., 2023). SXNI can dilate blood vessels and improve blood circulation, and it is mainly used for ischemia cardio-cerebrovascular diseases, such as cerebral infarction, vasospasm and coronary heart disease. The pharmacologically active metabolites of SXNI are flavonol glycosides and ginkgolide (Smith et al., 1996; Shi et al., 2009). Numerous *in vitro* and *in vivo* studies have confirmed the neuroprotective effect of *G. biloba* extract (GBE) (Oyama et al., 1996; Guidetti et al., 2001; Ahlemeyer and Krieglstein, 2003). GBE can improve blood circulation, strengthen capillary walls, prevent thrombosis, and protect nerve cells from damage during hypoxia (Singh et al., 2019). Two studies (Oskouei et al., 2013; Li et al., 2017) showed that GBE was better than placebo or aspirin in improving stroke patients’ neurological function, as assessed by the National Institutes of Health Stroke Scale (NIHSS). More interestingly, GBE can work within 1–3 h (Diamond et al., 2000). Compared with IVT or EVT, SXNI not only has a longer treatment time window and cheaper costs with fewer side effects (Li et al., 2023) but can also be implemented in urban and rural medical places without specially higher professional requirements for doctors. However, due to the complexity of the metabolites of SXNI, it requires more careful monitoring for patients to prevent adverse events (AEs) while administering the drug.

Previous systematic reviews published in 2011, 2012, and 2016 confirmed the efficacy of SXNI for treating AIS in terms of the clinical efficacy assessed by the clinical effective rate (CER), with a very low level of evidence. However, some outcomes that are conducive to assessing the overall status of stroke patients, such as neurological function assessed by internationally recognized tools (e.g., the NIHSS), activities of daily living (ADL), and AEs, were not reported in these reviews. In addition, numerous RCTs on SXNI for AIS have been published since 2016, and it is necessary to update the evidence. We aimed to comprehensively assess the efficacy and safety of SXNI as an add-on therapy for AIS in terms of clinical efficacy, neurological function, ADL, and AEs.

## 2 Methods

This study was conducted strictly following the Preferred Reporting Items for Systematic Reviews and Meta-Analysis (PRISMA) guidelines (Page et al., 2021). The protocol was registered at PROSPERO (www.crd.york.ac.uk), and the registration number was CRD42023418565.

### 2.1 Types of studies

All RCTs about SXNI for AIS were eligible for inclusion. The language of publication was not limited. We excluded case-control studies, retrospective studies, case reports, animal experiments, cell experiments, reviews, meta-analyses, clinical experiences, commentaries, and conference abstracts.

### 2.2 Participants

Patients who were older than 18 years and diagnosed with AIS according to the recognized guidelines or criteria and confirmed by computed tomography (CT) or magnetic resonance imaging (MRI) were included without limitations on sex, culture, nationality, or race. The duration of the stroke should have been less than 14 days. We excluded patients with hypoxic-ischemic encephalopathy and *postpartum* apoplexy.

### 2.3 Types of interventions

The SXNI group received SXNI combined with conventional treatments (CTs), and the non-SXNI group received CTs alone or CTs combined with other injections. According to the Chinese guidelines for the diagnosis and treatment of AIS 2018; Chinese Society of Neurology Chinese Stroke Society, 2018), CTs include IVT, EVT, antiplatelet drugs, neuroprotective agents, and symptomatic supportive treatment, such as the management of blood pressure and blood lipid and sugar levels. Other injections were defined as any monodrug of non-Ginkgo biloba extract, such as compound Danshen injection, Shuxuetong injection, and so on. The duration of treatment had to be between 14 and 30 days. There were no restrictions on daily dose or frequency.

### 2.4 Outcome measures

The primary outcome was clinical efficacy assessed by the CER. According to the scoring criteria for clinical neurological deficit (CND) in stroke patients (Fourth Session of Chinese National Cerebrovascular Conference, 1996), improvements in CND were classified into five categories: a) recovery: 90%–100% reduction in CND with a disability level of 0; b) significant improvement: 46%–89% reduction in CND with a disability level of 1–3; c) progress: 18%–45% reduction in CND; d) inefficacy: less than 17% reduction in CND; e) deterioration: greater increase in CND. CER = (a + b + c) cases/total cases × 100% (National Administration of Traditional Chinese Medicine, 1994; Zheng, 2002).

The secondary outcomes included a) neurological function assessed by the Chinese Stroke Scale (CSS) and the NIHSS; b) ADL assessed by the Barthel Index (BI); c) AEs. The CSS consists of eight dimensions with a total score of 45. A higher CSS score indicates that a patient’s stroke is more severe (Fourth Session of Chinese National Cerebrovascular Conference, 1996). The NHISS consists of 15 items with a total score of 42 and is a validated tool for assessing the severity of acute stroke. The greater the NIHSS score is, the more severe the neurological deficit (Brott et al., 1989; Kwah and Diong, 2014). The highest BI score is 100, and the higher the BI score is, the better the individual’s ability to perform ADLs (Mahoney and Barthel, 1965). The common AEs related to SXNI are headache, flushing, and so on.

### 2.5 Data sources and searches

Seven databases were searched from their inception to January 2023, including PubMed, the Cochrane Library, Embase, the China National Knowledge Infrastructure (CNKI), the WanFang Database, the Chinese Scientific Journal Database (VIP), and the Chinese Biological Medicine Database (CBM). We also searched the Chinese Clinical Trial Registry (http://www.chictr.org.cn/) and the U.S. Clinical Trial Registry (https://clinicaltrials.gov/). We screened the reference lists of all included studies to identify more eligible studies. The search terms included (“stroke” OR “palsy” OR “apoplexy” OR “apoplexia” OR “cerebrovascular disorders” OR “cerebral infarction” OR “infarct of brain” OR “cerebral hemorrhage” OR “intracerebral hemorrhage” OR “brain hemorrhage”) AND (“shuxuening” OR “shuxuening injection” OR “Ginkgo Leaf” OR “Folium Ginkgo” OR “Chinese patent medicine”). The detailed search strategy is described in Supplementary Appendix 1.

### 2.6 Selection of studies

We not only removed reduplicated articles using NoteExpress 3.6 but also performed manual de-duplication. Then, two reviewers independently screened the literature based on predefined eligibility criteria. We first excluded irrelevant literature by reading the titles and abstracts and then reading the full texts to select eligible studies. When two or more studies reported the same RCT, we selected the study with the largest sample size, appropriate outcomes, and the earliest publication. We consulted a third reviewer to resolve any disagreements.

### 2.7 Data extraction and management

We developed standardized data extraction forms that included study information (e.g., author, publication year, city, and country), characteristics of participants (e.g., age, sex, and course of stroke), study design (e.g., sample size, interventions, administration methods, duration of intervention, and SXNI manufacturing company), and outcomes (e.g., CER, NIHSS, CSS, BI, and AEs). Two reviewers extracted the data independently and cross-checked the extracted data. We contacted the authors via email when the information in the article was unclear or insufficient. If there were disagreements on data extraction, a third reviewer was consulted.

### 2.8 Quality assessment

The quality of the included studies was evaluated by two reviewers using the Cochrane risk of bias (ROB) assessment tool (Higgins and Green, 2011). The ROB tool includes seven items: random sequence generation, allocation concealment, blinding of participants and personnel, blinding of outcome assessment, incomplete outcome data, selective reporting, and other biases. Each item can be judged as “low risk”, “high risk” or “unclear risk”. If a study had a “low risk” of more than four domains, its overall quality was judged as high. Any disagreements between the two reviewers were resolved by a third reviewer.

### 2.9 Data synthesis

We used R software (version 4.1.2, https://cran.r-project.org/) and Review Manager software (version 5.4, Cochrane Collaboration, Copenhagen, Denmark) to analyze the data. Dichotomous data are expressed as risk ratios (RRs) and 95% confidence intervals (CIs). Continuous data assessed using the same scale are expressed as weighted mean differences (WMDs) and 95% CIs, whereas standardized mean differences (SMDs) and 95% CIs were used for the different scales. Statistical heterogeneity was assessed by *I* ^*2*^ and *p* values from the Cochrane Q test. *I* ^*2*^ < 50% and *p* ≥ 0.05 indicated no heterogeneity among studies. A random effects model was used if heterogeneity existed; otherwise, the fixed effects model was used. *p* < 0.05 was considered to indicate a statistically significant difference.

### 2.10 Sensitivity analysis

We tested the stability of the meta-analysis results by alternating the random and fixed effects models. Moreover, we excluded each study in turn to test the robustness of the results.

### 2.11 Subgroup analysis

We performed a subgroup analysis based on the duration of intervention, daily dose, and different injections used in the control group. We also conducted mixed-effects meta-regression analysis using sample size (n ≤ 107 vs. n > 107), quality of studies (high vs. low), publication year, mean age (≤60 years old vs. > 60 years old vs. mixed), and total dose of SXNI (≤280 mL vs. > 280 mL) as covariates to search for sources of heterogeneity.

### 2.12 Publication bias

Publication bias was assessed with “funnel” and Egger’s tests when the number of included studies was more than 10.

### 2.13 Quality of evidence

We evaluated the quality of evidence for each outcome using the Grading of Recommendation, Assessment, Development, and Evaluation (GRADE) tool (https://www.gradepro.org/). The GRADE included five domains: a) risk of bias, b) inconsistency, c) indirectness, d) imprecision, and e) other considerations, such as publication bias and number of included studies. The overall level of evidence for every outcome was judged as high, moderate, low, or very low. Any disagreement regarding the level of evidence was resolved through discussion and consultation.

## 3 Results

### 3.1 Study selection

**FIGURE 1:**
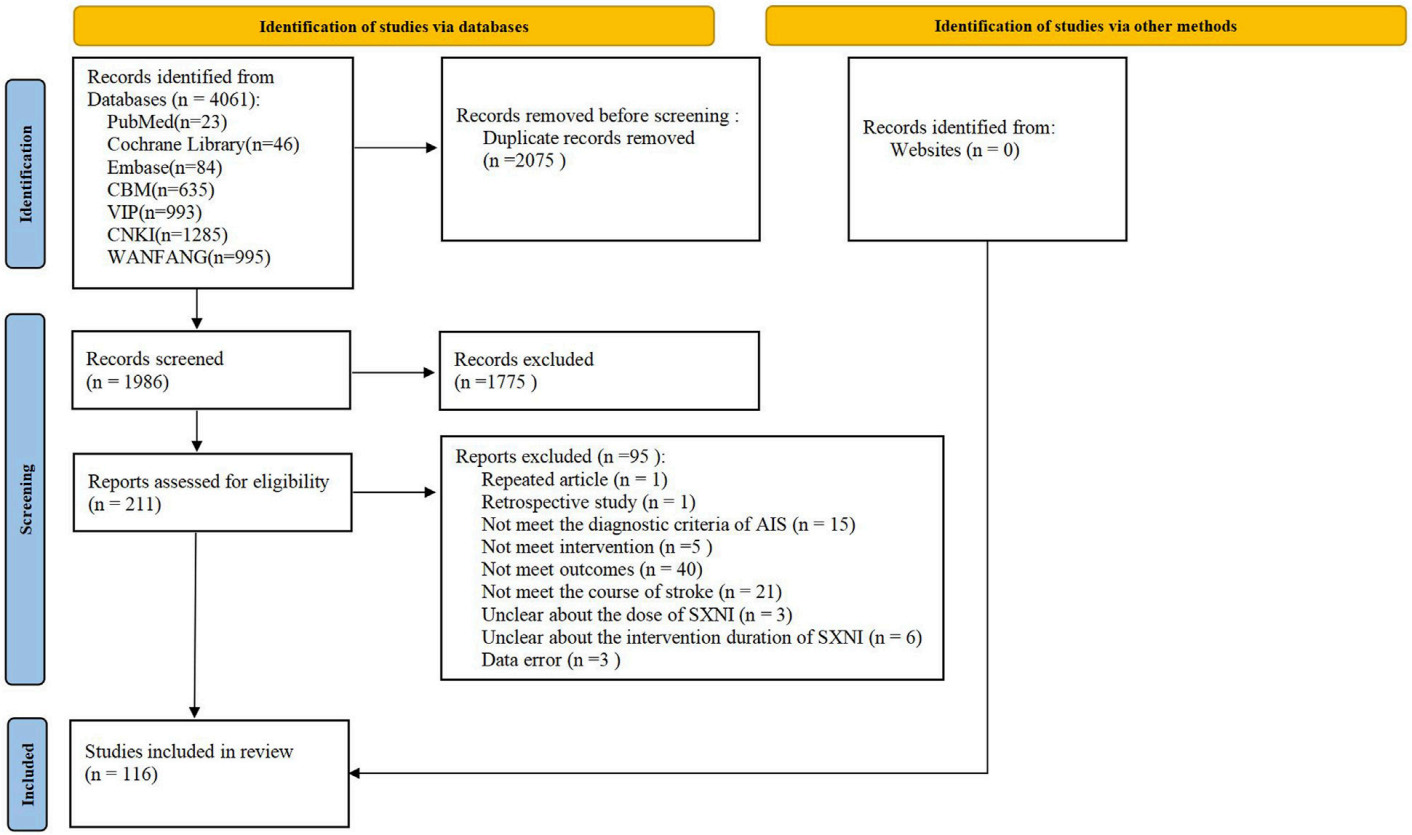
PRISMA flowchart of study selection.

A total of 4,061 records were retrieved, 2075 of which were duplicate publications. After removing 1,775 records that did not meet the criteria by screening the titles and abstracts, we read the full texts of 211 articles. Finally, 116 eligible studies were included in the meta-analysis. The details of the study selection process are displayed in Figure 1. The full citation details of the included and excluded studies are described in Supplementary Appendix 2.

### 3.2 Characteristics of the included studies

All included studies were conducted in China and published in Chinese, with publication years distributed between 2002 and 2023. The most frequent provinces of author affiliation were Henan, Shandong, Guangdong, and Hebei. The sample sizes were between 40 and 438, and the duration of AIS ranged from less than 12 h to 14 days. The intervention duration ranged from 14 to 30 days. The daily dose of SXNI varied from 2 to 30 mL. The total dose of SXNI was between 28 and 900 mL. Thirty-six studies with 3,568 participants compared the efficacy of SXNI plus CTs vs. CTs alone, and eight studies with 8,833 participants compared the efficacy of SXNI plus CTs vs. CTs plus other injections. Of the 116 studies, 102 reported the CER, 14 reported the BI, 44 reported the CSS, and 16 reported the NIHSS. The characteristics of the included studies are described in Table 1.

**TABLE 1:** Characteristics of the included RCTs. (Note. AEs, adverse events; BI, Barthel index; C, control group; CER, clinical effective rate; CSS, Chinese Stroke Scale; CTs, conventional treatments; d, day; F, female; h, hour; IVGTT, intravenous injection; M, male; NIHSS, National Institutes of Health Stroke Scale; NR, not reported; SXNI, shuxuening injection; T, treatment group; w, week.)

Study	Study sites	Sample size	Time since stroke		Age		Sex (M/F)		Interventions		Daily dose of SXNI	Intervention duration	SXNI manufacturing company	Outcomes
		T/C	T	C	T	C	T	C	T	C				
Cao M 2004	Hebei, China	35/40	11h-6d	12h-7d	41-78	43-76	22/13	26/14	SXNI + CTs	CTs + Compound Danshen injection	15 ml, qd, ivgtt	14 d	China Resources Double-crane Pharmaceutical Co., Ltd	CER, AEs
Cao XM 2015	Hebei, China	56/56	10–28h	10-28h	60 ± 1.2	60 ± 1.2	NR	NR	SXNI + CTs	CTs + Danshen injection	20 ml, qd, ivgtt	14 d	NR	CER, CSS
Chang CF 2015	Henan, China	150/128	6–72h	6-72h	64.9	63.7	98/52	84/44	SXNI + CTs	CTs + Compound Danshen injection	20 ml, qd, ivgtt	14 d	NR	CER, CSS, BI, AEs
Che YQ 2005	Liaoning, China	46/44	6–72h	6-72h	50-76	48-78	29/17	34/10	SXNI + CTs	CTs + Danshen injection	20 ml, qd, ivgtt	14 d	Xiamen Pengdao Pharmaceutical Co., Ltd	CER, CSS, AEs
Chen JJ 2014	Jiangsu, China	30/30	48h–1w	48h-1w	61 ± 5.26	62 ± 5.77	20/10	18/12	SXNI + CTs	CTs + Compound Danshen injection	20 ml, qd, ivgtt	14 d	NR	CER, AEs
Chen LY 2021	Hunan, China	35/35	<3d	<3d	57.84 ± 1.55	56.68 ± 1.39	20/15	18/17	SXNI + CTs	CTs + Edaravone injection	2 ml, qd, ivgtt	14 d	China Shineway Pharmaceutical Group Limited	NIHSS, BI
Chen R 2015	Jiangsu, China	39/39	32.47 ± 6.52h	32.47 ± 6.52h	65.38 ± 7.49	65.38 ± 7.49	NR	NR	SXNI + CTs	CTs + Compound Danshen injection	20 ml, qd, ivgtt	28 d	NR	CSS
Chen YF 2009	Shanghai, China	40/40	1-5d	1-5d	74.60 ± 7.53	72.95 ± 7.37	16/24	13/27	SXNI + CTs	CTs + Danshen injection	20 ml, qd, ivgtt	14 d	China Shineway Pharmaceutical Group Limited	CER
Chen ZC 2014	Guangdong, China	34/34	12-72 h	12-72 h	61.6 ± 4.3	58.6±5.3	22/12	18/16	SXNI + CTs	CTs + Chuanxiongqin injection	20 ml, qd, ivgtt	14 d	SHIYAO YINHU PHARMACENRTICAL CO., LTD	CER, AEs
Cheng ZL 2016	Hebei, China	40/40	15.4 ± 4.8h	16.1 ± 5.2h	58.7 ± 7.2	60.1 ± 6.8	25/15	23/17	SXNI + CTs	CTs	10 ml, qd, ivgtt	14 d	China Shineway Pharmaceutical Group Limited	CER
Cui YM 2014	Henan, China	62/62	≤48 h	≤48 h	62.8 ± 12.6	62.8 ± 12.6	NR	NR	SXNI + CTs	CTs + Venoruton injection	20 ml, qd, ivgtt	14 d	China Shineway Pharmaceutical Group Limited	CER
Dai YP 2008	Henan, China	80/80	l-7d	l-7d	38-82	40-81	44/36	47/33	SXNI + CTs	CTs + Venoruton injection	20 ml, qd, ivgtt	14 d	China Shineway Pharmaceutical Group Limited	CER, AEs
Du PK 2016	Henan, China	49/49	6.1 ± 1.9d	6.5 ± 1.4d	61.2 ± 3.7	63.1 ± 4.0	31/18	29/20	SXNI + CTs	CTs	20 ml, qd, ivgtt	14 d	Heilongjiang Zbd Pharmaceutical Co., Ltd	CER, CSS
Du XL 2010	Beijing, China	99/99	1.7 ± 0.5d	1.9 ± 0.3d	69.1 ± 4.4	67.3 ± 3.7	54/50	51/51	SXNI + CTs	CTs	20 ml, qd, ivgtt	14 d	China Shineway Pharmaceutical Group Limited	NIHSS, AEs
Feng JW 2017	Shandong, China	47/47	6.15 ± 1.29h	6.02 ± 1.26h	56.89 ± 12.67	58.05 ± 10.38	26/21	27/20	SXNI + CTs	CTs + Danshen injection	30 ml, qd, ivgtt	14 d	China Shineway Pharmaceutical Group Limited	CER
Gao YD 2011	Zhejiang, China	60/60	<72h	<72h	45-85	46-87	39/21	40/20	SXNI + CTs	CTs	20 ml, qd, ivgtt	14 d	NR	CER, CSS, AEs
Ge J 2013	Henan, China	70/67	6-24h	6-24h	61.23 ± 12.98	60.88 ± 11.75	39/31	38/29	SXNI + CTs	CTs + Compound Danshen injection	10 ml, qd, ivgtt	14 d	Langzhi Group Wanrong Pharmaceutical Co., Ltd.	CER, AEs
Guo WJ 2013	Hebei, China	35/35	l-3d	l-3d	58.38 ± 4.27	61.32 ± 5.88	20/15	19/16	SXNI + CTs	CTs	20 ml, qd, ivgtt	14 d	China Resources Double-crane Pharmaceutical Co., Ltd	CER, AEs
He J 2010	Anhui, China	40/40	1-3d	1-3d	58-72	59-74	32/8	28/12	SXNI + CTs	CTs + Compound Danshen injection	20 ml, qd, ivgtt	30 d	NR	CER, AEs
He JQ 2013	Guangdong, China	39/39	<12h	<12h	71.2 ± 2.3	72.1±2.1	30/9	31/8	SXNI + CTs	CTs	10 ml, qd, ivgtt	20 d	NR	CER
He XY 2006	Sichuan, China	30/30	<12h	<12h	78.69 ± 6.21	78.69 ± 6.21	NR	NR	SXNI + CTs	CTs	25 ml, qd, ivgtt	15 d	NR	CER, AEs
Hua GC 2002	Shanghai, China	60/32	36 ± 0.4h	35 ± 0.6h	69 ± 3.2	68 ± 4.1	36/24	17/15	SXNI + CTs	CTs + Ginaton injection	25 ml, qd, ivgtt	15 d	Beijing Wanhui Pharmaceutical Group	AEs
Huang DL 2009	Guangdong, China	40/40	12-96h	12-96h	38-72	39-71	27/13	28/12	SXNI + CTs	CTs + Compound Danshen injection	20 ml, qd, ivgtt	14 d	NR	CER, AEs
Huang JL 2010	Shaanxi, China	60/60	30 min-42 h	1-39 h	55.2 ± 8.5	56.6 ± 7.9	35/25	37/23	SXNI + CTs	CTs	20 ml, qd, ivgtt	28 d	Boan Brothers Pharmaceutical Co., Ltd	ESS, CER, BI
Huang M 2004	Guangdong, China	30/30	6 h-3 d	6 h-3 d	65.3 ± 7.5	64.2 ± 8.1	18/12	20/10	SXNI + CTs	CTs + Compound Danshen injection	10 ml, qd, ivgtt	14 d	Langzhi Group Wanrong Pharmaceutical Co., Ltd	CSS
Huang XZ 2014	Fujian, China	64/60	<14d	<14d	86.7 ± 3.6	87.7 ± 2.8	44/20	38/22	SXNI + CTs	CTs + Rhadiola extract injection	20 ml, qd, ivgtt	14 d	Beijing China Rescources High-Tech Natural Pharmaceutical Co., Ltd	CER, AEs
Jia HB 2008	Liaoning, China	62/61	12-72h	12-72h	43-85	42-86	39/23	37/24	SXNI + CTs	CTs + Chuanxiongqin injection	20 ml, qd, ivgtt	14 d	Beijing China Rescources High-Tech Natural Pharmaceutical Co., Ltd	CER, CSS, AEs
Jia HY 2021	Henan, China	41/41	11.52 ± 1.47h	11.74 ± 1.51h	57.38 ± 4.67	57.95 ± 4.16	22/19	23/18	SXNI + CTs	CTs	20 ml, qd, ivgtt	14 d	NR	NIHSS, CER
Jiang BN 2009	Shandong, China	55/55	<72h	<72h	67 ± 8.31	70 ± 9.14	35/20	33/22	SXNI + CTs	CTs + Xuesaitong injection	10 ml, qd, ivgtt	21 d	Tonghua Guhong Pharmaceutical Co., Ltd	CER, CSS
Jiao XL 2008	Zhejiang, China	30/22	3h-7d	3h-7d	46-89	46-89	NR	NR	SXNI + CTs	CTs + Compound Danshen injection	20 ml, qd, ivgtt	28 d	NR	CER
Ji DY 2014	Shaanxi, China	62/60	8-28h	7-29h	41-77	40-78	38/24	36/24	SXNI + CTs	CTs + Danshen injection	20 ml, qd, ivgtt	14 d	NR	CER, AEs
Kou XF 2012	Henan, China	50/50	<72h	<72h	66.7 ± 9.3	66.7±9.3	NR	NR	SXNI + CTs	CTs	20 ml, qd, ivgtt	14 d	China Shineway Pharmaceutical Group Limited	NIHSS, CER, AEs
Lei GR 2014	Jiangxi, China	45/45	18.3 ± 5.1h	18.3 ± 5.1h	59.21 ± 7.4	59.21 ± 7.4	NR	NR	SXNI + CTs	CTs + Danshen injection	20 ml, qd, ivgtt	14 d	NR	CER
Li JH 2008	Beijing, China	44/44	<48h	<48h	61-82	61-80	32/12	34/10	SXNI + CTs	CTs + Compound Danshen injection	20 ml, qd, ivgtt	14 d	NR	CER, CSS, AEs
Li SJ 2014	Shaanxi, China	60/60	≤72h	≤72h	63.8 ± 1.5	63.8 ± 1.5	NR	NR	SXNI + CTs	CTs + Danshen injection	20 ml, qd, ivgtt	14 d	NR	CER, CSS, BI, AEs
Li XF 2014	Shandong, China	48/42	2.1 ± 1.0h	2.2 ± 1.2h	63.7 ± 5.9	64.1 ± 6.3	26/22	22/20	SXNI + CTs	CTs	20 ml, qd, ivgtt	28 d	NR	CSS
Li XH 2011	Jilin, China	120/120	≤7d	≤7d	42-78	40-77	NR	NR	SXNI + CTs	CTs	20 ml, qd, ivgtt	14 d	SHIYAO YINHU PHARMACENRTICAL CO., LTD	CER, CSS
Li XJ 2010	Sichuan, China	28/30	3.78 ± 1.96d	4.08 ± 2.11d	63.5 ± 4.3	62.8 ± 4.6	18/10	19/11	SXNI + CTs	CTs + Xuesaitong injection	20 ml, qd, ivgtt	14 d	China Resources Double-crane Pharmaceutical Co., Ltd	CER, CSS
Li XL 2023	Jiangsu, China	35/35	<14d	<14d	63.79 ± 4.83	63.02 ± 4.57	23/12	25/10	SXNI + CTs	CTs	20 ml, qd, ivgtt	14 d	China Shineway Pharmaceutical Group Limited	NIHSS, CER, BI
Li XR 2011	Guizhou, China	90/60	<72h	<72h	50.8 ± 8.7	60.7 ± 9.5	39/51	26/34	SXNI + CTs	CTs	10 ml, qd, ivgtt	14 d	China Shineway Pharmaceutical Group Limited	CSS
Li YJ 2018	Hebei, China	62/62	6-72h	6-72h	58.6 ± 3.4	59.2 ± 3.5	32/30	34/28	SXNI + CTs	CTs	20 ml, qd, ivgtt	14 d	Beijing China Rescources High-Tech Natural Pharmaceutical Co., Ltd	NIHSS, CER, BI
Li ZY 2013	Neimenggu, China	80/80	9-72h	9-72h	41-76	41-76	NR	NR	SXNI + CTs	CTs + Troxerutin injection	20 ml, qd, ivgtt	15 d	NR	NIHSS, CER, AEs
Lin YQ 2006	Guangdong, China	50/50	48h-1w	48h-1w	35-79	40-82	27/23	30/20	SXNI + CTs	CTs + Venoruton injection	6 ml, qd, ivgtt	15 d	NR	CER, AEs
Ling YX 2006	Guangdong, China	220/218	8.2 ± 4.5h	8.3 ± 4.8h	56.5 ± 21.5	57.2 ± 22.4	124/96	120/98	SXNI + CTs	CTs + Compound Danshen injection	12 ml, qd, ivgtt	15 d	NR	CER, AEs
Liu JX 2004	Shaanxi, China	60/46	1-5d	1-6d	41-83	42-84	32/28	24/22	SXNI + CTs	CTs	30 ml, qd, ivgtt	30 d	Beijing China Rescources High-Tech Natural Pharmaceutical Co., Ltd.	CER
Liu JX 2010	Tianjin, China	50/50	<72h	<72h	45-75	45-75	NR	NR	SXNI + CTs	CTs + Xuesaitong injection	20 ml, qd, ivgtt	14 d	Beijing China Rescources High-Tech Natural Pharmaceutical Co., Ltd	CER, AEs
Liu MF 2009	Henan, China	60/60	≤3d	≤3d	63.9 ± 8.7	63.9 ± 8.7	NR	NR	SXNI + CTs	CTs + Compound Danshen injection	16 ml, qd, ivgtt	14 d	NR	CER, AEs
Liu S 2004	Shandong,China	60/60	1-3d	1-3d	45-78	45-78	34/26	38/22	SXNI + CTs	CTs + Venoruton injection	12 ml, qd, ivgtt	14 d	Sanjiu Enterprise Group(Shenzhen Nanfang Pharmaceutical Plant)	AEs
Liu XH 2013	Hunan, China	82/82	≤12h	≤12h	77.8±9.2	76.5±8.7	75/7	76/6	SXNI + CTs	CTs + Compound Danshen injection	25 ml, qd, ivgtt	30 d	NR	CER
Liu XJ 2008	Liaoning, China	68/30	≤7d	≤7d	36-80	36-80	41/27	17/13	SXNI + CTs	CTs + Danshen injection	20 ml, qd, ivgtt	14 d	NR	CER, AEs
Liu XY 2016	Neimenggu, China	47/47	4.92 ± 1.21h	4.86 ± 1.12h	64.39 ± 9.26	64.28 ± 9.17	27/20	25/22	SXNI + CTs	CTs + Shuxuetong injection	20 ml, qd, ivgtt	14 d	China Shineway Pharmaceutical Group Limited	CER, CSS
Liu Y 2003	Jiangsu, China	56/48	≤48h	≤48h	<75	<75	NR	NR	SXNI + CTs	CTs + Danshen injection	20 ml, qd, ivgtt	14 d	China Resources Double-crane Pharmaceutical Co., Ltd	CER
Ma YX 2016	Jilin, China	43/43	2.18 ± 0.41d	2.34 ± 0.42d	60.34 ± 5.28	60.23 ± 5.32	24/19	23/20	SXNI + CTs	CTs + Danshen injection	20 ml, qd, ivgtt	14 d	NR	CER, CSS, BI
Mai Y 2005	Yunnan, China	34/30	1-5d	1-5d	61-84	63-86	28/6	25/5	SXNI + CTs	CTs	20 ml, qd, ivgtt	30 d	Langzhi Group Wanrong Pharmaceutical Co., Ltd	CER, CSS, AEs
Nie XP 2020	Jiangxi, China	44/44	4.62 ± 1.79h	4.57 ± 1.62h	66.8 ± 2.5	66.4 ± 2.6	22/22	20/24	SXNI + CTs	CTs	20 ml, qd, ivgtt	20 d	Beijing China Rescources High-Tech Natural Pharmaceutical Co., Ltd	CER, AEs
Peng J 2021	Hunan, China	37/37	4.21 ± 0.57h	4.39 ± 0.66h	64.25 ± 0.37	64.53 ± 0.84	20/17	21/16	SXNI + CTs	CTs	20 ml, qd, ivgtt	14 d	Shanxi Taiyuan Pharmaceutical Co., Ltd	NIHSS, BI
Qin DY 2014	Guangxi, China	30/30	6-24h	6-24h	42-78	45-76	20/10	18/12	SXNI + CTs	CTs	20 ml, qd, ivgtt	14 d	China Shineway Pharmaceutical Group Limited	CER, CSS
Qin QA 2013	Henan, China	120/116	9-28h	10-29h	41-75	40-76	76/44	70/46	SXNI + CTs	CTs + Danshen injection	20 ml, qd, ivgtt	14 d	NR	CER, CSS, AEs
Ren JM 2006	Jiangsu, China	38/34	≤48h	≤48h	64±8	62±7	20/18	18/16	SXNI + CTs	CTs + Danshen injection	20 ml, qd, ivgtt	14 d	NR	CER, AEs
Shang S 2014	Henan, China	44/44	<72h	<72h	37-81	36-79	27/17	26/18	SXNI + CTs	CTs + Compound Danshen injection	20 ml, qd, ivgtt	15 d	NR	CER, AEs
Shen DD 2017	Zhejiang, China	57/57	16.1 ± 4.12h	15.67 ± 3.78h	62.34 ± 4.01	61.46 ± 3.69	33/24	37/20	SXNI + CTs	CTs	20 ml, qd, ivgtt	14 d	NR	NIHSS, CER, AEs
Shi JP 2008	Henan, China	41/41	48-72h	48-72h	40-79	37-78	26/15	24/17	SXNI + CTs	CTs + Compound Danshen injection	20 ml, qd, ivgtt	15 d	Guizhou Yibai Pharmaceutical Co., Ltd	CER, CSS, AEs
Su HM 2007	Shanghai, China	36/32	8h-6d	9h-5d	53.5	55	22/14	21/11	SXNI + CTs	CTs + Danshen injection	20 ml, qd, ivgtt	15 d	NR	CER
Sun BJ 2014	Hebei, China	100/100	36.5 ± 13.5h	39.5 ± 16.3h	60.5 ± 14.6	63.5 ± 14.6	55/45	54/46	SXNI + CTs	CTs + Shuxuetong injection	20 ml, qd, ivgtt	14 d	NR	CER, CSS
Sun HY 2005	Zhejiang, China	52/51	9h-3d	9h-3d	39-78	41-77	34/18	32/19	SXNI + CTs	CTs + Danshen injection	12 ml, qd, ivgtt	15 d	Langzhi Group Wanrong Pharmaceutical Co., Ltd.	CER, CSS, AEs
Sun T 2019	XinJiang, China	20/20	≤24h	≤24h	59.4 ± 6.7	59.4 ± 6.7	NR	NR	SXNI + CTs	CTs + Compound Xueshuantong injection	20 ml, qd, ivgtt	14 d	Heilongjiang Zbd Pharmaceutical Co., Ltd	NIHSS, CER, AEs
Tai SEGL 2011	Xinjiang, China	60/60	≤3d	≤3d	58-86	56-86	48/12	47/13	SXNI + CTs	CTs + Compound Danshen injection	20 ml, qd, ivgtt	30 d	NR	CER, CSS
Tian ZC 2008	Shandong, China	56/24	1-10d	1-10d	62 ± 3.72	64.1 ± 3.86	NR	NR	SXNI + CTs	CTs + Danshen injection	20 ml, qd, ivgtt	21 d	NR	CSS, AEs
Wang DH 2023	Beijing, China	60/60	26.14 ± 5.32h	25.32 ± 4.94h	57.32 ± 8.56	58.59 ± 7.88	35/25	33/27	SXNI + CTs	CTs	20 ml, qd, ivgtt	14 d	SHIYAO YINHU PHARMACENRTICAL CO., LTD	NIHSS, CER, BI, AEs
Wang HL 2013	Beijing, China	40/40	19.1 ± 10.6h	18.6 ± 9.9h	66.7 ± 8.5	67.4 ± 9.1	22/18	23/17	SXNI + CTs	CTs + Edaravone injection	20 ml, qd, ivgtt	14 d	Heilongjiang Zbd Pharmaceutical Co., Ltd.	NIHSS, CER
Wang HR 2010	Chongqing, China	53/53	1-65h	0.5-61h	43-77	41-78	24/29	21/32	SXNI + CTs	CTs	20 ml, qd, ivgtt	28 d	Boan Brothers Pharmaceutical Co., Ltd	CER, CSS
Wang JF 2018	Shandong, China	32/32	15.27±4.24h	15.34±4.11h	70.41±1.33	70.34 ± 1.41	21/11	20/12	SXNI + CTs	CTs	20 ml, qd, ivgtt	14 d	SHIYAO YINHU PHARMACENRTICAL CO., LTD	CER
Wang MS 2012	Henan, China	106/106	<48h	<48h	67.4 ± 8.7	69.0 ± 9.8	70/36	68/38	SXNI + CTs	CTs	20 ml, qd, ivgtt	14 d	NR	CER, AEs
Wang QY 2012	Shanxi, China	40/40	<72h	<72h	48-67	44-70	27/13	25/15	SXNI + CTs	CTs + Compound Danshen injection	20 ml, qd, ivgtt	14 d	NR	CER, CSS, AEs
Wang SW 2014	Guangxi, China	31/30	0.5-7d	1-7d	49-78	50-77	20/11	21/9	SXNI + CTs	CTs + Compound Danshen injection	20 ml, qd, ivgtt	15 d	NR	CER, CSS, AEs
Wang WH 2006	Sichuan, China	30/30	<12h	<12h	78.69 ± 6.21	78.69 ± 6.21	NR	NR	SXNI + CTs	CTs	25 ml, qd, ivgtt	15 d	NR	CER, AEs
Wang XH 2015	Henan, China	51/51	4.01 ± 0.22d	4.01 ± 0.22d	62.1 ± 2.3	62.1 ± 2.3	NR	NR	SXNI + CTs	CTs + Xuesaitong injection	20 ml, qd, ivgtt	14 d	China Resources Double-crane Pharmaceutical Co., Ltd	CER, CSS
Wu J 2011	Henan, China	33/31	6h-3d	6h-3d	55.42 ± 11.72	56.03 ± 12.49	17/16	15/16	SXNI + CTs	CTs + Edaravone injection	16 ml, qd, ivgtt	14 d	Langzhi Group Wanrong Pharmaceutical Co., Ltd	CER, CSS
Wu XJ 2006	Hunan, China	30/30	1-3d	1-3d	65.80 ± 8.62	65.40 ± 8.40	16/14	17/13	SXNI + CTs	CTs + Compound Danshen injection	20 ml, qd, ivgtt	14 d	Shanxi Zhendong Taisheng Pharmaceutical Co., Ltd	CER, AEs
Wu Y 2011	Guangdong, China	30/30	6-72h	6-72h	60.21 ± 8.72	60.21 ± 8.72	NR	NR	SXNI + CTs	CTs	10 ml, qd, ivgtt	14 d	Langzhi Group Wanrong Pharmaceutical Co., Ltd	CER, AEs
Wu ZX 2004	Zhejiang, China	50/34	1-2d	1-2d	36-70	40-72	27/23	15/19	SXNI + CTs	CTs + Danshen injection	20 ml, qd, ivgtt	14 d	Langzhi Group Wanrong Pharmaceutical Co., Ltd	CER, AEs
Xiao DF 2022	Liaoning, China	40/40	18.02 ± 2.57h	17.96 ± 2.64h	66.84 ± 4.93	67.03 ± 5.22	21/19	22/18	SXNI + CTs	CTs	20 ml, qd, ivgtt	14 d	Heilongjiang Zbd Pharmaceutical Co., Ltd	NIHSS, CER, BI, AEs
Xie RP 2010	Shanxi, China	40/40	12-72h	12-72h	42-80	42-80	NR	NR	SXNI + CTs	CTs + Compound Danshen injection	20 ml, qd, ivgtt	15 d	China Shineway Pharmaceutical Group Limited	CER, AEs
Xie YQ 2004	Hunan, China	30/30	<7d	<7d	59.4	57.7	17/13	18/12	SXNI + CTs	CTs	25 ml, qd, ivgtt	15 d	China Resources Double-crane Pharmaceutical Co., Ltd	CER, CSS, BI, AEs
Xin FB 2005	Hubei, China	121/121	<24h	<24h	58 ± 8	60 ± 7	76/45	71/50	SXNI + CTs	CTs + Danshen injection	20 ml, qd, ivgtt	14 d	Langzhi Group Wanrong Pharmaceutical Co., Ltd	CER, CSS, AEs
Xiong SC 2011	Sichuan, China	40/40	5-22h	4-24h	51-75	50-72	22/28	23/17	SXNI + CTs	CTs + Danshen injection	20 ml, qd, ivgtt	20 d	NR	CER, CSS
Xu CY 2012	Shandong, China	60/60	<7d	<7d	41-82	40-81	32/28	38/22	SXNI + CTs	CTs + Compound Danshen injection	20 ml, qd, ivgtt	14 d	SHANGHAI ASIA PIONEER PHARMACENRTICAL CO., LTD	AEs
Xu L 2008	Henan, China	30/30	8h-1w	8h-1w	55.42 ± 11.72	56.03 ± 12.49	18/12	19/11	SXNI + CTs	CTs + Sodium Ozagrel injection	16 ml, qd, ivgtt	14 d	Langzhi Group Wanrong Pharmaceutical Co., Ltd	CER, CSS, AEs
Xu PF 2011	Guangdong, China	35/35	1-5d	1.05-5d	65 ± 9.2	66.8 ± 10.3	19/16	20/15	SXNI + CTs	CTs + Compound Danshen injection	20 ml, qd, ivgtt	14 d	Heilongjiang Zbd Pharmaceutical Co., Ltd	CER, AEs
Xue JY 2013	Henan, China	24/24	4.5 ± 1.8h	4.5 ± 1.8h	65.8 ± 5.2	65.8 ± 5.2	NR	NR	SXNI + CTs	CTs + Danshen injection	20 ml, qd, ivgtt	14 d	NR	CER
Yan TQ 2016	Shandong, China	45/45	12.4 ± 1.6h	14.1 ± 1.8h	54.2 ± 2.4	53.5 ± 2.5	25/20	23/22	SXNI + CTs	CTs	20 ml, qd, ivgtt	14 d	NR	CER
Yan XY 2010	Anhui, China	30/30	6-72h	6-72h	52.13 ± 9.71	58.32 ± 8.79	18/12	16/14	SXNI + CTs	CTs	20 ml, qd, ivgtt	14 d	China Shineway Pharmaceutical Group Limited	CER, AEs
Yang JH 2010	Hunan, China	48/48	<7d	<7d	46-75	47-75	26/22	25/23	SXNI + CTs	CTs	20 ml, qd, ivgtt	14 d	NR	CER, AEs
Yang M 2009	Anhui,China	30/30	<7d	<7d	66.3 ± 9.3	67.4 ± 8.6	NR	NR	SXNI + CTs	CTs + Sodium Ozagrel injection	20 ml, qd, ivgtt	14 d	NR	CER, AEs
Yang XW 2006	Guangdong, China	46/35	<48h	<48h	46-74	45-75	28/18	21/14	SXNI + CTs	CTs + Compound Xueshuantong injection	10 ml, qd, ivgtt	14 d	Langzhi Group Wanrong Pharmaceutical Co., Ltd	CER, AEs
Yin HX 2004	Guangdong, China	95/90	<48h	<48h	35-78	38-76	68/27	65/25	SXNI + CTs	CTs + Compound Danshen injection	10 ml, qd, ivgtt	14 d	Langzhi Group Wanrong Pharmaceutical Co., Ltd	CER, CSS, AEs
Yin ZL 2020	Jilin, China	34/34	12.46 ± 1.61h	12.48 ± 1.62h	56.17 ± 3.24	56.14 ± 3.26	20/14	19/15	SXNI + CTs	CTs	20 ml, qd, ivgtt	14 d	Tonghua Guhong Pharmaceutical Co., Ltd	NIHSS, CER
Yu BQ 2003	Jiangsu, China	66/75	11h-6d	11h-7d	67 ± 11.5	64 ± 12.4	42/36	49/41	SXNI + CTs	CTs + Compound Danshen injection	20 ml, qd, ivgtt	14 d	NR	CER, CSS, BI, AEs
Zang ZX 2010	Heilongjiang, China	65/62	9-72h	8-72h	62.5 ± 10.3	63.4 ± 9.89	41/24	38/24	SXNI + CTs	CTs + Danshen injection	20 ml, qd, ivgtt	14 d	China Shineway Pharmaceutical Group Limited	CER, CSS
Zhang GJ 2010	Heilongjiang, China	124/122	12-72h	12-72h	41-83	41-83	78/46	74/48	SXNI + CTs	CTs + Chuanxiongqin injection	20 ml, qd, ivgtt	14 d	NR	AEs
Zhang H 2009	Hubei, China	60/58	9-28h	10-29h	42-76	41-77	38/22	35/23	SXNI + CTs	CTs + Danshen injection	20 ml, qd, ivgtt	14 d	Beijing China Rescources High-Tech Natural Pharmaceutical Co., Ltd	CER, CSS, BI, AEs
Zhang HM 2004	Shandong, China	74/70	<7d	<7d	54-80	56-79	NR	NR	SXNI + CTs	CTs + Compound Danshen injection	20 ml, qd, ivgtt	14 d	NR	CER, CSS, AEs
Zhang HX 2006	Hebei, China	110/100	16.1 ± 8.4h	16 ± 8.5h	62.56 ± 12.37	62.52 ± 12.4	56/54	51/49	SXNI + CTs	CTs + Venoruton injection	20 ml, qd, ivgtt	15 d	China Resources Double-crane Pharmaceutical Co., Ltd	ESS, AEs
Zhang L 2007	Jilin, China	40/40	<72h	<72h	44-78	44-78	NR	NR	SXNI + CTs	CTs + Xuesaitong injection	10 ml, qd, ivgtt	14 d	NR	CER, AEs
Zhang L 2010	Henan, China	40/40	48h-1w	48h-1w	35-74	42-78	23/17	24/16	SXNI + CTs	CTs + Venoruton injection	6 ml, qd, ivgtt	15 d	NR	CER, AEs
Zhang MX 2018	Shanghai, China	40/40	<48h	<48h	43-81	43-81	26/14	26/14	SXNI + CTs	CTs + Compound Danshen injection	20 ml, qd, ivgtt	14 d	NR	CER, CSS
Zhang RL 2008	Shaanxi, China	67/62	1-6d	NR	36-75	36-75	46/21	44/18	SXNI + CTs	CTs	20 ml, qd, ivgtt	30 d	NR	CER, CSS, AEs
Zhang XK 2017	Shandong, China	62/62	<3d	<3d	58.66 ± 7.25	57.59 ± 9.31	32/30	33/29	SXNI + CTs	CTs + Danshen injection	20 ml, qd, ivgtt	14 d	NR	CER, CSS, AEs
Zhang XZ 2018	Henan, China	48/48	18.26 ± 2.32h	18.29 ± 2.30h	65.16 ± 4.62	65.19 ± 4.60	23/25	24/24	SXNI + CTs	CTs	20 ml, qd, ivgtt	14 d	China Shineway Pharmaceutical Group Limited	NIHSS, CER
Zhang YP 2004	Shanxi, China	66/58	6h-5d	6h-5d	45-78	42-80	38/28	35/23	SXNI + CTs	CTs + Compound Danshen injection	15 ml, qd, ivgtt	14 d	NR	CER, CSS
Zhao YX 2015	Henan, China	42/42	32.5 ± 9.4h	32.5 ± 9.4h	64.5 ± 8.1	64.5±8.1	NR	NR	SXNI + CTs	CTs + Edaravone injection	20 ml, qd, ivgtt	28 d	China Shineway Pharmaceutical Group Limited	NIHSS, BI, AEs
Zheng YZ 2014	Henan, China	36/36	<72h	<72h	47-76	47-76	NR	NR	SXNI + CTs	CTs + Xueshuantong injection	10 ml, bid, ivgtt	21 d	Datong Huida Pharmaceutical Co., Ltd	CER, AEs
Zhu L 2014	Shaanxi, China	73/73	5-24h	6-24h	62.5 ± 13.1	61.8 ± 12.5	40/33	38/35	SXNI + CTs	CTs + Compound Danshen injection	20 ml, qd, ivgtt	14 d	NR	CER, CSS, AEs
Zhu XJ 2013	Liaoning, China	51/30	18.02 ± 4.28h	18.02 ± 4.28h	62.41 ± 4.24	60.38 ± 6.02	21/30	18/12	SXNI + CTs	CTs	25 ml, qd, ivgtt	14 d	China Resources Double-crane Pharmaceutical Co., Ltd	CER
Zhuang JS 2004	Hebei, China	77/71	15-72h	15-72h	64.2 ± 9.7	65.4 ± 8.1	NR	NR	SXNI + CTs	CTs + Venoruton injection	20 ml, qd, ivgtt	15 d	NR	CER, AEs
Zi XH 2004	Hunan, China	28/26	<7d	<7d	69.2 ± 5.3	70 ± 6.6	NR	NR	SXNI + CTs	CTs + Sodium Ozagrel injection	25 ml, qd, ivgtt	15 d	NR	CER, AEs

Note: AEs, adverse events; BI, Barthel index; C, control group; CER, clinical effective rate; CSS, Chinese Stroke Scale; CTs, conventional treatments; d, day; F, female; h, hour; ivgtt, intravenous injection; M, male; NIHSS, National Institutes of Health Stroke Scale; NR, not report; SXNI, shuxuening injection; T, treatment group; w, week.

### 3.3 The quality of the included studies

**FIGURE 2:**
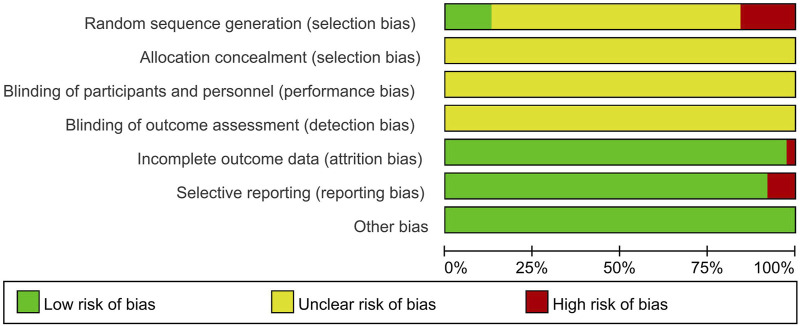
Risk of bias graph.

Fifteen (12.9%) studies were judged to be of moderate to high quality (Table 2; Figure 2). Of 116 studies, 110 (94.8%) mentioned the word “randomization”, 15 (12.9%) used random number table methods, and the remaining studies used incorrect or undetailed randomization methods. Five (4.3%) studies mentioned the word “blinding” without a detailed description of the specific methods. None of the studies reported allocation concealment. Three (2.6%) studies were judged as high risk because they did not conduct intention-to-treat analysis and did not specify the reason for case dropout. Nine (7.8%) studies were assessed as high risk for selective reporting based on the description of the methods in the article. All included studies were free of other sources of ROB.

**TABLE 2:** Quality assessment of the included studies using the Cochrane risk of bias (ROB) assessment tool^a^.

Author, year	Random sequence generation	Allocation concealment	Blinding of participants and personnel	Blinding of outcome assessment	Incomplete outcome data	Selective reporting	Other bias	Overall quality
Cao M 2004	unclear	unclear	unclear	unclear	low	low	low	low
Cao XM 2015	unclear	unclear	unclear	unclear	low	low	low	low
Chang CF 2015	unclear	unclear	unclear	unclear	low	low	low	low
Che YQ 2005	unclear	unclear	unclear	unclear	low	low	low	low
Chen JJ 2014	unclear	unclear	unclear	unclear	low	low	low	low
Chen LY 2021	low	unclear	unclear	unclear	low	low	low	high
Chen R 2015	high	unclear	unclear	unclear	low	low	low	low
Chen YF 2009	unclear	unclear	unclear	unclear	low	low	low	low
Chen ZC 2014	low	unclear	unclear	unclear	low	low	low	high
Cheng ZL 2016	unclear	unclear	unclear	unclear	low	low	low	low
Cui YM 2014	unclear	unclear	unclear	unclear	low	low	low	low
Dai YP 2008	unclear	unclear	unclear	unclear	low	low	low	low
Du PK 2016	unclear	unclear	unclear	unclear	low	low	low	low
Du XL 2010	high	unclear	unclear	unclear	low	low	low	low
Feng JW 2017	low	unclear	unclear	unclear	low	low	low	high
Gao YD 2011	unclear	unclear	unclear	unclear	low	low	low	low
Ge J 2013	unclear	unclear	unclear	unclear	low	low	low	low
Guo WJ 2013	unclear	unclear	unclear	unclear	low	low	low	low
He J 2010	unclear	unclear	unclear	unclear	low	low	low	low
He JQ 2013	unclear	unclear	unclear	unclear	low	low	low	low
He XY 2006	unclear	unclear	unclear	unclear	low	low	low	low
Hua GC 2002	unclear	unclear	unclear	unclear	low	low	low	low
Huang DL 2009	high	unclear	unclear	unclear	low	low	low	low
Huang JL 2010	low	unclear	unclear	unclear	low	low	low	high
Huang M 2004	unclear	unclear	unclear	unclear	low	low	low	low
Huang XZ 2014	high	unclear	unclear	unclear	low	low	low	low
Ji DY 2014	unclear	unclear	unclear	unclear	low	low	low	low
Jia HB 2008	unclear	unclear	unclear	unclear	low	low	low	low
Jia HY 2021	unclear	unclear	unclear	unclear	low	low	low	low
Jiang BN 2009	unclear	unclear	unclear	unclear	low	low	low	low
Jiao XL 2008	unclear	unclear	unclear	unclear	low	low	low	low
Kou XF 2012	high	unclear	unclear	unclear	low	low	low	low
Lei GR 2014	unclear	unclear	unclear	unclear	low	low	low	low
Li JH 2008	unclear	unclear	unclear	unclear	low	low	low	low
Li SJ 2014	high	unclear	unclear	unclear	low	low	low	low
Li XF 2014	high	unclear	unclear	unclear	low	low	low	low
Li XH 2011	unclear	unclear	unclear	unclear	low	low	low	low
Li XJ 2010	unclear	unclear	unclear	unclear	low	low	low	low
Li XL 2023	low	unclear	unclear	unclear	low	low	low	high
Li XR 2011	unclear	unclear	unclear	unclear	low	low	low	low
Li YJ 2018	low	unclear	unclear	unclear	low	low	low	high
Li ZY 2013	high	unclear	unclear	unclear	low	low	low	low
Lin YQ 2006	unclear	unclear	unclear	unclear	low	low	low	low
Ling YX 2006	unclear	unclear	unclear	unclear	low	low	low	low
Liu JX 2004	unclear	unclear	unclear	unclear	low	low	low	low
Liu JX 2010	unclear	unclear	unclear	unclear	low	low	low	low
Liu MF 2009	unclear	unclear	unclear	unclear	low	high	low	low
Liu S 2004	high	unclear	unclear	unclear	low	low	low	low
Liu XH 2013	unclear	unclear	unclear	unclear	low	low	low	low
Liu XJ 2008	unclear	unclear	unclear	unclear	low	low	low	low
Liu XY 2016	low	unclear	unclear	unclear	low	low	low	high
Liu Y 2003	unclear	unclear	unclear	unclear	low	high	low	low
Ma YX 2016	unclear	unclear	unclear	unclear	low	low	low	low
Mai Y 2005	unclear	unclear	unclear	unclear	low	high	low	low
Nie XP 2020	unclear	unclear	unclear	unclear	low	low	low	low
Peng J 2021	low	unclear	unclear	unclear	low	low	low	high
Qin DY 2014	unclear	unclear	unclear	unclear	low	low	low	low
Qin QA 2013	unclear	unclear	unclear	unclear	low	low	low	low
Ren JM 2006	unclear	unclear	unclear	unclear	low	low	low	low
Shang S 2014	unclear	unclear	unclear	unclear	low	low	low	low
Shen DD 2017	low	unclear	unclear	unclear	low	low	low	high
Shi JP 2008	unclear	unclear	unclear	unclear	low	low	low	low
Su HM 2007	unclear	unclear	unclear	unclear	low	low	low	low
Sun BJ 2014	unclear	unclear	unclear	unclear	low	low	low	low
Sun HY 2005	high	unclear	unclear	unclear	low	low	low	low
Sun T 2019	unclear	unclear	unclear	unclear	low	low	low	low
Tai SEGL 2011	unclear	unclear	unclear	unclear	low	high	low	low
Tian ZC 2008	unclear	unclear	unclear	unclear	low	low	low	low
Wang DH 2023	low	unclear	unclear	unclear	low	low	low	high
Wang HL 2013	unclear	unclear	unclear	unclear	low	low	low	low
Wang HR 2010	low	unclear	unclear	unclear	low	low	low	high
Wang JF 2018	unclear	unclear	unclear	unclear	low	low	low	low
Wang MS 2012	unclear	unclear	unclear	unclear	low	low	low	low
Wang QY 2012	high	unclear	unclear	unclear	low	high	low	low
Wang SW 2014	unclear	unclear	unclear	unclear	low	low	low	low
Wang WH 2006	unclear	unclear	unclear	unclear	low	low	low	low
Wang XH 2015	high	unclear	unclear	unclear	low	low	low	low
Wu J 2011	low	unclear	unclear	unclear	low	low	low	high
Wu XJ 2006	unclear	unclear	unclear	unclear	low	low	low	low
Wu Y 2011	unclear	unclear	unclear	unclear	low	high	low	low
Wu ZX 2004	unclear	unclear	unclear	unclear	low	low	low	low
Xiao DF 2022	low	unclear	unclear	unclear	low	low	low	high
Xie RP 2010	unclear	unclear	unclear	unclear	low	low	low	low
Xie YQ 2004	unclear	unclear	unclear	unclear	low	low	low	low
Xin FB 2005	unclear	unclear	unclear	unclear	low	low	low	low
Xiong SC 2011	unclear	unclear	unclear	unclear	low	low	low	low
Xu CY 2012	unclear	unclear	unclear	unclear	low	low	low	low
Xu L 2008	unclear	unclear	unclear	unclear	low	low	low	low
Xu PF 2011	unclear	unclear	unclear	unclear	low	low	low	low
Xue JY 2013	unclear	unclear	unclear	unclear	low	low	low	low
Yan TQ 2016	unclear	unclear	unclear	unclear	low	low	low	low
Yan XY 2010	high	unclear	unclear	unclear	low	low	low	low
Yang JH 2010	low	unclear	unclear	unclear	low	low	low	high
Yang M 2009	unclear	unclear	unclear	unclear	low	low	low	low
Yang XW 2006	unclear	unclear	unclear	unclear	low	low	low	low
Yin HX 2004	unclear	unclear	unclear	unclear	low	low	low	low
Yin ZL 2020	low	unclear	unclear	unclear	low	low	low	high
Yu BQ 2003	high	unclear	unclear	unclear	low	low	low	low
Zang ZX 2010	unclear	unclear	unclear	unclear	high	high	low	low
Zhang GJ 2010	unclear	unclear	unclear	unclear	low	low	low	low
Zhang H 2009	high	unclear	unclear	unclear	high	low	low	low
Zhang HM 2004	high	unclear	unclear	unclear	high	low	low	low
Zhang HX 2006	unclear	unclear	unclear	unclear	low	low	low	low
Zhang L 2007	unclear	unclear	unclear	unclear	low	low	low	low
Zhang L 2010	unclear	unclear	unclear	unclear	low	high	low	low
Zhang MX 2018	unclear	unclear	unclear	unclear	low	low	low	low
Zhang RL 2008	unclear	unclear	unclear	unclear	low	high	low	low
Zhang XK 2017	unclear	unclear	unclear	unclear	low	low	low	low
Zhang XZ 2018	unclear	unclear	unclear	unclear	low	low	low	low
Zhang YP 2004	high	unclear	unclear	unclear	low	low	low	low
Zhao YX 2015	unclear	unclear	unclear	unclear	low	low	low	low
Zheng YZ 2014	unclear	unclear	unclear	unclear	low	low	low	low
Zhu L 2014	unclear	unclear	unclear	unclear	low	low	low	low
Zhu XJ 2013	unclear	unclear	unclear	unclear	low	low	low	low
Zhuang JS 2004	unclear	unclear	unclear	unclear	low	low	low	low
Zi XH 2004	high	unclear	unclear	unclear	low	low	low	low

^a^ Higgins, J.P.T., Green, S., 2011. Cochrane Handbook for Systematic Reviews of Interventions. London, The Cochrane Collaboration.

### 3.4 Primary outcome

#### 3.4.1 Clinical efficacy

##### 3.4.1.1 SXNI plus CTs vs. CTs alone

**FIGURE 3:**
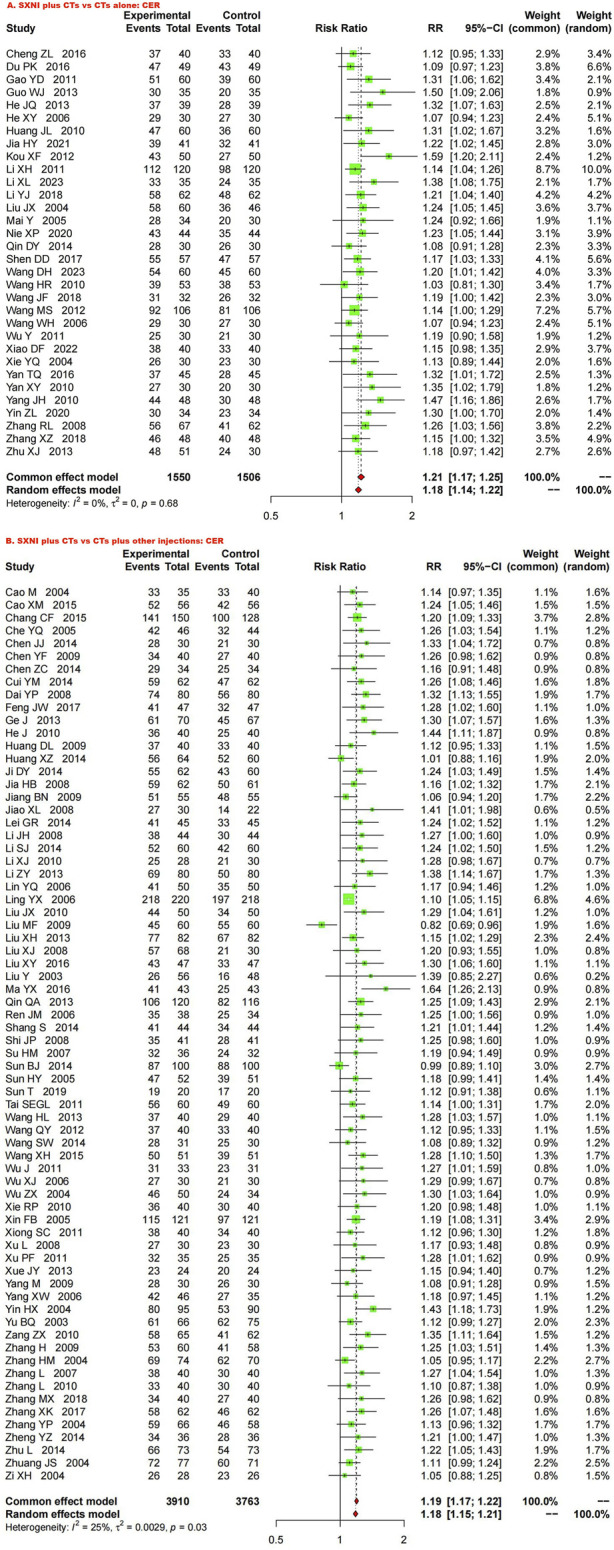
Forest plot and meta-analysis of clinical efficacy. (Note: CER, clinical effective rate; CTs, conventional treatments; SXNI, shuxuening injection).

**FIGURE 4:**
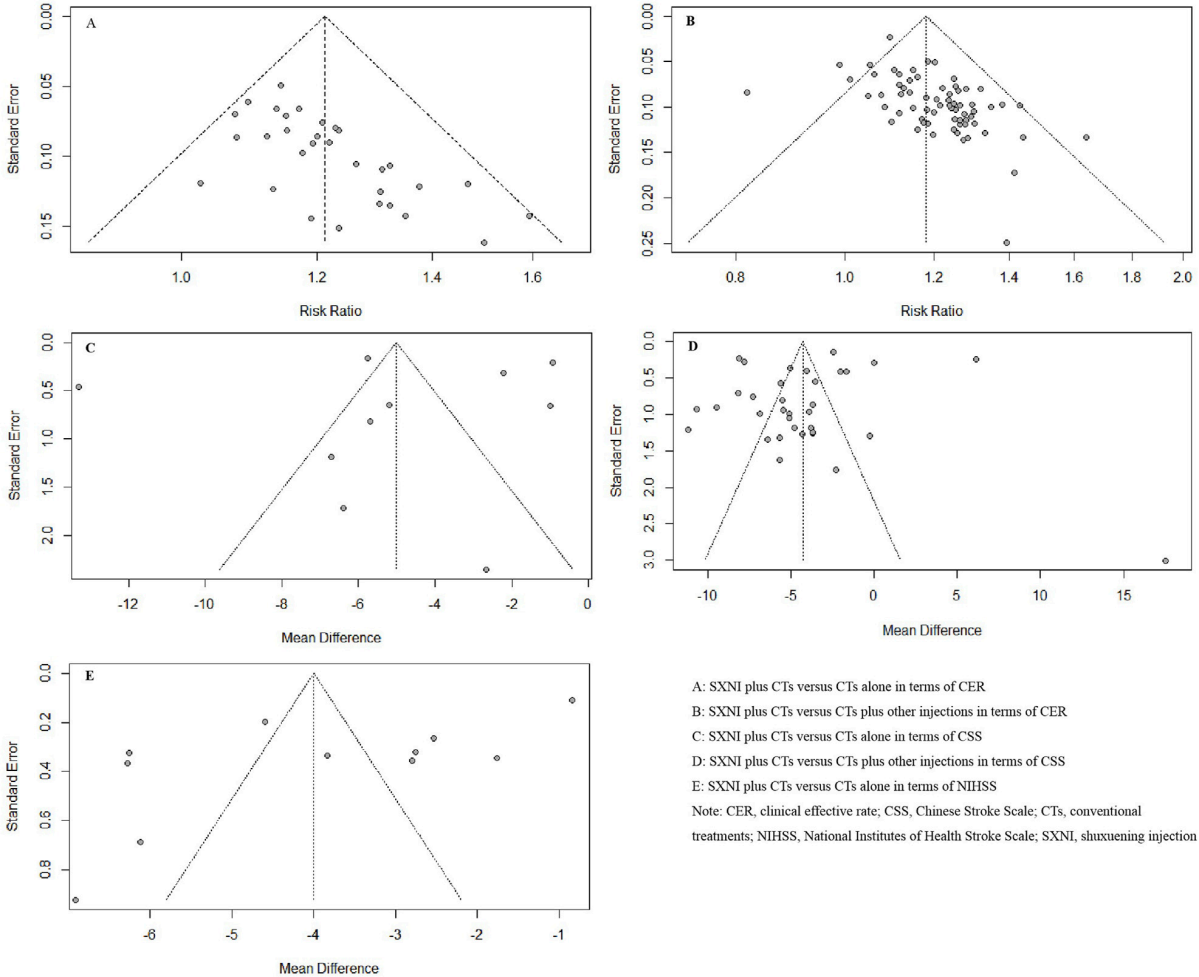
Funnel plots based on the CER, NIHSS, and CSS scores. (Note. CER, clinical effective rate; CSS, Chinese Stroke Scale; CTs, conventional treatments; NIHSS, National Institutes of Health Stroke Scale; SXNI, shuxuening injection).

Thirty-two studies with 3,056 participants reported the CER. We used a fixed effects model due to the low heterogeneity (*I* ^*2*^ = 0%, *p =* 0.68). The meta-analysis results showed that SXNI plus CTs was superior to CTs alone in terms of the CER (*RR*: 1.21, *95% CI*: 1.17–1.25, *Z* = 11.22, *p* < 0.05) (Figure 3A). Sensitivity analysis was conducted by adjusting the random and fixed effects models (Supplementary Table S1) or excluding each study in turn (Supplementary Figure S1), which demonstrated that the results were robust. Subgroup analysis revealed that the effects of different daily doses and different intervention durations of SXNI on CER were not significantly different between SXNI plus CTs and CTs alone (Supplementary Figures S2, S3). Mixed-effect meta-regression analysis further revealed that the sample size, mean age, total dose of SXNI, publication year, and quality of studies were not the main sources of heterogeneity (Supplementary Table S2). Egger’s test (*t* = 4.80, *p* < 0.05) and funnel plots revealed significant publication bias (Figure 4A).

##### 3.4.1.2 SXNI plus CTs vs. CTs plus other injections

Seventy studies with 7,673 participants reported CER. A random effects model was used because of statistical heterogeneity (*I* ^*2*^ = 25%, *p* < 0.05). The results of the meta-analysis showed that SXNI plus CTs was superior to CTs plus other injections in improving the CER (*RR*: 1.18, *95% CI*: 1.15–1.21, Z = 13.26, *p* < 0.05) (Figure 3B). By adjusting the analysis models (Supplementary Table S1) or removing each study in turn (Supplementary Figure S4), sensitivity analysis indicated that the results were robust. Subgroup analysis revealed that the effects of different daily doses, different intervention durations, and different injections on CER were not significantly different between SXNI plus CTs and CTs plus other injections (Supplementary Figures S5–7). Mixed-effect meta-regression models did not reveal sample size, mean age, total dose of SXNI, publication year, or quality of studies as sources of heterogeneity (Supplementary Table S2). The results of Egger’s test (*t* = 5.25, *p* < 0.05) and funnel plots showed publication bias (Figure 4B).

### 3.5 Secondary outcomes

#### 3.5.1 Neurological function

##### 3.5.1.1 SXNI plus CTs vs. CTs alone

**FIGURE 5:**
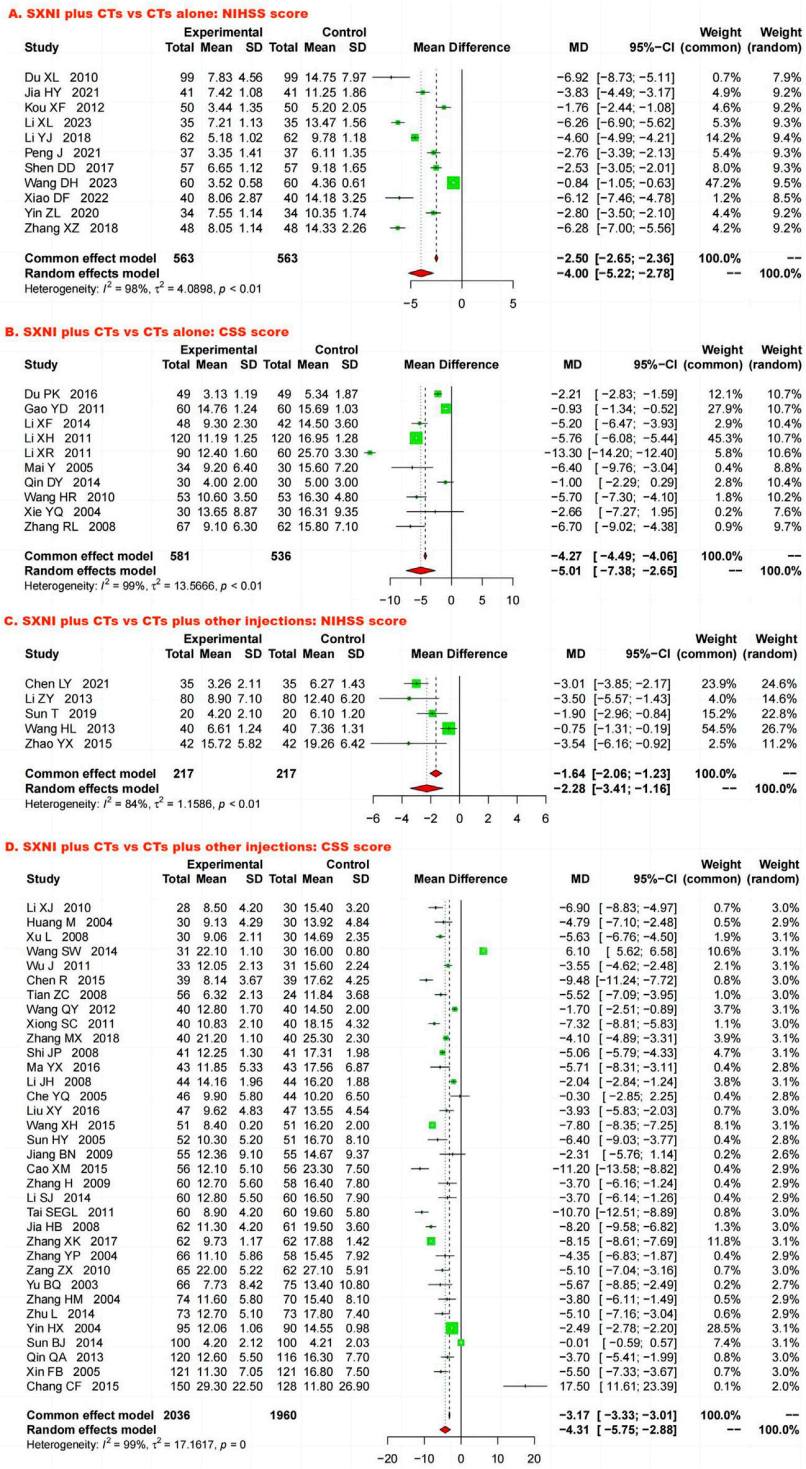
Forest plot and meta-analysis of neurological function. (Note. CSS, Chinese Stroke Scale; CTs, conventional treatments; NIHSS, National Institutes of Health Stroke Scale; SXNI, shuxuening injection).

Eleven studies, including 1,126 participants, assessed neurological function using the NIHSS. We used a random effects model due to the significant heterogeneity (I^2^ = 98%, *p* < 0.05). SXNI plus CTs was superior for decreasing the NIHSS score compared with CTs alone (MD: −4.00, 95% CI: −5.22 to −2.78, Z = −6.42, *p* < 0.05) (Figure 5A). Sensitivity analysis was conducted by adjusting the random and fixed effects models (Supplementary Table S1) or deleting each study in turn (Supplementary Figure S8), which demonstrated that the meta-analysis results were robust. Mixed-effect meta-regression analysis revealed that the sample size, mean age, total dose of SXNI, publication year, and quality of studies did not contribute to the heterogeneity (Supplementary Table S2). Egger’s test (t = −2.99, *p* < 0.05) and funnel plots revealed publication bias (Figure 4E).

Ten studies, including 1,117 participants, evaluated neurological function using CSS. Because of the significant heterogeneity (*I* ^*2*^ = 99%, *p* < 0.05), we used a random effects model to pool the data. SXNI plus CTs was superior to CTs alone for decreasing the CSS (*MD*: −5.01, *95% CI*: −7.38 to −2.65, *Z* = −4.15; *p* < 0.05) (Figure 5B). By adjusting the analysis models (Supplementary Table S1) or removing each study in turn (Supplementary Figure S9), sensitivity analysis indicated that the results were robust. Mixed-effect meta-regression analysis based on sample size, mean age, total dose of SXNI, publication year, and quality of studies did not reveal sources of heterogeneity (Supplementary Table S2). Egger’s test (*t* = −0.36, *p >* 0.05) and funnel plots revealed no publication bias (Figure 4C).

##### 3.5.1.2 SXNI plus CTs vs. CTs plus other injections

Five studies with 434 participants assessed neurological function via the NIHSS. Because of significant heterogeneity (*I* ^*2*^ = 84%, *p* < 0.05), we used a random effects model. The meta-analysis indicated that SXNI plus CTs improved the NIHSS score better than CTs plus other injections did (*MD*: −2.28, *95% CI*: −3.41 to −1.16, *Z* = −3.97, *p* < 0.05) (Figure 5C). Sensitivity analysis was conducted by adjusting the random and fixed effects models (Supplementary Table S1) or deleting each study in turn (Supplementary Figure S10), which demonstrated that the meta-analysis results were robust. No source of heterogeneity was found in mixed-effect meta-regression models based on sample size, mean age, total dose of SXNI, publication year, or quality of studies (Supplementary Table S2).

Thirty-four studies involving 3,996 participants assessed neurological function using the CSS. A random effects model was used to pool the data due to the significant heterogeneity (*I* ^*2*^ = 99%, *p* < 0.05). The results of the meta-analysis showed that SXNI plus CTs decreased the CSS score in AIS patients more than CTs plus other injections did (*MD*: −4.31, *95% CI*: −5.75 to −2.88, *Z* = −5.89, *p* < 0.05) (Figure 5D). By adjusting the analysis models (Supplementary Table S1) or removing each study in turn (Supplementary Figure S11), sensitivity analysis indicated that the results were robust. Subgroup analysis indicated that the effects of different daily doses, different intervention durations, and different injections on decreasing the CSS score were not significantly different between SXNI plus CTs and CTs plus other injections (Supplementary Figures S12–14). Mixed-effect meta-regression models revealed that sample size, mean age, total dose of SXNI, publication year, and quality of studies were not the main sources of heterogeneity (Supplementary Table S2). Egger’s test (*t* = −1.20, *p >* 0.05) and funnel plots revealed no publication bias (Figure 4D).

#### 3.5.2 Activities of daily living

##### 3.5.2.1 SXNI plus CTs vs. CTs alone

**FIGURE 6:**
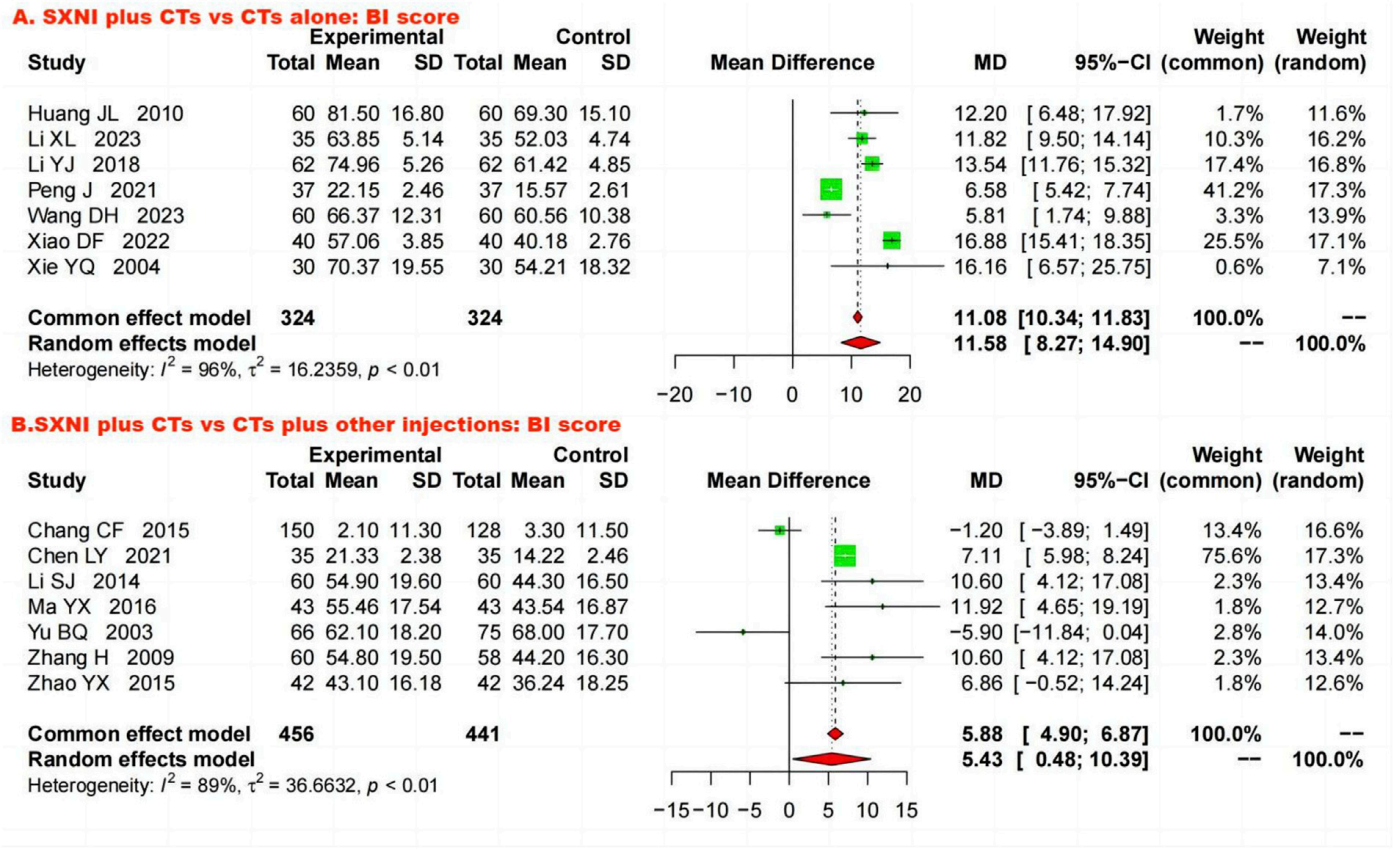
Forest plot and meta-analysis of activities of daily living. (Note. BI, Barthel index; CTs, conventional treatments; SXNI, shuxuening injection).

Seven studies with 648 participants assessed ADLs using the BI. We used a random effects model to pool the data because of the substantial heterogeneity (*I* ^*2*^ = 96%, *p* < 0.05). The meta-analysis showed that SXNI plus CTs was better at improving the BI score than CTs alone (*MD*: 11.58, *95% CI*: 8.27–14.90, *Z* = 6.84, *p* < 0.05) (Figure 6A). By adjusting the analysis models (Supplementary Table S1) or removing each study in turn (Supplementary Figure S15), sensitivity analysis demonstrated the reliability of the results. Mixed-effect meta-regression analysis revealed that sample size, mean age, total dose of SXNI, publication year, and quality of studies were not the sources of heterogeneity (Supplementary Table S2).

##### 3.5.2.2 SXNI plus CTs vs. CTs plus other injections

Seven studies involving 897 participants evaluated ADL by the BI. Due to significant heterogeneity (*I* ^*2*^ = 89%, *p* < 0.05), a random effects model was used. The results showed that SXNI plus CTs was superior to CTs plus other injections for increasing BI scores (*MD*: 5.43, *95% CI*: 0.48–10.39, *Z* = 2.15, *p* < 0.05) (Figure 6B). Sensitivity analysis by adjusting the statistical models showed that the meta-analysis results were robust (Supplementary Table S1). However, the difference in the BI scores between SXNI plus CTs and CTs plus other injections was not significant when five studies were removed one at a time (Chen, 2021; Li, 2014; Ma, 2016; Zhang et al., 2009; Zhao, 2015) (Supplementary Figure S16). Mixed-effect meta-regression did not reveal sample size, mean age, total dose of SXNI, publication year, or quality of studies as sources of heterogeneity (Supplementary Table S1).

### 3.6 AEs

Seventy-one studies reported AEs, and 49 reported no AEs. Twenty-two studies reported AEs related to the SXNI group or non-SXNI group, five of which did not report the number of AEs in the SXNI group, and two did not report the number of AEs in the non-SXNI group. SXNI plus CTs (1.53%, 61/3,994) was similarly safe to CTs alone or CTs plus other injections (1.32%, 50/3,797). The most common AEs related to SXNI were cardiovascular system events, and the five most common symptoms were dizziness, flushing, palpitations, nausea, and headache. The most common AEs related to the non-SXNI group were digestive system events, and the five most common symptoms were palpitations, nausea, dizziness, flushing, and vomiting. Moreover, all these symptoms in both the SXNI group and the non-SXNI group were mild and disappeared after discontinuation of the drug and symptomatic treatment. The symptoms of AEs are detailed in Supplementary Table S3.

### 3.7 Quality of evidence

We used the GRADE tool to systematically evaluate the quality of each outcome. The levels of evidence for CER and BI scores were low in SXNI plus CTs compared with CTs alone for AIS, while the levels of evidence for the NIHSS score and CSS score were very low (Table 3). Additionally, the level of evidence for the CER, NIHSS, CSS, and BI scores were all very low for SXNI plus CTs compared with CTs plus other injections for AIS (Table 3). The grade was decreased mainly because of the low methodological quality of the included studies, high heterogeneity among the included studies, and potential publication bias.

**TABLE 3:** Summary of findings according to the Grading Recommendations Assessment, Development, and Evaluations (GRADE) tool. (Note. CI, confidence interval; CTs, conventional treatments; MD, mean difference; RR, risk ratio; SXNI, shuxuening injection).

Certainty assessment							No of patients		Effect		Certainty		Importance
No of studies	Study design	Risk of bias	Inconsistency	Indirectness	Imprecision	Other considerations	SXNI plus CTs	CTs alone	Relative (95% CI)	Absolute (95% CI)			
A. SXNI plus CTs versus CTs alone													
Clinical Effective Rate													
32	randomised trials	seriousa	not serious	not serious	not serious	publication bias strongly suspectedd	1397/1550 (90.1%)	1119/1506 (74.3%)	RR 1.21 (1.17 to 1.25)	156 more per 1,000(from 126 more to 186 more)	⊕⊕○○○ Low		CRITICAL
Chinese Stroke Scale													
10	randomised trials	very seriousb	very seriousc	not serious	not serious	none	581	536	-	MD 5.01 lower (7.38 lower to 2.65 lower)		⊕○○○ Very low	IMPORTANT
National Institute of Health Stroke Scale													
11	randomised trials	not serious	very seriousc	not serious	not serious	publication bias strongly suspectedd	563	563	-	MD 4 lower (5.22 lower to 2.78 lower)		⊕○○○ Very low	IMPORTANT
Barthel Index													
7	randomised trials	not serious	very seriousc	not serious	not serious	none	324	324	-	MD 11.58 higher (8.27 higher to 14.9 higher)		⊕⊕○○ Low	IMPORTANT
B. SXNI plus CTs versus CTs plus other injections													
Clinical Efficiency Rate													
70	randomised trials	very seriousb	not serious	not serious	not serious	publication bias strongly suspectedd	3528/3910 (90.2%)	2849/3763 (75.7%)	RR 1.18 (1.15 to 1.21)	136 more per 1,000 (from 114 more to 159 more)		⊕○○○ Very low	CRITICAL
Chinese Stroke Scale													
34	randomised trials	very seriousb	very seriousc	not serious	not serious	none	2036	1960	-	MD 4.31 lower (5.75 lower to 2.88 lower)		⊕○○○ Very low	IMPORTANT
National Institutes of Health Stroke Scale													
5	randomised trials	very seriousb	very seriousc	not serious	not serious	none	217	217	-	MD 2.28 lower (3.41 lower to 1.16 lower)		⊕○○○ Very low	IMPORTANT
Barthel Index													
7	randomised trials	very seriousb	very seriousc	not serious	not serious	publication bias strongly suspectedd	456	441	-	MD 5.43 higher (0.48 higher to 10.39 higher)		⊕○○○ Very low	IMPORTANT

Note: CI, confidence interval; CTs, conventional treatments; MD, mean difference; RR, risk ratio; SXNI, shuxuening injection.

Explanations:

^a^ . 50% - 75% of the studies were those with a higher risk of overall bias.

^b^ . More than 75% of the studies were those with a higher risk of overall bias.

^c^ . Heterogeneity among the studies was substantial.

^d^ . There was a risk of publication bias.

## 4 Discussion

### 4.1 Summary of the evidence

A total of 116 studies involving 12,401 participants were included in this review to assess the efficacy and safety of SXNI as an add-on therapy for patients with AIS. The meta-analysis results showed that SXNI plus CTs was superior to CTs alone or CTs plus other injections in improving patients’ CER, NIHSS, CSS, and BI scores, suggesting that SXNI combined with CTs can significantly improve clinical efficacy, reduce neurological deficits, and promote the recovery of ADL in patients with AIS. Subgroup analysis and mixed-effect meta-regression analysis indicated that duration of intervention, daily dose of SXNI, total dose of SXNI, sample size, mean age, different injections used in the control group, quality of studies, and publication year were not sources of heterogeneity. Except for the BI score between SXNI plus CTs and CTs plus other injections, the sensitivity analysis of the remaining indicators demonstrated that the meta-analysis results were robust. Sensitivity analysis revealed that the difference in BI scores between SXNI plus CTs and CTs plus other injections was not significant when five studies were removed one at a time (Chen, 2021; Li, 2014; Ma, 2016; Zhang et al., 2009; Zhao, 2015). In terms of safety, based on the results of this study, we cautiously believe that SXNI is relatively safe and reliable. Publication bias was found in the CER and NIHSS scores. Only 15 (12.9%) studies were judged to be of moderate to high quality. The levels of evidence for the CER, NIHSS, CSS, and BI scores were very low to low quality. Therefore, the results of this study should be considered with caution.

According to traditional Chinese medicine theory, the main pathological changes in AIS are qi deficiency and blood stasis. Qi is regarded as the main material basement that constitutes the human body and maintains life activities and an energy which manifests simultaneously on the physical and emotional-mental-spiritual level, and it has functions of promoting, warming, defense, transformative action and containment) (Jiang et al., 2023; Tian et al., 2024). Therefore, clinical treatment of this disease is mainly carried out by supplementing qi, promoting blood circulation, and dredging meridians and collaterals. SXNI is an extract of *G. biloba* leaves that promotes blood circulation, eliminates blood stasis, unblocks meridians, tonifies qi, and strengthens the brain (Ding, 2018; Zhang, 2019). From the perspective of Western medicine, SXNI can improve blood rheology, inhibit platelet aggregation, and reduce blood viscosity (Zhang et al., 2005). Ginkgolides can inhibit glutamate receptor-gated calcium channels, significantly reduce Ca^2+^ content in brain cells, prevent intracellular Ca^2+^ overload, and reduce cascade reaction-induced brain cell necrosis (Chandrasekaran et al., 2001). SXNI can improve the energy metabolism and nutrition of hypoxic brain cells via its antioxidant properties (Smith et al., 2002; Nash and Shah, 2015) and reduce the apoptosis of neuronal cells in brain tissue through the inhibition of multiple inflammatory responses and the modulation of oxidative stress levels (Dong et al., 2023). Moreover, SXNI can increase superoxide dismutase activity against oxidative damage caused by ischemia to protect muscle cells (Gao et al., 2007; Koyama et al., 2013; Powers and Lennon, 1999), and Ginkgolide B also can promote muscle regeneration by reviving osteocalcin–GPRC6A signalling (Wang et al., 2023). This may be the potential mechanism by which SXNI can reduce neurological deficits and improve limb function in AIS patients.

A centralized hospital monitoring study of the safety of SXNI injection in 9,735 patients showed that SXNI had a low incidence of AEs and good safety (Qu et al., 2023), which is consistent with our results. However, with the expanding application scope and increasing use frequency of SXNI in clinical practice, it is often abused. Reports of AEs related to SXNI were not uncommon. More than 80% of patients experienced AEs within 2 hours after administration (Chen, 2018; Gong and Shang, 2023), which prompted us to focus on monitoring the patient’s condition during this time period. The occurrence of AEs to SXNI may be related to many factors, such as patient age, type of solvent, dosage, over-the-counter use, and coadministration (Wu, 2019). Therefore, we should pay attention to selecting the appropriate drug solvent, strictly follow the instructions for use, and try to avoid using other injections in combination.

In this review, 12.9% of the included studies were defined as moderate to high quality. Correct randomization and allocation concealment are important conditions for ensuring the minimization of selection bias; however, only 15 (12.9%) studies reported correct randomization methods, and none of the studies reported detailed allocation concealment, blinding, or implementation. Of all studies, 2.6% did not conduct intention-to-treat analysis and did not specify the reason for case dropout, 7.8% may not have completely reported outcomes, and none was registered in advance. Although all outcomes except CER showed significant heterogeneity, our subgroup analysis and mixed-effect regression analysis did not reveal the source of heterogeneity. Sensitivity analysis revealed differences in BI scores between SXNI plus CTs and CTs plus other injections when five studies (Chen, 2021; Li, 2014; Ma, 2016; Zhang et al., 2009; Zhao, 2015) were excluded one by one. The different manufacturers of SXNI, large sample size spans, different age-inclusion criteria, and populations in the different regions of these five studies may be potential sources of heterogeneity. The overall level of evidence for each outcome ranged from very low to low quality. The reasons for this were mainly the low methodological quality of the included studies, high heterogeneity among the included studies, and potential publication bias. Consequently, future RCTs with large sample sizes from multiple centers should be rigorously conducted in accordance with the consolidated standards of reporting trials (CONSORT) and should be registered in advance on the registration platform (Schulz et al., 2010).

### 4.2 Compared with previous studies

Our results suggested that SXNI in the treatment of AIS could not only increase CER and improve neurological function and ADL but also had good safety, which was consistent with the results of five systematic reviews previously published in 2011, 2012, and 2016 (Yan, et al., 2011; Xi et al., 2012; Zheng et al., 2012; Cai and Jiang, 2012; Tan, et al., 2016). We included patients who had AIS within 14 days; however, Xi et al. and Zheng et al. included patients whose stroke occurred within 72 h and 7 days, respectively. According to the use requirements of SXNI, we included only adult stroke patients, but previous studies did not limit the age of AIS patients, which may have affected the scope of its application. Compared with previous meta-analyses (Yan, et al., 2011; Xi et al., 2012; Zheng et al., 2012; Cai and Jiang, 2012; Tan, et al., 2016), our study used more comprehensive outcomes (including CER, neurological function, ADL, and AEs) to evaluate the efficacy and safety of SXNI as an add-on therapy for AIS. However, Yan et al. included only 14 RCTs and did not report neurological deficit scores for outcome measures. Cai et al. only observed the CER of SXNI in AIS patients based on 22 RCTs. Tan et al. did not evaluate the effect of SXNI on the ability of patients with AIS to perform ADL. Moreover, the studies included in the previous meta-analyses (Yan, et al., 2011; Xi et al., 2012; Zheng et al., 2012; Cai and Jiang, 2012; Tan, et al., 2016) were all published before 2016, and many RCTs focusing on SXNI for AIS have been reported since 2016; therefore, our literature search deadline was 2023 to update the evidence.

### 4.3 Implications for practice and research

Our findings supported the clinical use of SXNI as an add-on treatment for AIS patients with a low level of evidence. Moreover, because no long-term follow-up studies have been performed to assess the effect of SXNI on the long-term prognosis of AIS patients, our study can only confirm that an intervention duration of 14–30 days with SXNI can effectively improve the neurological function and ADL of AIS patients. The usual daily dose of SXNI was 20 mL, and the most frequent treatment duration was 14 days. The five most common symptoms related to AEs were dizziness, flushing, palpitations, nausea, and headache, which is consistent with previous studies (Qu et al., 2023; Gong and Shang, 2023). Cardiovascular system events were the most common AEs of SXNI for AIS patients. Therefore, in clinical practice, SXNI should be used in strict accordance with the instructions, and patients should be monitored within 2 h after SXNI administration.

In this review, most (87.1%) included studies were judged as low quality. The lack of a detailed description of allocation concealment and blinding methods is a common problem in most studies. In all, 7.8% of the studies may have had a selective reporting bias. The included studies were all single-center RCTs conducted in China, which may mask the efficacy of SXNI in stroke patients of different ethnicities. Most of the studies were conducted in cities, and there is a lack of studies on SXNI for AIS in rural or primary medical institutions, while AIS patients in rural and primary medical institutions are in urgent need of effective and convenient drugs or technologies. SXNI has the advantages of being easy to use and safe. If SXNI is indeed effective in improving the prognosis of AIS patients, it will compensate for the deficiency caused by limited access to IVT and/or EVT. Large-sample, multicenter, placebo-controlled RCTs, especially in rural or primary medical institutions, should be conducted in the future. Moreover, all RCTs should be registered in an internationally recognized clinical trial registration platform and reported in accordance with the CONSORT guidelines.

### 4.4 Limitations and advantages

Some limitations of this study should be noted. First, all included trials were conducted in China and published in Chinese, which may have led to selection bias and publication bias and reduced the generalizability of the results to the applicable population. Second, most of the included studies were of low quality and had significant heterogeneity, which led to a low level of evidence.

There are also some advantages in our study. First, we carried out an extensive literature search, including seven databases and two clinical trial registration platforms, to obtain as many relevant studies as possible. Second, two reviewers independently performed literature screening, data extraction, and quality evaluation and cross-checked to ensure the accuracy of the data. Third, we conducted a sensitivity analysis, subgroup analysis, and mixed-effects meta-regression analysis to test the robustness of the meta-analysis results. We also assessed the level of evidence for each outcome based on the GRADE tool.

## 5 Conclusion

SXNI, as an add-on therapy, maybe safe and significantly improved the clinical efficacy, neurological function, and ADL of patients with AIS. However, due to the low quality of the included studies and the very low to low level of evidence in the meta-analysis results, more standardized, large sample, multicenter, and long follow-up RCTs are needed to confirm the efficacy and safety of SXNI for AIS.

## Data Availability

The original contributions presented in the study are included in the article/Supplementary Material, further inquiries can be directed to the corresponding authors.

## Abbreviations

ADL, activities of daily living; AEs, adverse events; AIS, acute ischemic stroke; BI, Barthel index; CBM, Chinese biological medicine database; CI, confidence interval; CER, clinical effective rate; CNKI, China national knowledge infrastructure; CONSORT, consolidated standards of reporting trials; CSS, Chinese Stroke Scale; CT, computed tomography; CTs, conventional treatments; EVT, endovascular thrombectomy; GBE, Ginkgo biloba extract; GRADE, Grading of Recommendation, Assessment, Development, and Evaluation; IVT, intravenous thrombolysis; MRI, magnetic resonance imaging; CND, clinical neurological deficit; NIHSS, National Institutes of Health Stroke Scale; RCTs, randomized controlled trials; ROB, risk of bias; RR, risk ratio; SMD, standardized mean difference; SXNI, shuxuening injection; PAF, platelet-activating factor; PRISMA, preferred reporting items for systematic reviews and meta-analysis; VIP, Chinese scientific journal database; WMD, weighted mean difference.
