# Supplementary material for: Shuxuening injection for treating acute ischemic stroke: a PRISMA-compliant systematic review and meta-analysis of randomized controlled trials
Source: Front Pharmacol. 2024 Oct 22;15:1407669. doi: 10.3389/fphar.2024.1407669 (PMC11538198; doi:10.3389/fphar.2024.1407669)
Supplement: Supplementary file 4 [file DataSheet1.docx]

[**Supplementary Material**](#_Toc9491)

[sTable 1 Results of sensitivity analysis after adjusting the statistical models. 1](#_Toc10076)

[sTable 2 Details of mixed-effect meta-regression analysis of the main outcomes](#_Toc2865) 3

[sTable 3 Details of the symptoms of adverse events. 9](#_Toc29140)

[sFigure 1. The results of sensitivity analysis after removing studies one by one (SXNI plus CTs vs. CTs alone: CER). 12](#_Toc20721)

[sFigure 2. The results of subgroup analysis based on daily doses of SXNI (SXNI plus CTs vs. CTs alone: CER).](#_Toc24123) 13

[sFigure 3. The results of subgroup analysis based on intervention duration of SXNI (SXNI plus CTs vs. CTs alone: CER). 14](#_Toc19308)

[sFigure 4. The results of sensitivity analysis after removing studies one by one (SXNI plus CTs vs. CTs plus other injections: CER). 15](#_Toc17886)

[sFigure 5. The results of subgroup analysis based on daily doses of SXNI (SXNI plus CTs vs. CTs plus other injections: CER). 16](#_Toc19690)

[sFigure 6. The results of subgroup analysis based on intervention duration of SXNI (SXNI plus CTs vs. CTs plus other injections: CER). 17](#_Toc24399)

[sFigure 7. The results of subgroup analysis based on different injections in the control group (SXNI plus CTs vs. CTs plus other injections: CER). 18](#_Toc20858)

[sFigure 8. The results of sensitivity analysis after removing studies one by one (SXNI plus CTs vs. CTs alone: NIHSS). 19](#_Toc24346)

[sFigure 9. The results of sensitivity analysis after removing studies one by one (SXNI plus CTs vs. CTs alone: CCS). 19](#_Toc10566)

[sFigure 10. The results of sensitivity analysis after removing studies one by one (SXNI plus CTs vs. CTs plus other injection: NIHSS). 19](#_Toc21323)

[sFigure 11. The results of sensitivity analysis after removing studies one by one (SXNI plus CTs vs. CTs plus other injection: CSS). 20](#_Toc22158)

[sFigure 12. The results of subgroup analysis based on daily doses of SXNI (SXNI plus CTs vs. CTs plus other injections: CSS). 21](#_Toc14336)

[sFigure 13. The results of subgroup analysis based on intervention duration of SXNI (SXNI plus CTs vs. CTs plus other injections:CSS). 22](#_Toc17515)

[sFigure 14. The results of subgroup analysis based on different injections in the control group (SXNI plus CTs vs. CTs plus other injections: CSS). 23](#_Toc30100)

[sFigure 15. The results of sensitivity analysis after removing studies one by one (SXNI plus CTs vs. CTs alone: BI). 24](#_Toc25807)

[sFigure 16. The results of sensitivity analysis after removing studies one by one (SXNI plus CTs vs. CTs plus other injection: BI). 24](#_Toc15659)

[sAppendix 1. Search Strategy. 25](#_Toc30556)

[sAppendix 2. The full citation details of included and excluded studies. 29](#_Toc7853)

# **sTable 1 Results of sensitivity analysis after adjusting the statistical models**

| **Study type** | **Studies, no.** | **Participants, no** | | **Study heterogeneity** | | | | **Analysis model** | **RR or MD**  **(95% CI)** | ***p* value** |
| --- | --- | --- | --- | --- | --- | --- | --- | --- | --- | --- |
|  |  | **T** | **C** | **tau^2^** | **df** | **I^2^, %** | ***p* value** |  |  |  |
| **Clinical effective rate (CER)** | | | | | | | | | | |
| SXNI plus CTs vs CTs alone | 32 | 1550 | 1506 | 0 | 31 | 0.0 | 0.6808 | random | 1.18 (1.14, 1.22) | < 0.0001 |
|  |  |  |  |  |  |  |  | fixed | 1.21 (1.17, 1.25) | < 0.0001 |
| SXNI plus CTs vs CTs plus other injections | 70 | 3910 | 3763 | 0.0029 | 69 | 25.1 | 0.0331 | random | 1.18 (1.15, 1.21) | < 0.0001 |
|  |  |  |  |  |  |  |  | fixed | 1.19 (1.17, 1.22) | < 0.0001 |
| **National Institutes of Health Stroke Scale (NIHSS) score** | | | | | | | | | | |
| SXNI plus CTs vs CTs alone | 11 | 563 | 563 | 4.0898 | 10 | 98.5 | < 0.0001 | random | -4.00 (-5.22, -2.78) | < 0.0001 |
|  |  |  |  |  |  |  |  | fixed | -2.50 (-2.65, -2.36) | < 0.0001 |
| SXNI plus CTs vs CTs plus other injections | 5 | 217 | 217 | 1.1586 | 4 | 84.1 | < 0.0001 | random | -2.28 (-3.41, -1.16) | < 0.0001 |
|  |  |  |  |  |  |  |  | fixed | -1.64 (-2.06, -1.23) | < 0.0001 |
| **Chinese Stroke Scale (CSS) score** | | | | | | | | | | |
| SXNI plus CTs vs CTs alone | 10 | 581 | 536 | 13.566 | 9 | 98.9 | < 0.0001 | random | -5.01 (-7.38, -2.65) | < 0.0001 |
|  |  |  |  |  |  |  |  | fixed | -4.27 (-4.49, -4.06) | < 0.0001 |
| SXNI plus CTs vs CTs plus other injections | 34 | 2036 | 1960 | 17.1617 | 33 | 98.8 | < 0.0001 | random | -4.31 (-5.75, -2.88) | < 0.0001 |
|  |  |  |  |  |  |  |  | fixed | -3.17 (-3.33, -3.01) | < 0.0001 |
| **Barthel Index (BI) score** | | | | | | | | | | |
| SXNI plus CTs vs CTs alone | 7 | 324 | 324 | 16.2359 | 6 | 95.5 | < 0.0001 | random | 11.58 (8.27, 14.90) | < 0.0001 |
|  |  |  |  |  |  |  |  | fixed | 11.08 (10.34, 11.83) | < 0.0001 |
| SXNI plus CTs vs CTs plus other injections | 7 | 456 | 441 | 36.6632 | 6 | 88.7 | < 0.0001 | random | 5.43 (0.48, 10.39) | < 0.0001 |
|  |  |  |  |  |  |  |  | fixed | 5.88 (4.90, 6.87) | < 0.0001 |

Note: CI, confidence interval; CTs, conventional treatments; MD, mean difference; RR, risk ratio; SXNI, shuxuening injection.

# **sTable 2 Details of mixed-effect meta-regression analysis of the main outcomes**

**SXNI plus CTs vs CTs alone: CER**

Mixed-Effects Model (k = 32; tau-squared estimator: REML)

tau-squared (estimated amount of residual heterogeneity): 0 (SE = 0.0021)

tau (square root of estimated tau-squared value): 0

I-squared (residual heterogeneity / unaccounted variability): 0.00%

H-squared (unaccounted variability / sampling variability): 1.00

R-squared (amount of heterogeneity accounted for): 0.00%

Test for Residual Heterogeneity: QE(df = 25) = 23.7155, p = 0.5359

Test of Moderators (coefficients 2:7): QM(df = 6) = 3.1124, p = 0.7946

Model Results:

| **covariates** | **estimate** | **se** | ***z*-value** | ***p*-value** | **95%CI** |
| --- | --- | --- | --- | --- | --- |
| Intrcpt | 1.404 | 9.271 | 0.151 | 0.880 | -16.77 to 19.58 |
| Total dose (more than 280 ml) | -0.030 | 0.049 | -0.614 | 0.539 | -0.13 to 0.07 |
| Age (mix) | -0.030 | 0.054 | -0.546 | 0.585 | -0.14 to 0.08 |
| Age (more than 60 years old) | -0.054 | 0.046 | -1.165 | 0.244 | -0.14 to 0.04 |
| Sample (more than 107） | -0.024 | 0.039 | -0.621 | 0.535 | -0.10 to 0.05 |
| Publication | -0.001 | 0.005 | -0.126 | 0.900 | -0.01 to 0.01 |
| Quality (low) | -0.029 | 0.045 | -0.643 | 0.520 | -0.12 to 0.06 |

**SXNI plus CTs vs CTs plus other injections: CER**

Mixed-Effects Model (k = 70; tau-squared estimator: REML)

tau-squared (estimated amount of residual heterogeneity): 0.0021 (SE = 0.0016)

tau (square root of estimated tau-squared value): 0.0453

I-squared (residual heterogeneity / unaccounted variability): 21.46%

H-squared (unaccounted variability / sampling variability): 1.27

R-squared (amount of heterogeneity accounted for): 29.30%

Test for Residual Heterogeneity: QE(df = 63) = 74.8510, p = 0.145

Test of Moderators (coefficients 2:7): QM(df = 6) = 8.8543, p = 0.1819

Model Results:

| covariates | estimate | se | *z*-value | *p*-value | 95%CI |
| --- | --- | --- | --- | --- | --- |
| Intrcpt | -10.468 | 6.093 | -1.718 | 0.086 | -22.41 to 1.47 |
| Total dose (more than 280 ml) | -0.017 | 0.030 | -0.552 | 0.581 | -0.08 to 0.04 |
| Age (mix) | 0.033 | 0.038 | 0.868 | 0.386 | -0.04 to 0.11 |
| Age (more than 60 years old) | -0.008 | 0.037 | -0.219 | 0.827 | -0.08 to 0.06 |
| Sample (more than 107) | -0.038 | 0.025 | -1.518 | 0.129 | -0.09 to 0.01 |
| Publication | 0.005 | 0.003 | 1.756 | 0.079 | -0.00 to 0.01 |
| Quality (low) | -0.027 | 0.067 | -0.409 | 0.683 | -0.16 to 0.10 |

**SXNI plus CTs vs CTs alone: NIHSS**

Mixed-Effects Model (k = 11; tau-squared estimator: REML)

tau-squared (estimated amount of residual heterogeneity): 5.5183 (SE = 3.2925)

tau (square root of estimated tau-squared value): 2.3491

I-squared (residual heterogeneity / unaccounted variability): 98.13%

H-squared (unaccounted variability / sampling variability): 53.38

R-squared (amount of heterogeneity accounted for): 0.00%

Test for Residual Heterogeneity: QE(df = 6) = 406.5272, p < 0.0001

Test of Moderators (coefficients 2:5): QM(df = 4) = 1.5876, p = 0.8110

Model Results:

| covariates | estimate | se | *z*-value | *p*-value | 95%CI |
| --- | --- | --- | --- | --- | --- |
| Intrcpt | 289.455 | 616.507 | 0.470 | 0.639 | -918.86 to 1497.79 |
| Age (more than 60 years old) | -1.850 | 1.767 | -1.047 | 0.295 | -5.31 to 1.61 |
| Sample (more than 107) | -0.435 | 2.001 | -0.217 | 0.828 | -4.36 to 3.49 |
| Publication | -0.145 | 0.305 | -0.474 | 0.635 | -0.74 to 0.45 |
| Quality (low) | -1.465 | 2.284 | -0.642 | 0.521 | -5.94 to 3.01 |

**SXNI plus CTs vs CTs alone: CSS**

Mixed-Effects Model (k = 10; tau-squared estimator: REML)

tau-squared (estimated amount of residual heterogeneity): 20.3972 (SE = 14.7111)

tau (square root of estimated tau-squared value): 4.5163

I-squared (residual heterogeneity / unaccounted variability): 99.50%

H-squared (unaccounted variability / sampling variability): 201.57

R-squared (amount of heterogeneity accounted for): 0.00%

Test for Residual Heterogeneity: QE(df = 4) = 730.6322, p < 0.0001

Test of Moderators (coefficients 2:6): QM(df = 5) = 1.8323, p = 0.8718

Model Results:

| covariates | estimate | se | *z*-value | *p*-value | 95%CI |
| --- | --- | --- | --- | --- | --- |
| Intrcpt | -571.457 | 1086.793 | -0.526 | 0.599 | -2701.53 to 1558.62 |
| Age (mix) | -1.161 | 8.735 | -0.133 | 0.894 | -18.28 to 15.96 |
| Age (more than 60 years old) | -4.103 | 7.190 | -0.571 | 0.568 | -18.19 to 9.99 |
| Sample (more than 107) | -4.610 | 5.487 | -0.840 | 0.401 | -15.36 to 6.14 |
| Publication | 0.282 | 0.543 | 0.519 | 0.604 | -0.78 to 1.35 |
| Quality (low) | 3.572 | 6.828 | 0.523 | 0.601 | -9.81 to 16.95 |

**SXNI plus CTs vs CTs plus other injections: NIHSS**

Mixed-Effects Model (k = 11; tau-squared estimator: REML)

tau-squared (estimated amount of residual heterogeneity): 5.5183 (SE = 3.2925)

tau (square root of estimated tau-squared value): 2.3491

I-squared (residual heterogeneity / unaccounted variability): 98.13%

H-squared (unaccounted variability / sampling variability): 53.38

R-squared (amount of heterogeneity accounted for): 0.00%

Test for Residual Heterogeneity: QE(df = 6) = 406.5272, pl < 0.0001

Test of Moderators (coefficients 2:5): QM(df = 4) = 1.5876, p = 0.8110

Model Results:

| covariates | estimate | se | *z*-value | *p*-value | 95%CI |
| --- | --- | --- | --- | --- | --- |
| Intrcpt | 289.455 | 616.507 | 0.470 | 0.639 | -918.88 to 1497.79 |
| Age (more than 60 years old) | -1.850 | 1.767 | -1.047 | 0.295 | -5.31 to 1.61 |
| Sample (more than 107) | -0.435 | 2.001 | -0.217 | 0.828 | -4.36 to 3.49 |
| Publication | -0.145 | 0.305 | -0.474 | 0.635 | -0.74 to 0.45 |
| Quality (low) | -1.465 | 2.284 | -0.642 | 0.521 | -5.94 to 3.01 |

**SXNI plus CTs vs CTs plus other injections: CSS**

Mixed-Effects Model (k = 34; tau-squared estimator: REML)

tau-squared (estimated amount of residual heterogeneity): 20.0077 (SE = 5.7419)

tau (square root of estimated tau-squared value): 4.4730

I-squared (residual heterogeneity / unaccounted variability): 98.03%

H-squared (unaccounted variability / sampling variability): 50.83

R-squared2 (amount of heterogeneity accounted for): 0.00%

Test for Residual Heterogeneity: QE(df = 27) = 1672.6648, pl < 0.0001

Test of Moderators (coefficients 2:7): QM(df = 6) = 2.8897, pl = 0.8226

Model Results:

| covariates | estimate | se | *z*-value | *p*-value | 95%CI |
| --- | --- | --- | --- | --- | --- |
| Intrcpt | -164.584 | 385.127 | -0.427 | 0.669 | -919.42 to 590.25 |
| Total dose (more than 280 ml) | -1.845 | 2.282 | -0.809 | 0.419 | -6.32 to 2.63 |
| Age (mix) | 3.841 | 2.577 | 1.490 | 0.136 | -1.21 to 8.89 |
| Age (more than 60 years old) | 3.475 | 2.437 | 1.426 | 0.154 | -1.30 to 8.25 |
| Sample (more than 107) | 0.124 | 1.751 | 0.071 | 0.943 | -3.31 to 3.56 |
| Publication | 0.079 | 0.191 | 0.413 | 0.680 | -0.30 to 0.45 |
| Quality (low) | -1.527 | 3.669 | -0.416 | 0.677 | -8.72 to 5.66 |

**SXNI plus CTs vs CTs alone: BI**

Mixed-Effects Model (k = 7; tau-squared estimator: REML)

tau-squared (estimated amount of residual heterogeneity): 34.3503 (SE = 35.2307)

tau (square root of estimated tau^2 value): 5.8609

I-squared (residual heterogeneity / unaccounted variability): 97.89%

H-squared (unaccounted variability / sampling variability): 47.33

R-squared (amount of heterogeneity accounted for): 0.00%

Test for Residual Heterogeneity: QE(df = 2) = 125.9384, p < 0.0001

Test of Moderators (coefficients 2:5): QM(df = 4) = 0.8821, p = 0.9271

Model Results:

| covariates | estimate | se | *z*-value | *p*-value | 95%CI |
| --- | --- | --- | --- | --- | --- |
| Intrcpt | 1911.495 | 3203.829 | 0.597 | 0.551 | -4367.89 to 8190.88 |
| Total dose (more than 280 ml) | -7.438 | 18.273 | -0.407 | 0.684 | -43.25 to 28.38 |
| Age (more than 60 years old) | 5.099 | 16.664 | 0.306 | 0.760 | -27.56 to 37.76 |
| Sample (more than 107) | 1.692 | 13.864 | 0.122 | 0.903 | -25.48 to 28.86 |
| Publication | -0.942 | 1.591 | -0.592 | 0.554 | -4.06 to 2.18 |

**SXNI plus CTs vs CTs plus other injections: BI**

Mixed-Effects Model (k = 7; tau-squared estimator: REML)

tau-squared (estimated amount of residual heterogeneity): 73.6507 (SE = 113.7947)

tau (square root of estimated tau-squared value): 8.5820

I-squared (residual heterogeneity / unaccounted variability): 91.53%

H-squared (unaccounted variability / sampling variability): 11.81

R-squared (amount of heterogeneity accounted for): 0.00%

Test for Residual Heterogeneity: QE(df = 1) = 11.8083, p-val = 0.0006

Test of Moderators (coefficients 2:6): QM(df = 5) = 2.3053, p-val = 0.8055

Model Results:

| covariates | estimate | se | *z*-value | *p*-value | 95%CI |
| --- | --- | --- | --- | --- | --- |
| Intrcpt | -1645.572 | 1936.727 | -0.850 | 0.396 | -5441.49 to 2150.34 |
| Total dose (more than 280 ml) | -4.242 | 13.272 | -0.320 | 0.749 | -30.26 to 21.77 |
| Age (mix) | 19.926 | 17.752 | 1.123 | 0.262 | -14.87 to 54.72 |
| Age (more than 60 years old) | 8.890 | 13.578 | 0.655 | 0.512 | -17.71 to 35.51 |
| Sample (more than 107) | -6.623 | 11.806 | -0.561 | 0.575 | -29.76 to 16.52 |
| Publication | 0.818 | 0.958 | 0.853 | 0.394 | -1.06 to 2.70 |

# **sTable 3 Details of the symptoms of adverse events**

| **Author, year** | **SXNI Group** | **non-SXNI Group** |
| --- | --- | --- |
| Chen ZC 2014 | Spirit and nervous system: dizziness (unclear cases);  Cardiovascular system: palpitation (unclear cases) | no |
| Du XL 2010 | Skin and adnexa: skin pruritus (2 cases) | no |
| Gao YD 2011 | Cardiovascular system: flushing (5 cases) | no |
| He J 2010 | Spirit and nervous system: dizziness (3 cases);  Cardiovascular system: palpitation and flushing (1 case) | no |
| Hua GC 2002 | Digestive system: gastrointestinal discomfort (unclear case) | no |
| Jia HB 2008 | Spirit and nervous system: dizziness (3 cases);  Cardiovascular system: palpitation and flushing (1 case) | no |
| Kou XF 2012 | Cardiovascular system: flushing (3 cases) | no |
| Ling YX 2006 | no | Skin and adnexa: allergic rash (3 cases) |
| Nie XP 2020 | Spirit and nervous system: facial muscle tension (1 case);  Digestive system: nausea (1 case) | Spirit and nervous system: facial muscle tension (3 cases), headache (4 cases);  Digestive system: vomiting (2 cases), nausea (1 case) |
| Shi JP 2008 | Digestive system: stomach discomfort (3 cases) | Digestive system: stomach discomfort (4 cases) |
| Wang DH 2023 | Digestive system: nausea and vomiting (2 cases), stomachache (1 case);  Spirit and nervous system: dizziness (2 cases) | Digestive system: nausea and vomiting (6 cases), stomachache (1 case );  Skin and adnexa: skin pruritus (2 cases);  Spirit and nervous system: dizziness (4 cases) |
| Xiao DF 2022 | Cardiovascular system: flushing (1 cases) | no |
| Xie YQ 2004 | Spirit and nervous system: headache and dizziness (3 cases), transient mental excitement (1 case) | no |
| Yan YX 2010 | Spirit and nervous system: headache (2cases);  Cardiovascular system: flushing (2 cases) | no |
| Yang JH 2010 | Spirit and nervous system: dizziness (1 case);  Cardiovascular system: flushing (1 case);  Skin and adnexa: Allergic rash (1 case) | no |
| Yu BQ 2003 | Spirit and nervous system: headache (unclear cases); Cardiovascular system: slight burning sensation (unclear cases) | no |
| Zhang GJ 2010 | Spirit and nervous system: dizziness (6 cases);  Cardiovascular system: palpitation and flushing (1 case) | no |
| Zhang HX 2006 | Cardiovascular system: slight burning sensation, palpitations or phlebitis (7 cases), cerebral hemorrhage (1 case);  Skin and adnexa: ecchymosis of skin (2 cases) | Cardiovascular system and digestive system: slight burning sensation, nausea, palpitations or phlebitis (9 cases), cerebral hemorrhage (2 cases);  Skin and adnexa: ecchymosis of skin (2 cases) |
| Zhang XK 2017 | Cardiovascular system: flushing (unclear case);  Digestive system: inappetence (unclear case) | Cardiovascular system: flushing (unclear case);  Digestive system: inappetence (unclear case) |
| Zhao YX 2015 | Urinary system: elevated blood urea nitrogen (1 case);  Digestive system: elevated transaminase (1 cases) | Urinary system: elevated blood urea nitrogen (3 cases);  Digestive system: elevated transaminase (2 cases) |
| Zheng YZ 2014 | Digestive system (unclear case);  Urinary system (unclear case) | Digestive system (unclear case); Urinary system (unclear case) |
| Zhu L 2014 | Spirit and nervous system: headache (1 case);  Digestive system: nausea (1 case) | Digestive system: diarrhea (2 cases) |

# **sFigure 1. The results of the sensitivity analysis after removing studies one by one (SXNI plus CTs vs. CTs alone: CER).**


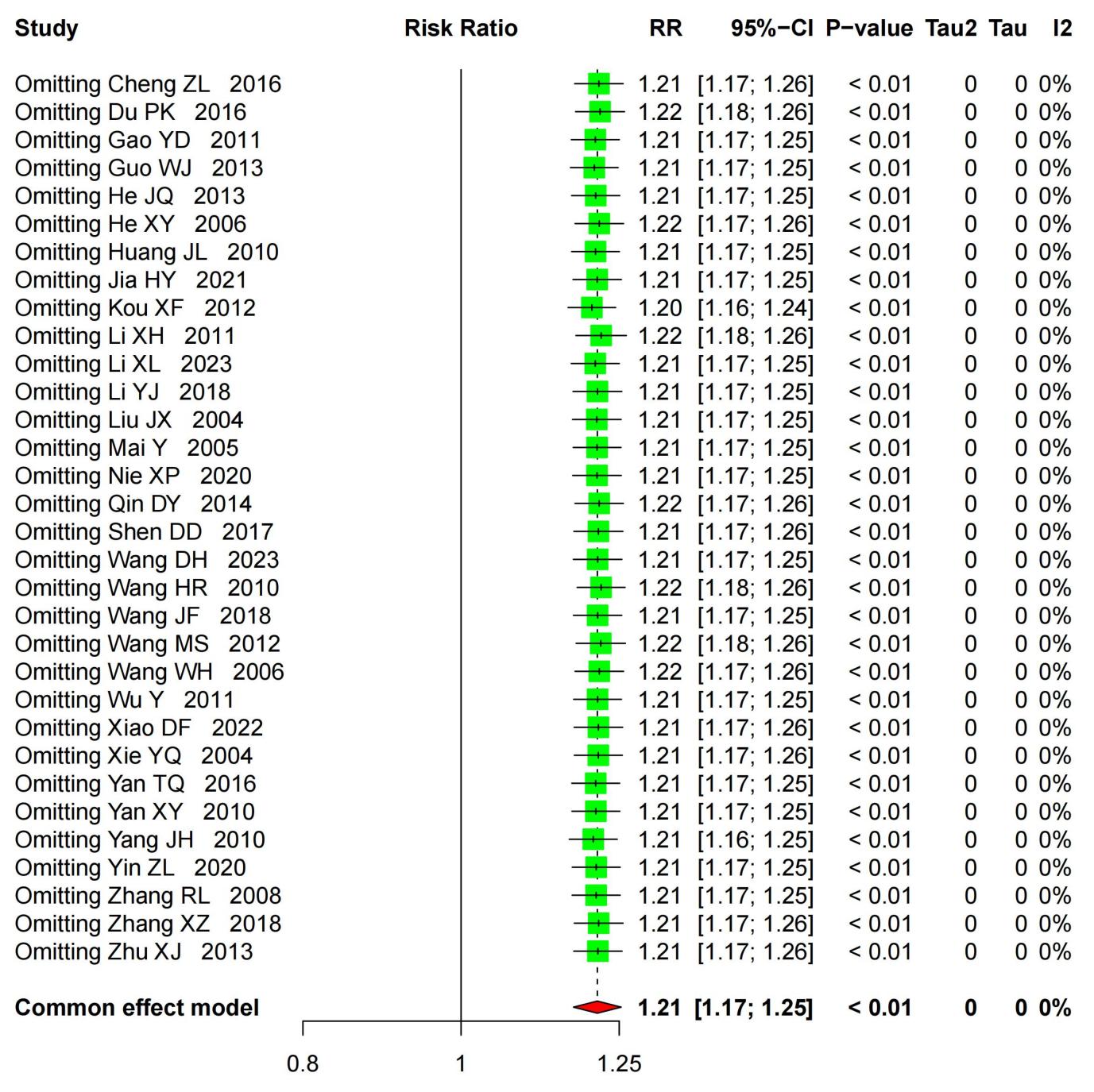


Note: CER, clinical effective rate; CTs, conventional treatments; SXNI, shuxuening injection.

# **sFigure 2. The results of subgroup analysis based on daily doses of SXNI (SXNI plus CTs vs. CTs alone: CER).**


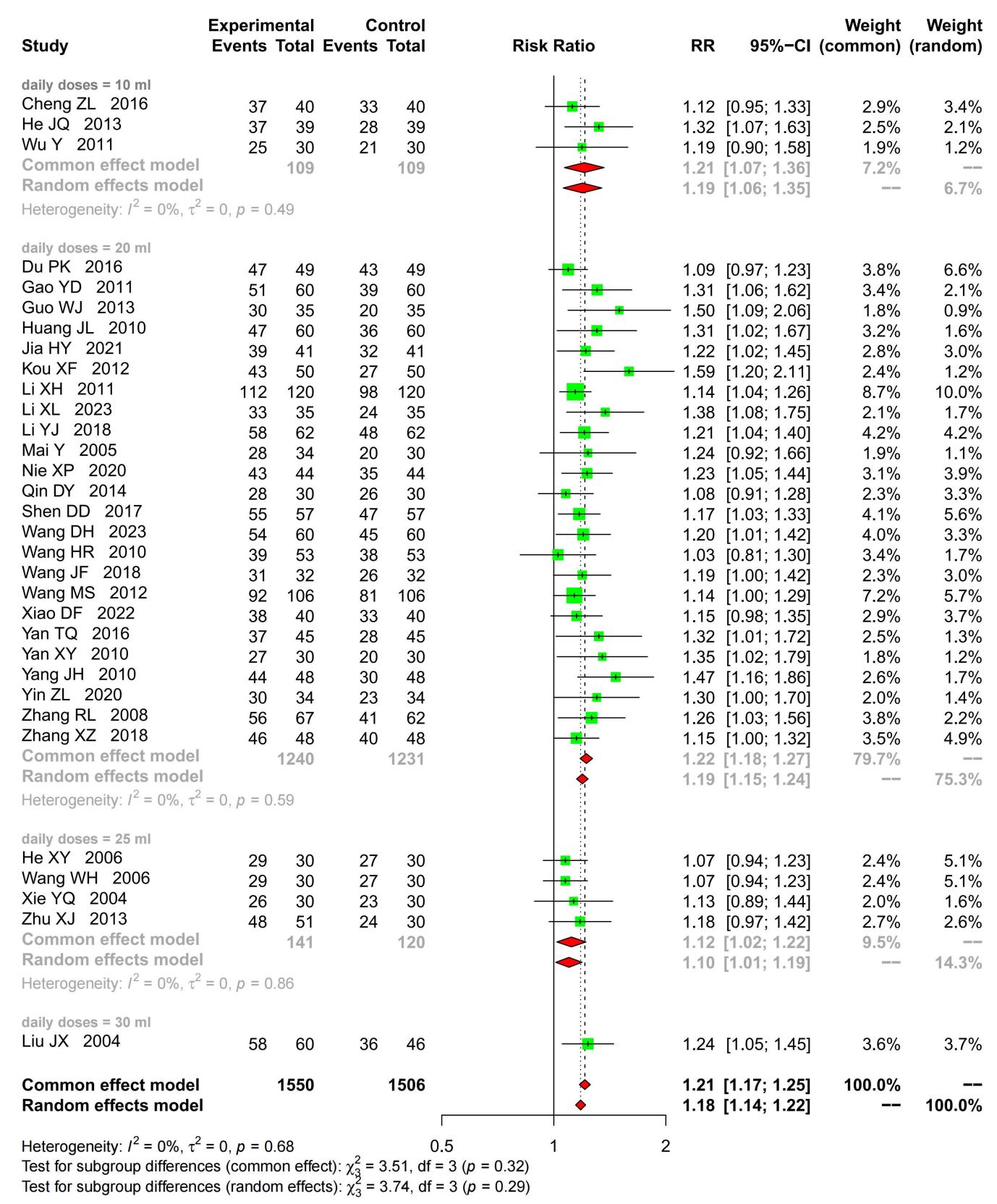


Note: CER, clinical effective rate; CTs, conventional treatments; SXNI, shuxuening injection.

# **sFigure 3. The results of the subgroup analysis based on the duration of SXNI intervention (SXNI plus CTs vs. CTs alone: CER).**


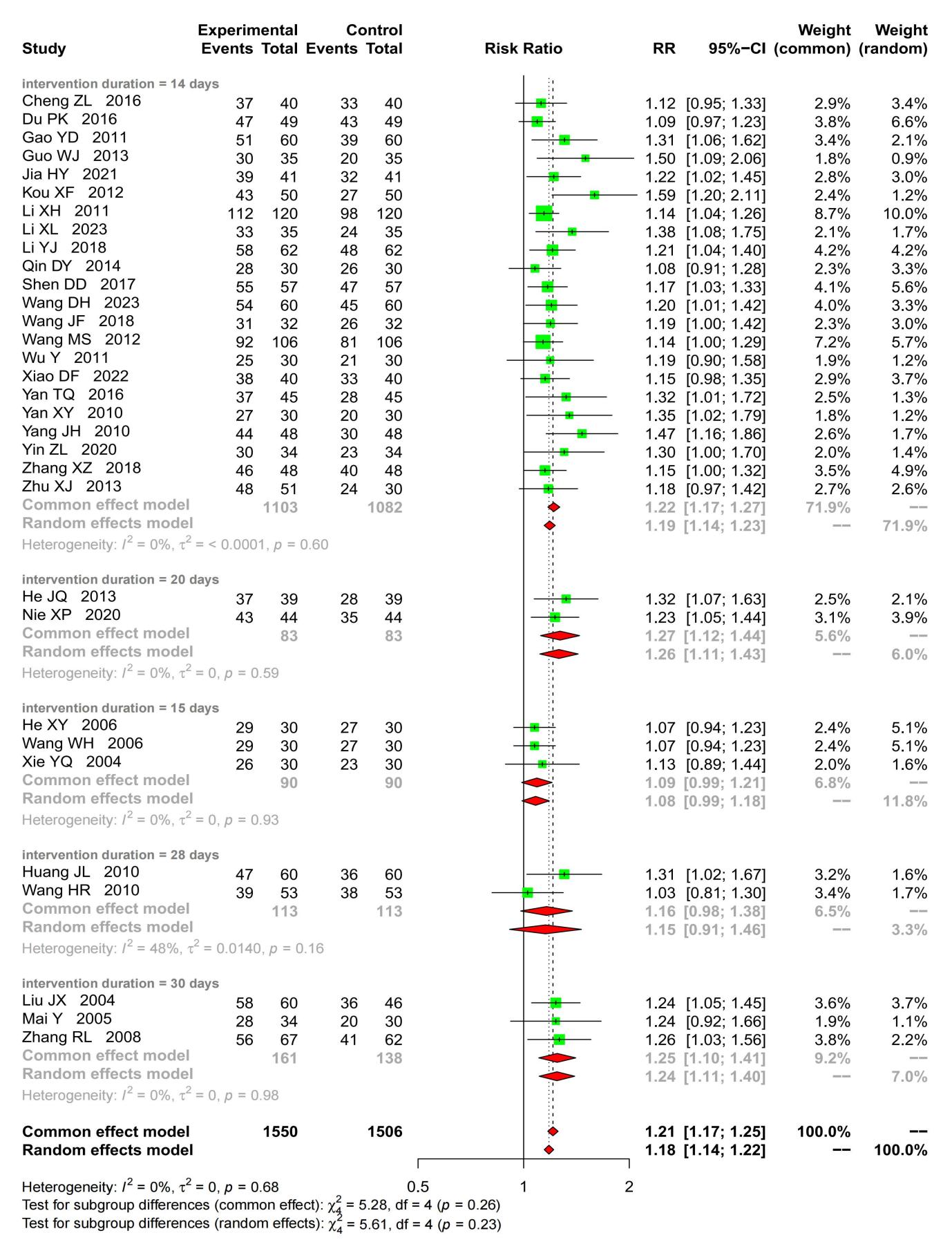


Note: CER, clinical effective rate; CTs, conventional treatments; SXNI, shuxuening injection.

# **sFigure 4. The results of the sensitivity analysis after removing studies one by one (SXNI plus CTs vs. CTs plus other injections: CER).**


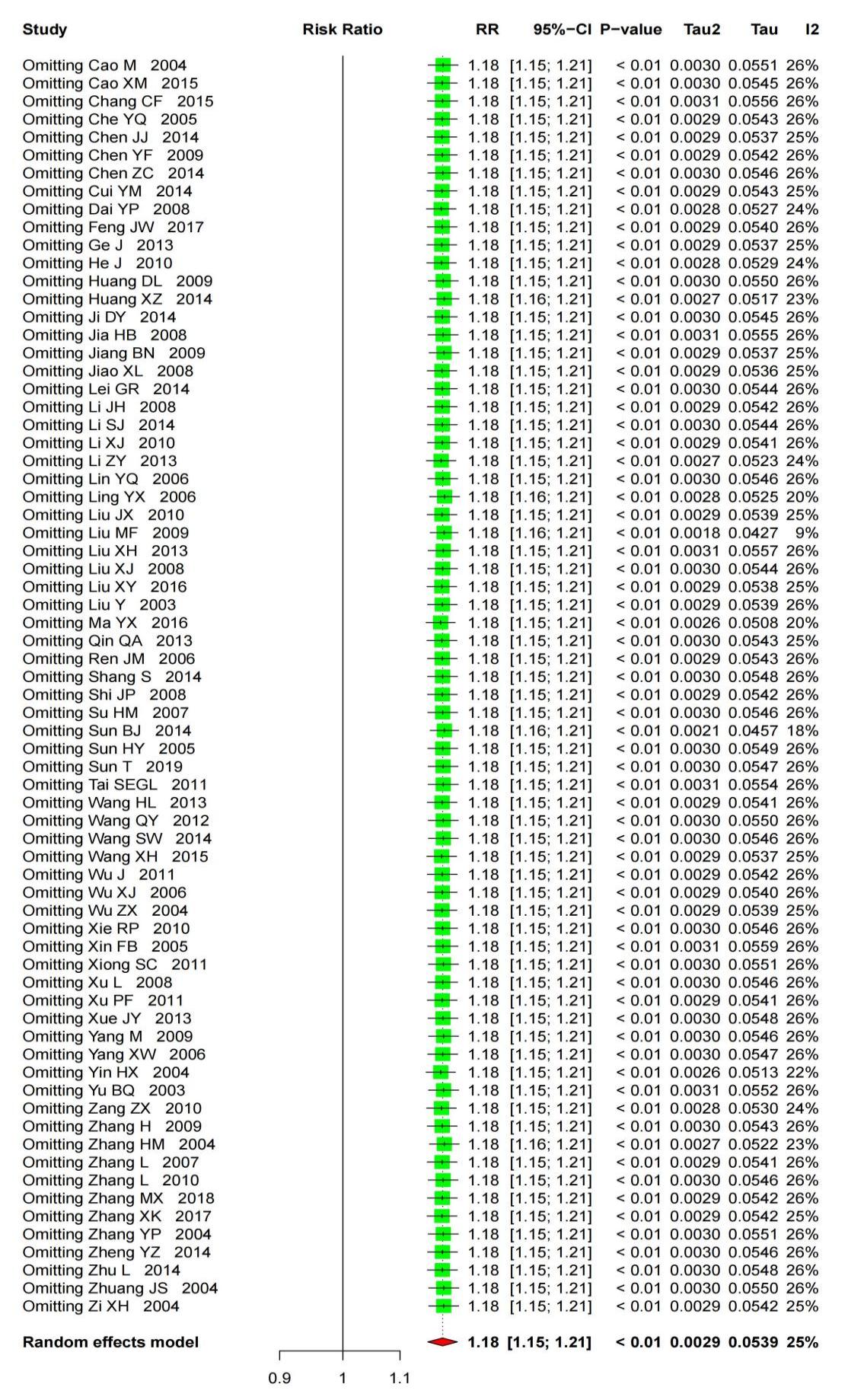


Note: CER, clinical effective rate; CTs, conventional treatments; SXNI, shuxuening injection.

# **sFigure 5. The results of subgroup analysis based on daily doses of SXNI (SXNI plus CTs vs. CTs plus other injections: CER).**


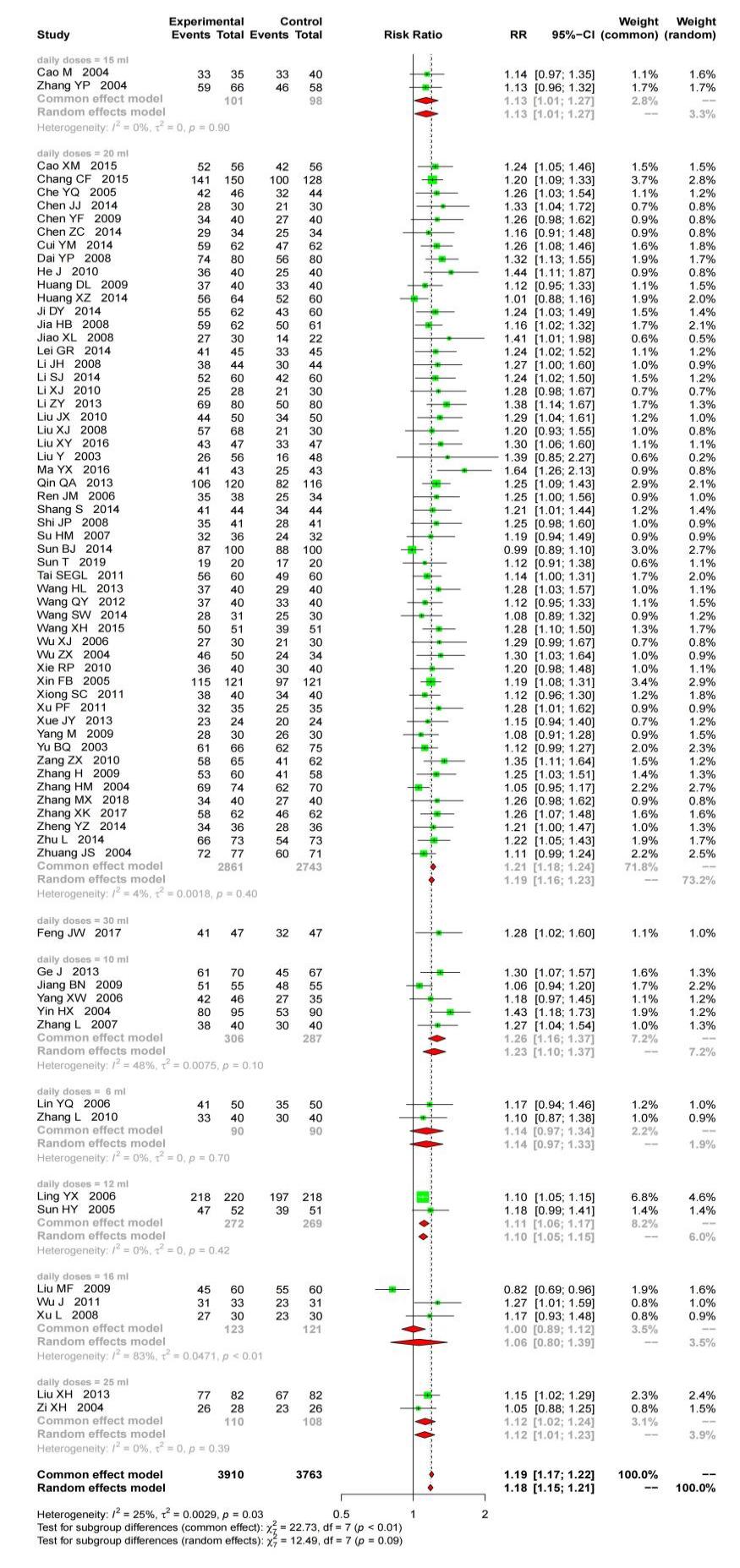


Note: CER, clinical effective rate; CTs, conventional treatments; SXNI, shuxuening injection.

# **sFigure 6. The results of subgroup analysis based on the intervention duration of SXNI (SXNI plus CTs vs. CTs plus other injections: CER).**


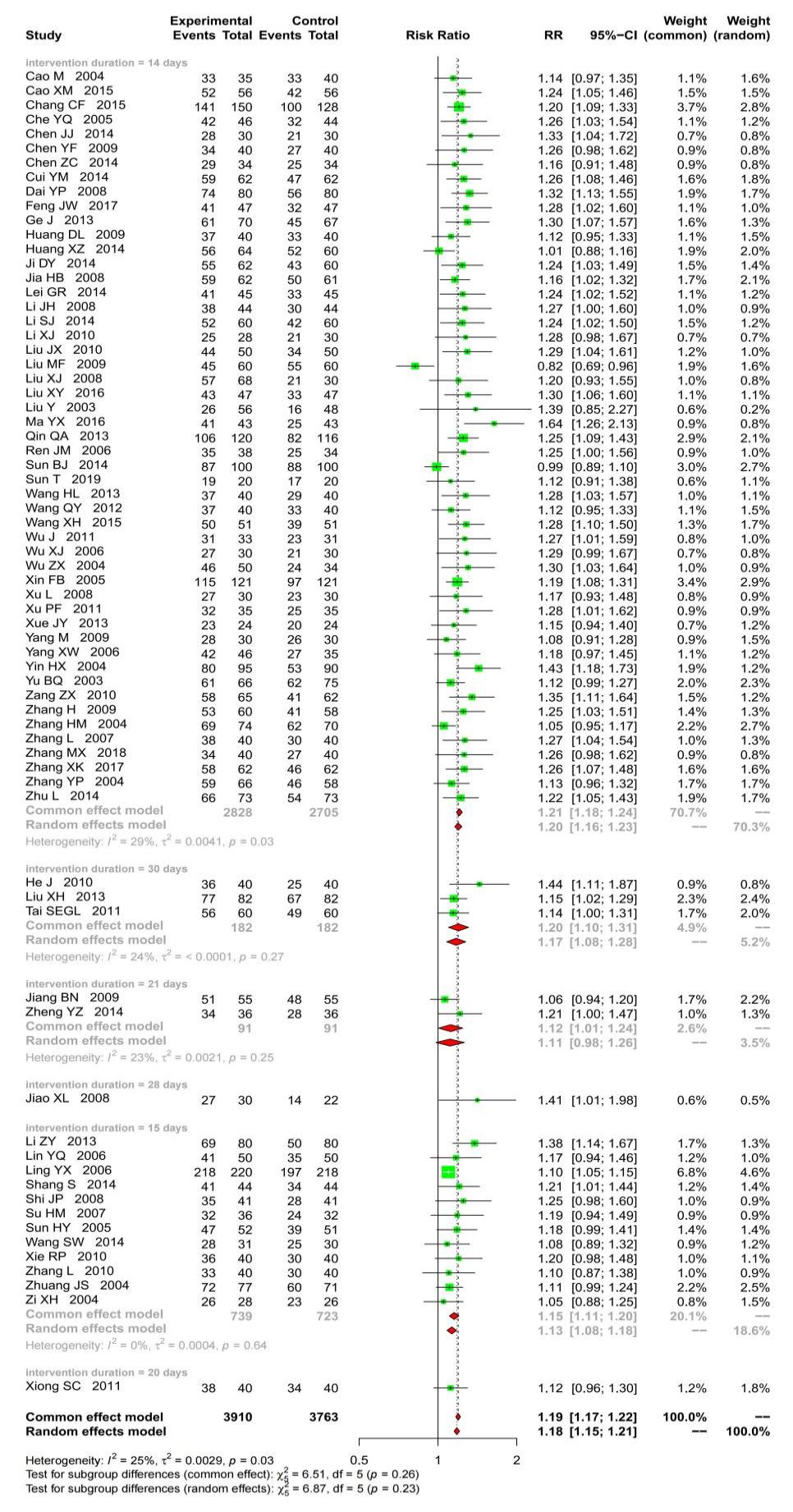


Note: CER, clinical effective rate; CTs, conventional treatments; SXNI, shuxuening injection.

# **sFigure 7. The results of subgroup analysis based on different injections in the control group (SXNI plus CTs vs. CTs plus other injections: CER).**


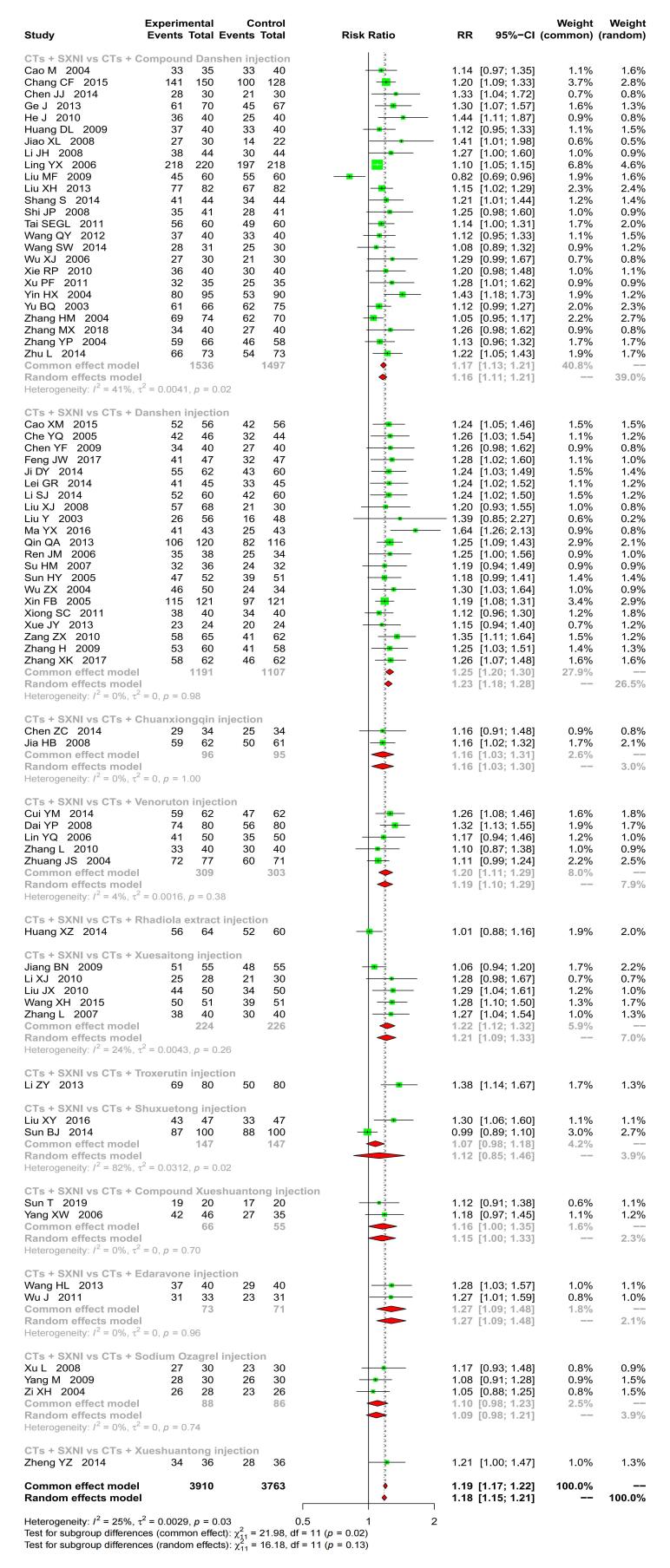


Note: CER, clinical effective rate; CTs, conventional treatments; SXNI, shuxuening injection.

# **sFigure 8. The results of the sensitivity analysis after removing studies one by one (SXNI plus CTs vs. CTs alone: NIHSS).**


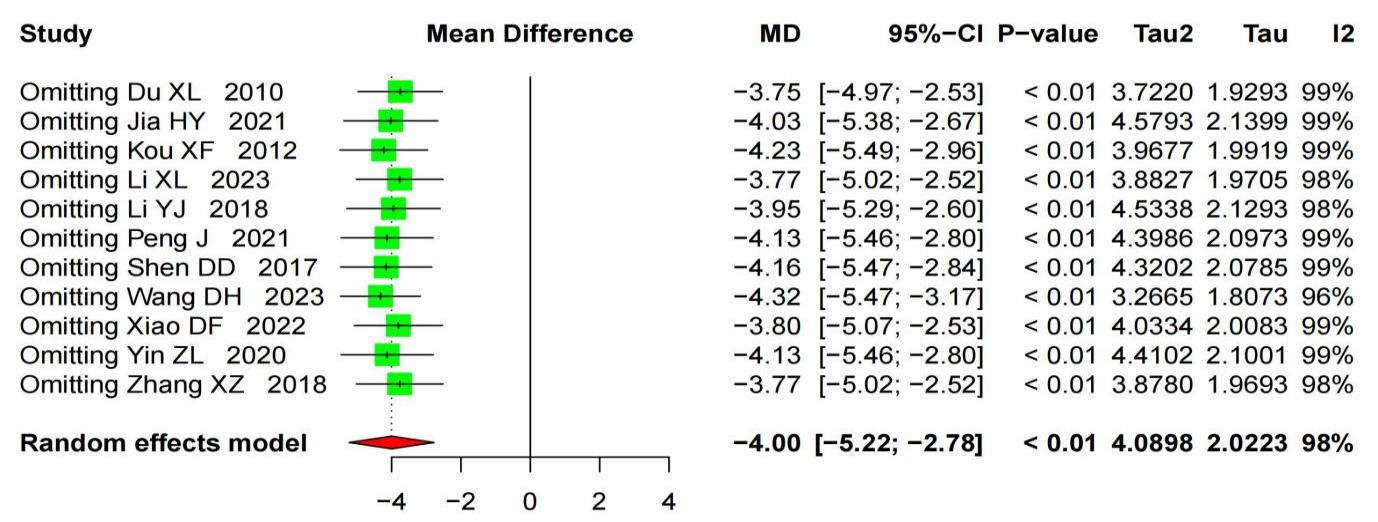


Note: CTs, conventional treatments; NIHSS, National Institutes of Health Stroke Scale; SXNI, shuxuening injection.

# **sFigure 9. The results of the sensitivity analysis after removing studies one by one (SXNI plus CTs vs. CTs alone: CCS).**


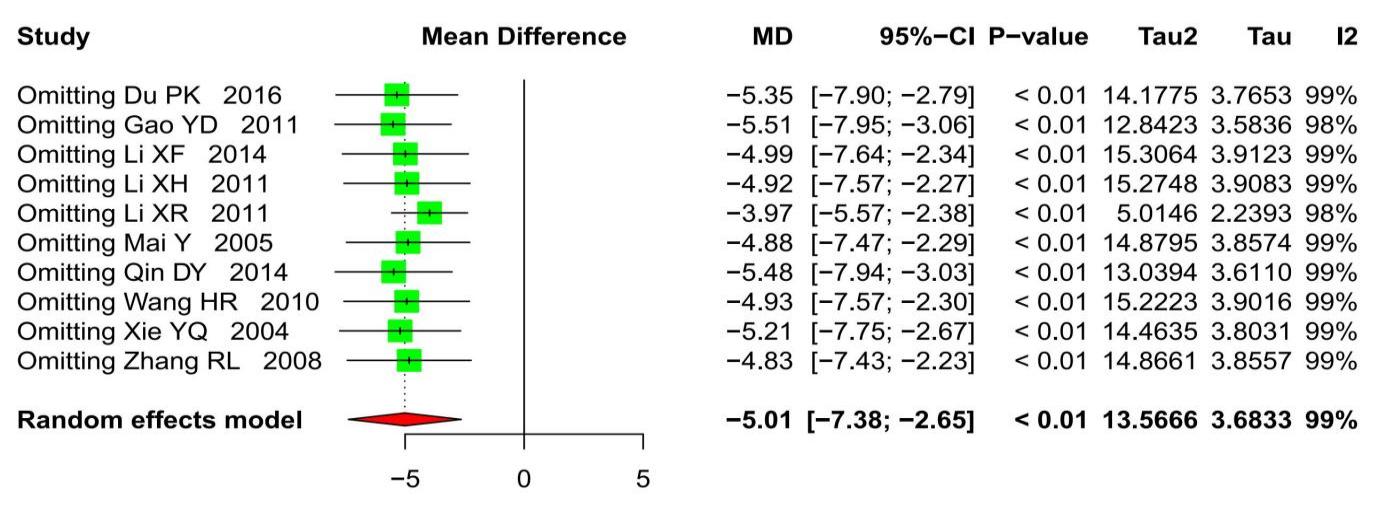


Note: CSS, Chinese Stroke Scale; CTs, conventional treatments; SXNI, shuxuening injection.

# **sFigure 10. The results of the sensitivity analysis after removing studies one by one (SXNI plus CTs vs. CTs plus other injection: NIHSS).**


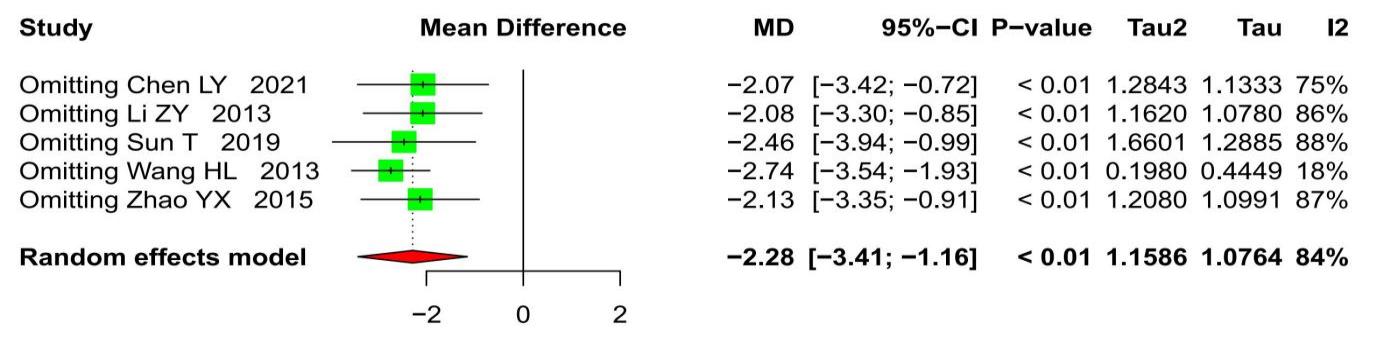


Note: CTs, conventional treatments; NIHSS, National Institutes of Health Stroke Scale; SXNI, shuxuening injection.

# **sFigure 11. The results of the sensitivity analysis after removing studies one by one (SXNI plus CTs vs. CTs plus other injection: CSS).**

**
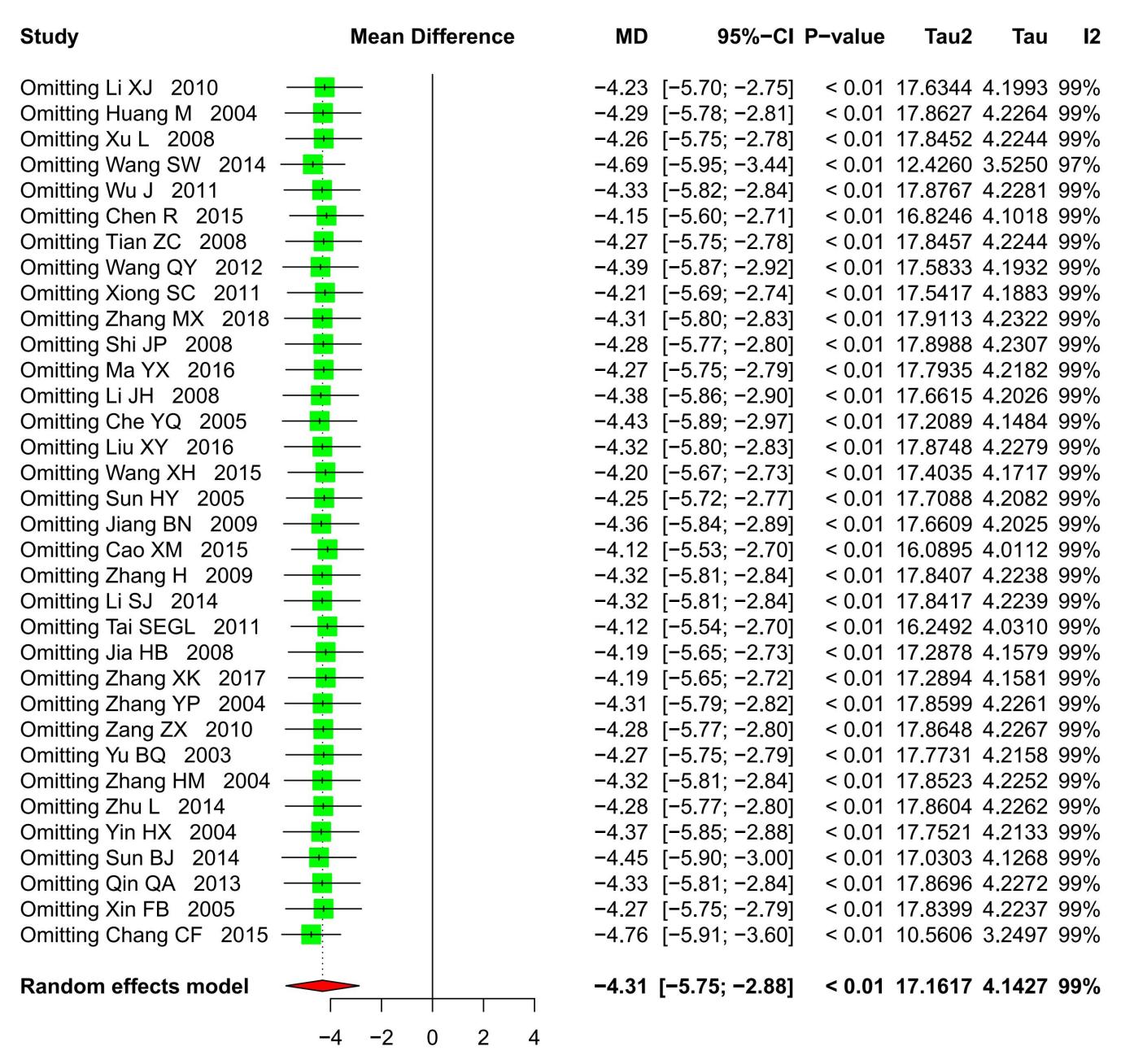
**

Note: CSS, Chinese Stroke Scale; CTs, conventional treatments; SXNI, shuxuening injection.

# **sFigure 12. The results of subgroup analysis based on daily doses of SXNI (SXNI plus CTs vs. CTs plus other injections: CSS).**


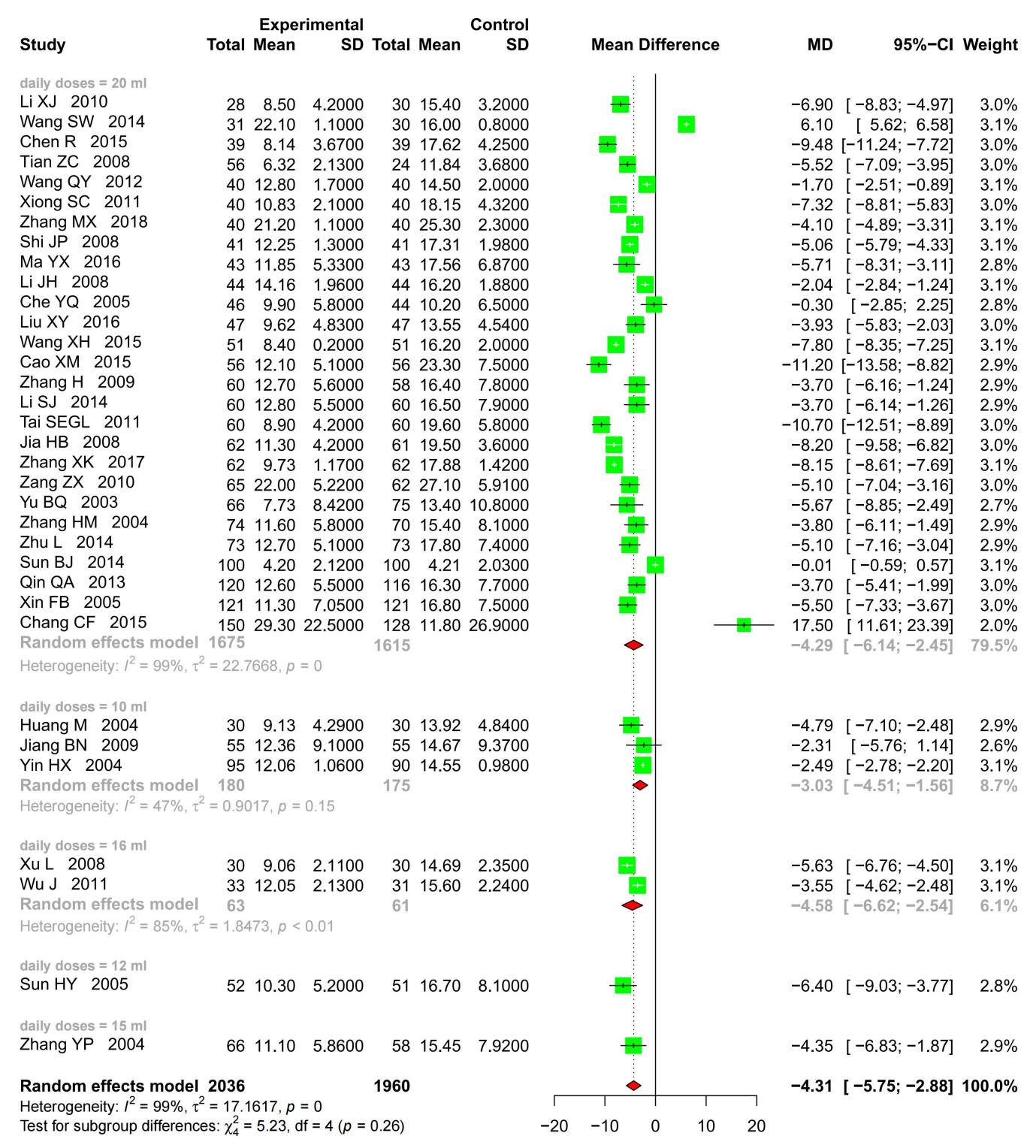


Note: CSS, Chinese Stroke Scale; CTs, conventional treatments; SXNI, shuxuening injection.

# **sFigure 13. The results of subgroup analysis based on the intervention duration of SXNI (SXNI plus CTs vs. CTs plus other injections: CSS).**


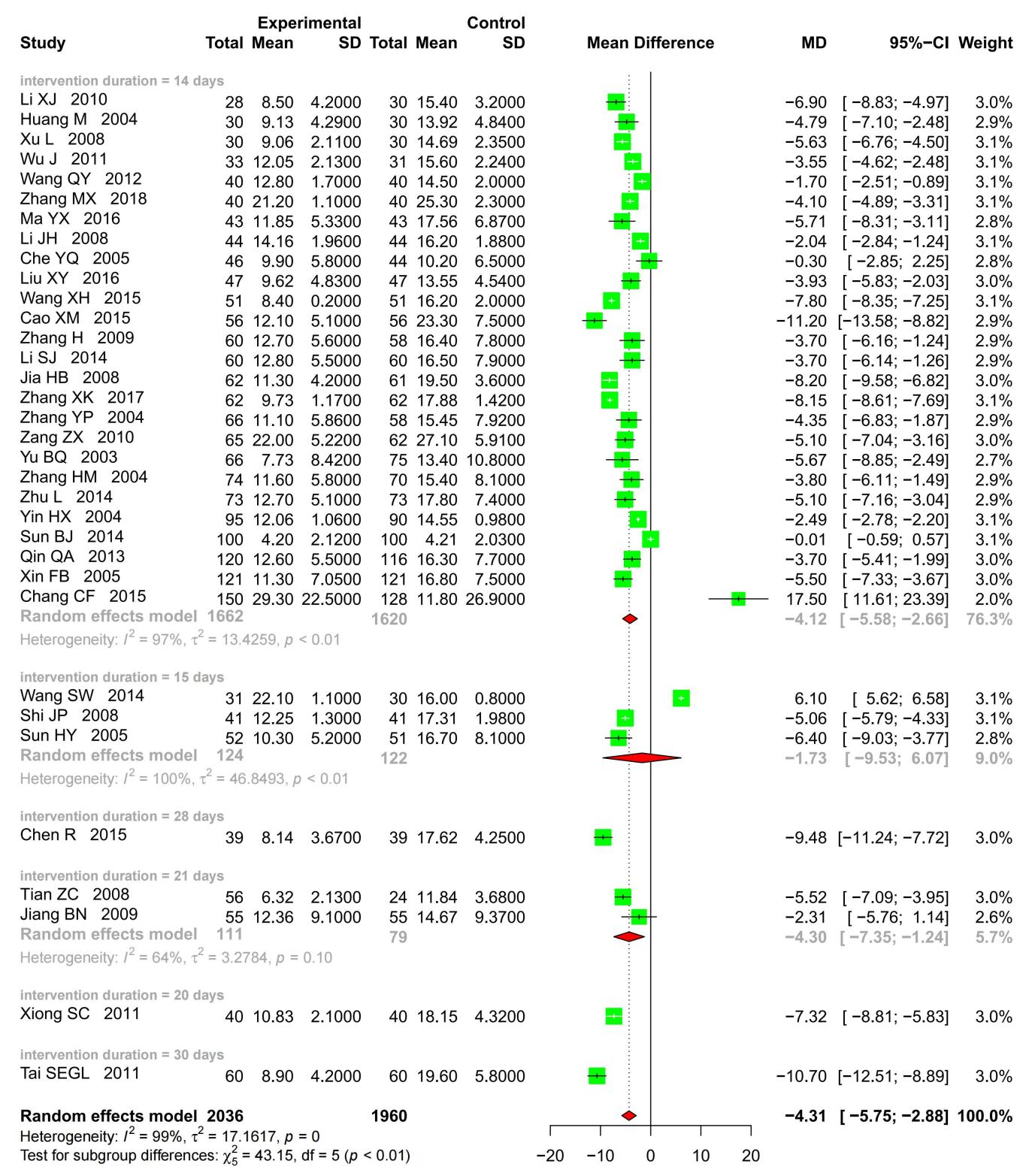


Note: CSS, Chinese Stroke Scale; CTs, conventional treatments; SXNI, shuxuening injection.

# **sFigure 14. The results of subgroup analysis based on different injections in the control group (SXNI plus CTs vs. CTs plus other injections: CSS).**

**
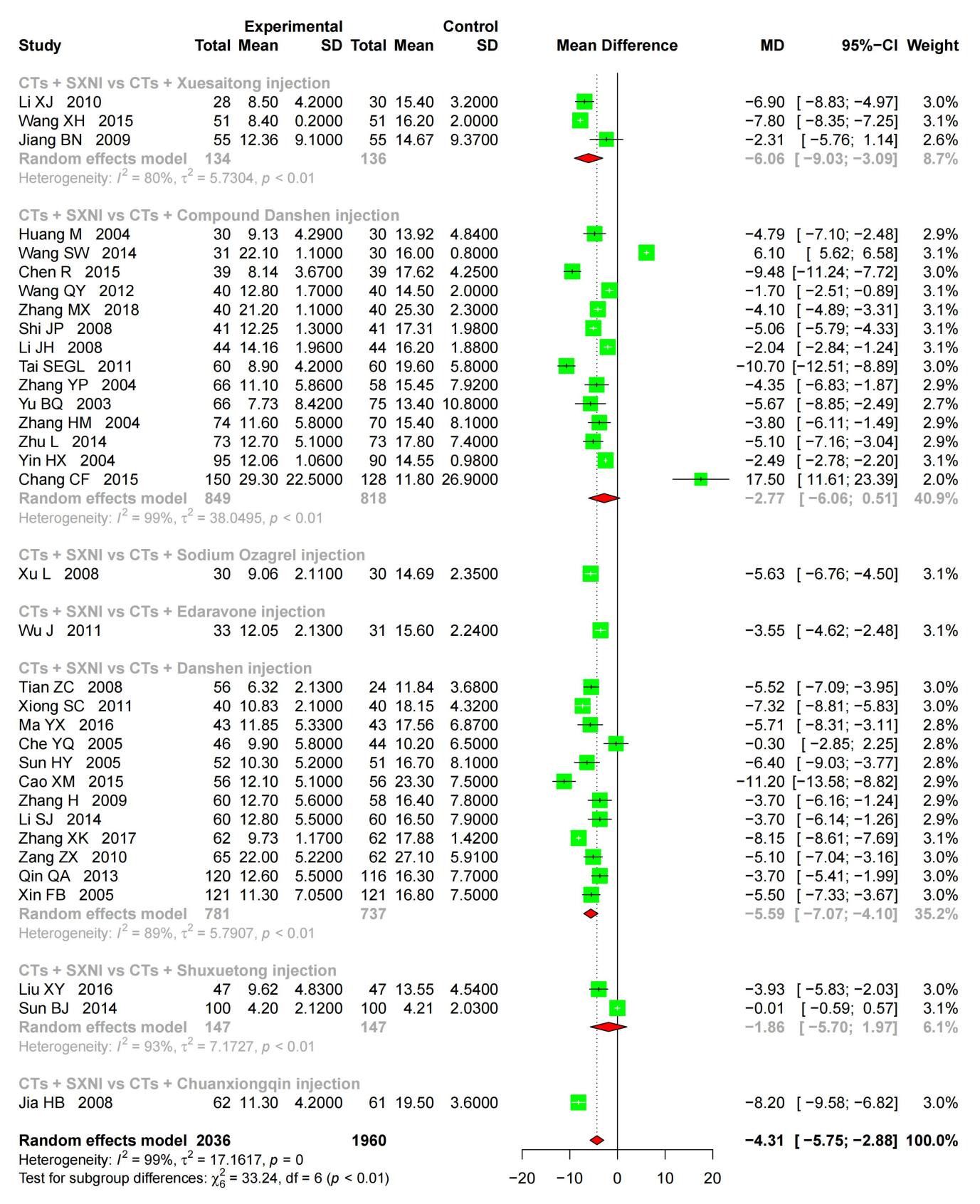
**

Note: CSS, Chinese Stroke Scale; CTs, conventional treatments; SXNI, shuxuening injection.

# **sFigure 15. The results of the sensitivity analysis after removing studies one by one (SXNI plus CTs vs. CTs alone: BI).**


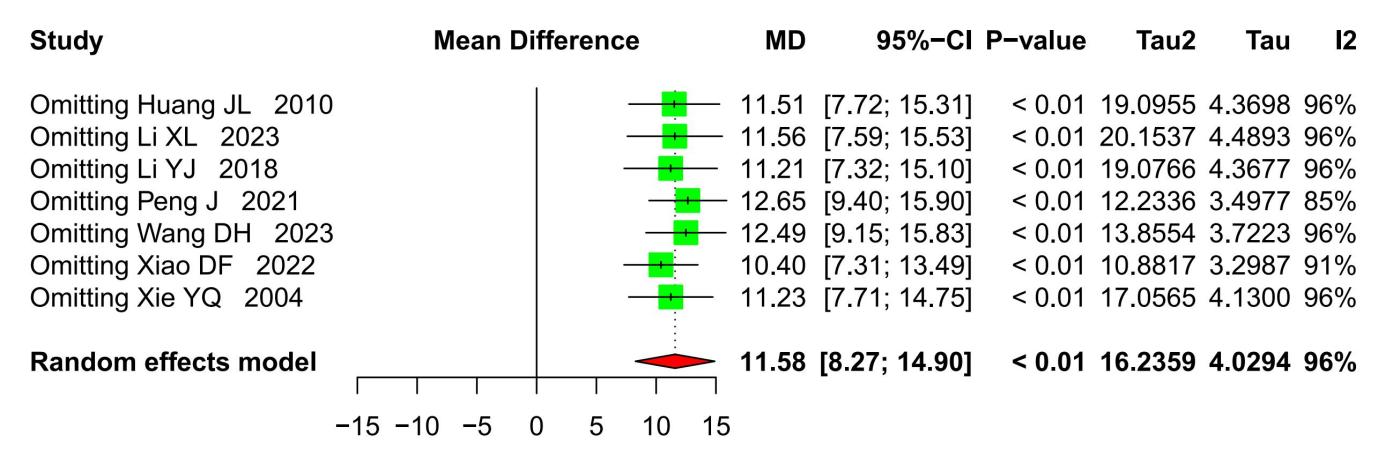


Note: BI, Barthel index; CTs, conventional treatments; SXNI, shuxuening injection.

# **sFigure 16. The results of the sensitivity analysis after removing studies one by one (SXNI plus CTs vs. CTs plus other injection: BI).**


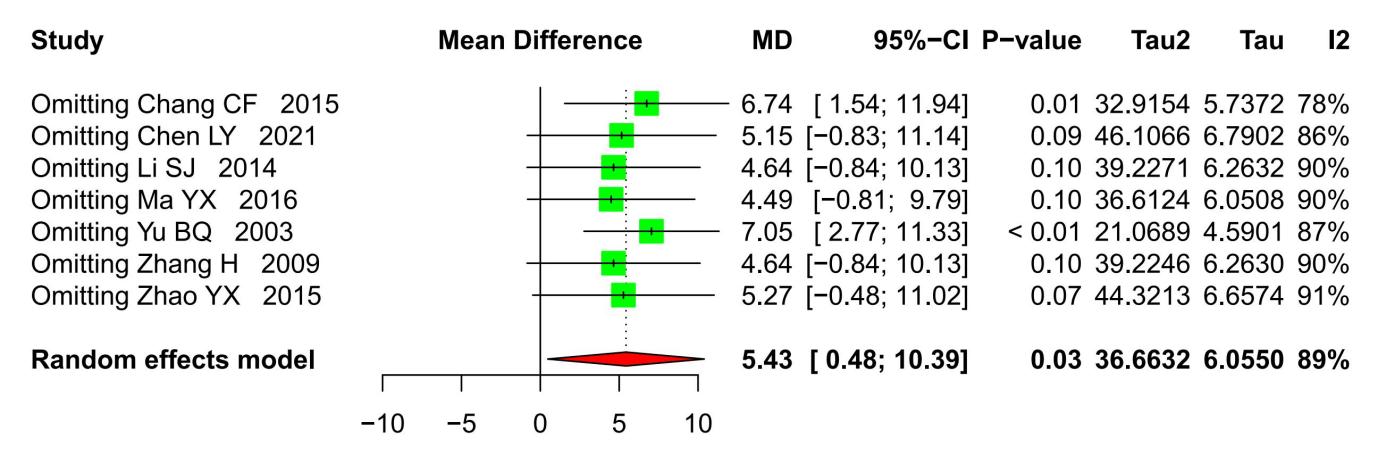


Note: BI, Barthel index; CTs, conventional treatments; SXNI, shuxuening injection.

# **sAppendix 1. Search Strategy**

**Cochrane Library：**

1. (stroke):ti,ab,kw (Word variations have been searched)
2. (palsy):ti,ab,kw (Word variations have been searched)
3. (apoplexy):ti,ab,kw (Word variations have been searched)
4. (apoplexia):ti,ab,kw (Word variations have been searched)
5. (cerebrovascular disorders):ti,ab,kw (Word variations have been searched)
6. (cerebrovascular disease):ti,ab,kw (Word variations have been searched)
7. (cerebrovascular accident):ti,ab,kw (Word variations have been searched)
8. (cva):ti,ab,kw (Word variations have been searched)
9. (cerebral infraction):ti,ab,kw (Word variations have been searched)
10. (infarct of brain):ti,ab,kw (Word variations have been searched)
11. (cerebral hemorrhage):ti,ab,kw (Word variations have been searched)
12. (intracerebral hemorrhage):ti,ab,kw (Word variations have been searched)
13. (brain hemorrhage):ti,ab,kw (Word variations have been searched)
14. #1 OR #2 OR #3 OR #4 OR #5 OR #6 OR #7 OR #8 OR #9 OR #10 OR #11 OR #12 OR #13
15. (Chinese patent medicine):ti,ab,kw (Word variations have been searched)
16. (shuxuening):ti,ab,kw (Word variations have been searched)
17. (shuxuening injection):ti,ab,kw (Word variations have been searched)
18. Shu-xue-ning injection):ti,ab,kw (Word variations have been searched)
19. (shu xue ning):ti,ab,kw (Word variations have been searched)
20. (Shu-xue-ning):ti,ab,kw (Word variations have been searched)
21. (SXN):ti,ab,kw (Word variations have been searched)
22. (SXN injection):ti,ab,kw (Word variations have been searched)
23. (Ginkgo Leaf):ti,ab,kw (Word variations have been searched)
24. (Folium Ginkgo):ti,ab,kw (Word variations have been searched)13
25. #15 OR #16 OR #17 OR #18 OR #19 OR #20 OR #21 OR #22 OR #23 OR #24
26. #25 AND #14 with Cochrane Library publication date Between Jan 1900 and Jan 2023

**PubMed：**

((((((((((((((stroke[MeSH Major Topic])) OR ((palsy[MeSH Major Topic]))) OR ((apoplexy[MeSH Major Topic]))) OR ((apoplexia[MeSH Major Topic]))) OR ((cerebrovascular disorders[MeSH Major Topic]))) OR ((cerebrovascular disease[MeSH Major Topic]))) OR ((cerebrovascular accident[MeSH Major Topic]))) OR ((cva[MeSH Major Topic]))) OR ((cerebral infraction[MeSH Major Topic]))) OR ((infarct of brain[MeSH Major Topic]))) OR ((cerebral hemorrhage[MeSH Major Topic]))) OR ((intracerebral hemorrhage[MeSH Major Topic]))) OR ((brain hemorrhage[MeSH Major Topic]))) AND (((((((((((Chinese patent medicine[MeSH Major Topic])) OR ((shuxuening[Title/Abstract]))) OR ((shuxuening injection[Title/Abstract]))) OR ((Shu-xue-ning injection[Title/Abstract]))) OR ((shu xue ning[Title/Abstract]))) OR ((Shu-xue-ning[Title/Abstract]))) OR ((SXN[Title/Abstract]))) OR ((SXN injection[Title/Abstract]))) OR ((Ginkgo Leaf[Title/Abstract]))) OR ((Folium Ginkgo[Title/Abstract]))) AND ((1996/01/01[PDAT] : 2023/01/31[PDAT]))

**Embase：**

1. stroke:ti,ab,kw
2. palsy:ti,ab,kw
3. apoplexy:ti,ab,kw
4. apoplexia:ti,ab,kw
5. 'cerebrovascular disorders':ti,ab,kw
6. 'cerebrovascular disease':ti,ab,kw
7. cva:ti,ab,kw
8. 'cerebrovascular accident':ti,ab,kw
9. 'cerebral infraction':ti,ab,kw
10. 'infarct of brain':ti,ab,kw
11. 'cerebral hemorrhage':ti,ab,kw
12. 'intracerebral hemorrhage':ti,ab,kw
13. 'brain hemorrhage':ti,ab,kw
14. #1 OR #2 OR #3 OR #4 OR #5 OR #6 OR #7 OR #8 OR #9 OR #10 OR #11 OR #12 OR #13
15. 'chinese patent medicine':ti,ab,kw
16. shuxuening:ti,ab,kw
17. 'shuxuening injection':ti,ab,kw
18. 'shu-xue-ning injection':ti,ab,kw
19. 'shu xue ning':ti,ab,kw
20. 'shu-xue-ning':ti,ab,kw
21. 'sxn injection':ti,ab,kw
22. sxn:ti,ab,kw
23. 'ginkgo leaf':ti,ab,kw
24. 'folium ginkgo':ti,ab,kw
25. #15 OR #16 OR #17 OR #18 OR #19 OR #20 OR #21 OR#22 OR #23 OR #24
26. #14 AND #25 AND [01-01-1966]/sd NOT[01-02-2023]/sd

**CNKI：**

( ( ( 主题%= 中风 + 卒中 + 脑血管病 + 脑血管意外 + 脑梗死 + 脑梗塞 + 脑栓塞 + 脑血栓 + 脑出血 + 脑溢血 or 题名%= 中风 + 卒中 + 脑血管病 + 脑血管意外 + 脑梗死 + 脑梗塞 + 脑栓塞 + 脑血栓 + 脑出血 + 脑溢血 ) AND ( 主题%= 舒血宁 + 银杏叶提取物 or 题名%= 舒血宁 + 银杏叶提取物 ) ) AND ( ( 发表时间 Between('1915-01-01','2023-01-31') )

**CBM：**

(("舒血宁注射液"[不加权:扩展]) AND ((((((("中风"[不加权:扩展] OR "卒中"[不加权:扩展] OR "中风康复"[不加权:扩展] OR "中风后遗症"[不加权:扩展] OR "梗死, 大脑后动脉"[不加权:扩展] OR "梗死, 大脑前动脉"[不加权:扩展]) OR "梗死, 大脑中动脉"[不加权:扩展] OR "梗死, 大脑前动脉"[不加权:扩展]) OR "垂体卒中"[不加权:扩展] OR "缺血性卒中"[不加权:扩展] OR "出血性卒中"[不加权:扩展] OR "栓塞性卒中"[不加权:扩展]) OR "血栓性卒中"[不加权:扩展]) OR "脑血管障碍"[不加权:扩展]) OR "脑梗死"[不加权:扩展] OR "大脑梗死"[不加权:扩展]) OR "颅内栓塞"[不加权:扩展] OR "颅内栓塞和血栓形成"[不加权:扩展])) AND -2023[日期]

**VIP：**

((((((((((题名或关键词=中风 OR 题名或关键词=卒中) OR 题名或关键词=脑血管病) OR 题名或关键词=脑血管意外) OR 题名或关键词=脑梗死) OR 题名或关键词=脑梗塞) OR 题名或关键词=脑栓塞) OR 题名或关键词=脑血栓) OR 题名或关键词=脑出血) OR 题名或关键词=脑溢血) AND (题名或关键词=舒血宁 OR 题名或关键词=银杏叶提取物))

**Wan Fang：**

 (((((((((题名或关键词=卒中 OR 题名或关键词=中风) OR 题名或关键词=脑梗死) OR 题名或关键词=脑栓塞) OR 题名或关键词=脑出血) OR 题名或关键词=脑血管病) OR 题名或关键词=脑血管意外) OR 题名或关键词=脑溢血) OR 题名或关键词=脑梗塞) OR 题名或关键词=脑血栓) AND 题名或关键词=舒血宁 ) OR 题名或关键词=银杏叶提取物)) and Date:1900-2023

# **sAppendix 2. The full citation details of the included and excluded studies**

**Included studies for qualitative and quantitative synthesis (n=116):**

1. Cao M. Clinical analysis of 35 cases of cerebral infarction treated with Shuxuening injection. Chinese General Practice. 2004;7(6):398. doi:10.3969/j.issn.1007-9572.2004.06.037.
2. Cao XM. Clinical efficacy of Shuxuening in the treatment of acute cerebral infarction and its effect on patients' neurological function recovery. Chinese Journal of Practical Nervous Diseases. 2015;18(18):125-126. doi: 10.3969/j.issn.1673-5110.2015.18.097.
3. Chang CF. Clinical observation on the treatment of acute cerebral infarction with Shuxuening Injection. Journal of Frontiers of Medicine. 2015;5(13):122-123. doi:10.3969/j.issn.2095-1752.2015.13.115.
4. Che YQ. Effects of the Shuxuening parenteral solution for content of the S-100 beta and neuron specific enolase protein of plasm in cerebral infarction. Journal of Medical Science Yanbian University. 2005;28(01):56-59. doi:10.3969/j.issn.1000-1824.2005.01.018.
5. Chen JJ. Clinical experience of 30 cases of acute cerebral infarction treated by Shuxuening. Heilongjiang Journal of Traditional Chinese Medicine. 2014;43(5):38-39. doi:CNKI:SUN:HLZY.0.2014-05-026
6. Chen LY. Clinical effect of Shuxuening injection in the adjuvant treatment of acute cerebral infarction. Chinese Journal of Clinical Rational Drug Use. 2021;14(22):47-49. doi:10.15887/j.cnki.13-1389/r.2021.22.017.
7. Chen R. Study on the efficacy of Shuxuening in the treatment of cerebral infarction and its effect on blood rheology. Heilongjiang Medicine Journal. 2015;28(03):554-555. doi:10.14035/j.cnki.hljyy.2015.03.046.
8. Chen YF, Wang HP, Yang H. Clinical Observation on the Effect of Shuxuening Injection on Senile Ischemic Cerebrovascular Disease. Guide of China Medicine. 2009;7(07):26-27. doi:10.3969/j.issn.1671-8194.2009.07.013.
9. Chen ZC, Zheng GY, Ye ZW, Chen ZH. Observation on the therapeutic effect of Shuxuening Injection in the treatment of acute cerebral infarction with evidence of blood stasis obstruction. Practical Journal of Cardiac Cerebral Pneumal and Vascular Disease. 2014;22(04):93-94. doi:10.3969/j.issn.1008-5971.2014.04.048.
10. Cheng ZL, Jin L. Effect of Shuxuening injection on nitric oxide and coagulation function in patients with acute cerebral infarction. Chinese Journal of Practical Nervous Diseases. 2016;19(6):99-100. doi:10.3969/j.issn.1673-5110.2016.06.067.
11. Cui YM. Efficacy and mechanism of Shuxuening injection in the treatment of acute cerebral infarction. Guide of China Medicine. 2014;12(8):177-178. doi:10.15912/j.cnki.gocm.2014.08.184.
12. Dai YP. Clinical observation of Shuxuening in the treatment of 80 cases of acute cerebral infarction. Journal of Chinese Practical Diagnosis and Therapy. 2008;22(10):770-771. doi:10.3969/j.issn.1674-3474.2008.10.022.
13. Du PK, Wang YL, Zhang XH. Clinical efficacy of Shuxuening in the treatment of acute cerebral infarction and its influence on the recovery of neurological function. Chin J Mod Drug Appl. 2016;10(12):129-130. doi:10.14164/j.cnki.cn11-5581/r.2016.12.096.
14. Du XL, Li YB, Li D, Li BZ, Xu CM, Tang P. Clinical study of Shuxuening injection for treating patients with acute cerebral infarction. Chinese Journal of New Drugs. 2010;19(11):963-965. doi: CNKI:SUN:ZXYZ.0.2010-11-024.
15. Feng JW. Effect of Shuxuening Injection on the treatment and hemorheology of patients with ischemic stroke. Guide of China Medicine. 2017;15(29):202-203. doi:10.15912/j.cnki.gocm.2017.29.163.
16. Gao YD. Clinical effect of Shuxuening injection in the treatment of 60 cases of acute cerebral infarction. Journal of Modern Medicine & Health. 2011;27(1):101-102. doi:CNKI:SUN:XYWS.0.2011-01-075.
17. Ge J. Clinical observation of Shuxuening injection in the treatment of acute cerebral Infarction. Chinese Journal of Clinical Rational Drug Use. 2013;6(02):49-50. doi:10.15887/j.cnki.13-1389/r.2013.02.128.
18. Guo WJ, Bian HR, Tian ZK, Wang X, Wang YX, Zhao YF. Effect of Shuxuening Injection on serum TNF-α and ET-1 in patients with acute cerebral infarction. Modern Journal of Integrated Traditional Chinese and Western Medicine. 2013;22(25):2744-2745+2748. doi:10.3969/j.issn.1008-8849.2013.25.004.
19. He J, Hu RP. Shuxuening injection treating 40 cases of acute cerebral infarction. Journal of Emergency in Traditional Chinese Medicine. 2010;19(12):2130. doi:10.3969/j.issn.1004-745X.2010.12.064.
20. He JQ. The Mechanism and Prospective Study on Shuxuening Injection in the Treatment of Senile Ischemic Cerebrovascular Disease. Clinical Medicine & Engineering. 2013;20(7):849-850. doi:10.3969/j.issn.1674-4659.2013.07.0849.
21. He XY, Wang WH, Wu Y, Ma M. Clinical observation and nursing care of Shuxuening injection in the treatment of elderly ischemic cerebrovascular disease. Journal of Military Surgeon in Southwest China. 2006;8(06):115-116. doi:10.3969/j.issn.1672-7193.2006.06.068.
22. Hua GC, Feng QG, Fan HC. Investigation on Clinical Effect of Shuxuening Injection for Ischemic Apoplexy (Cerebral Infarction) on 60 Cases. Chinese Traditional Patent Medicine. 2002;24(01):36-38. doi:10.3969/j.issn.1001-1528.2002.01.014.
23. Huang DL, Zhang HX. Clinical Observation of Shuxuening Injection Treated on 80 Cases with Acute Cerebral Infarction. The Journal of Medical Theory and Practice. 2009;22(06):621-624. doi:10.3969/j.issn.1001-7585.2009.06.001.
24. Huang JL. Effect of Shuxuening injection on acute cerebral infarction. China Medicine. 2010;5(12):1132-1133. doi:10.3760/cma.j.issn.1673-4777.2010.12.010.
25. Huang M, Guan CH. Shuxuening in the treatment of acute cerebral infarction. Chinese Journal of Thrombosis and Hemostasis. 2004;10(01):29-30. doi:10.3969/j.issn.1009-6213.2004.01.012.
26. Huang XZ, Xie LY, Xu HY. Shuxuening injection compared with Dazhu Rhodiola injection in treatment of elderly patients with acute cerebral infarction. Journal of Frontiers of Medicine. 2014;2014(27):29-30. doi:10.3969/j.issn.2095-1752.2014.27.019.
27. Ji DY. Efficacy of Shuxuening injection in the treatment of acute cerebral infarction. Chinese Journal of Trauma and Disability Medicine. 2014;22(3):121-122. doi:10.13214/j.cnki.cjotadm.2014.03.116.
28. Jia HB. Efficacy of Shuxuening for acute cerebral infarction with syndrome of static blood blocking collaterals. Lishizhen Medicine and Materia Medica Research. 2008;19(01):201-202. doi:10.3969/j.issn.1008-0805.2008.01.115.
29. Jia HY, Wang QF. Study on the efficacy of Shuxuening injection in the treatment of acute cerebral infarction. China Health Care & Nutrition. 2021;31(22):22.
30. Jiang BN, Wang JR. Curative effect of Shuxuening injection on acute cerebral infarction. Zhejiang Clinical Medicine Journal. 2009;11(5):518-519. doi:10.3969/j.issn.1008-7664.2009.05.034.
31. Jiao XL, Ji JY. Effect of Shuxuening injection on cerebral infarction. Northwest Pharmaceutical Journal. 2008;23(06):391-392. doi:10.3969/j.issn.1004-2407.2008.06.035.
32. Kou XF. Clinical efficacy of Shuxuening injection and impact on recovery of neurological function in patient with acute ischemic stroke. Modern Preventive Medicine. 2012;39(20):5494-5495. doi:CNKI:SUN:XDYF.0.2012-20-117.
33. Lei GR. Observation on the clinical effect of Shuxuening injection in the treatment of acute cerebral infarction. Journal of Medical Information. 2014;2014(37):48-49. doi:10.3969/j.issn.1006-1959. 2014.37.062.
34. Li JH, Chen L, Hou HG. Clinical evaluation on treatment of elderly 44 cases patients with acute cerebral infarction with Shuxuening injection. The Medical Forum. 2008;12(22):684-685. doi:10.3969/j.issn.1672-1721.2008.22.007.
35. Li SJ. Clinical effect of Shuxuening injection in the treatment of acute cerebral infarction. Jilin Medical Journal. 2014;35(12):2560. doi:10.3969/j.issn.1004-0412.2014.12.050
36. Li XF. Shuxuening Injection has a good effect on nerve function deficit in patients with acute cerebral infarction. Chinese Journal of Practical Nervous Diseases. 2014;2014(17):61-62. doi:10.3969/j.issn.1673-5110.2014.17.038.
37. Li XH. Clinical effect of Shuxuening injection in the treatment of acute cerebral infarction. Asia-Pacific Traditional Medicine. 2011;7(9):129-130.
38. Li XJ. The therapeutic effects of Shuxuening injectionin acute cerebral infarction patients. China Modern Medicine. 2010;17(11):41-42. doi:10.3969/j.issn.1674-4721.2010.11.022.
39. Li XL, Dai ZC. Study of Shuxuening Injection in adjuvant treatment of acute cerebral infarction and improvement of nerve function deficit and blood coagulation function in patients. Modern Medicine and Health Research Electronic Journal. 2023;7(01):80-83. doi:10.3969/j.issn.2096-3718.2023.01.024.
40. Li XR, Wang ZL. Shuxuening adjuvant therapy for middle cerebral artery occlusion. Chinese Journal of Experimental Traditional Medical Formulae. 2011;17(11):257-258. doi:10.3969/j. issn.1005-9903.2011.11.075.
41. Li YJ. Effects of Shuxuening on the clinical efficacy of patients with cerebral infarction and the levels of plasma Hcy, UCH-L1, fibulin-5. Anhui Medical and Pharmaceutical Journal. 2018;22(01):143-146. doi:10.3969/j.issn.1009-6469.2018.01.037.
42. Li ZY, Ma HD. Analysis of curative effect of Shuxuening Injection in treating acute cerebral infarction and its effect on the recovery of nerve function. World Latest Medicine Information. 2013;2013(10):12-13. doi:10.3969/j.issn.1671-3141.2013.10.005.
43. Lin YQ. Effect of Shuxuening injection on acute cerebral infarction. Modern Journal of Integrated Traditional Chinese and Western Medicine. 2006;15(06):700. doi:10.3969/j.issn.1008-8849.2006.06.007.
44. Ling YX, Wang ZH, Chen WR, Chen ZY. Effect of ginkgo leaf injection on acute cerebral infarction. Journal of Guangdong Medical University. 2006;24(05):466-468. doi:10.3969/j.issn.1005-4057.2006.05.011.
45. Liu JX, Ding XR, Feng LJ, Cao LP. Clinical observation of Shu Xue Ning to the function rehabilitation of cerebroinfarction patients. China New Medicine. 2004;3(1):41-42.
46. Liu JX, Liu YY, Jia Y. Clinical effect of Shuxuening injection on acute cerebral infarction. Chinese Journal of Urban and Rural Enterprise Hygiene. 2010;25(1):48. doi:10.16286/j.1003-5052.2010.01.020.
47. Liu MF. Observation on curative effect of Shuxuening injection on ischemic cerebrovascular disease (Report of 60 cases). Medical Information. 2009;22(04):365-366. doi:10.3969/j.issn.1006-1959-C.2009.04.036.
48. Liu S, Zhang DS, Jiang SX, Yu YY. Shuxuening injection in the treatment of 60 cases of acute cerebral infarction. China Pharmaceuticals. 2004;13(10):71. doi:10.3969/j.issn.1006-4931.2004.10.057.
49. Liu XH. Mechanism and prospective study of Shuxuening injection in treatment of elderly patients with ischemic cerebrovascular disease. Guide of China Medicine. 2013;11(22):271-272. doi:10.15912/j.cnki.gocm.2013.22.539.
50. Liu XJ, Cai XF. Effect of Shuxuening injection on 68 cases of ischemic cerebrovascular disease. Chinese Journal of Coal Industry Medicine. 2008;11(04):477-478. doi:10.3969/j.issn.1007-9564.2008.04.015.
51. Liu XY, Zhao LF. Clinical observation of Shuxuening injection in the treatment of acute ischemic stroke. World Latest Medicine Information. 2016;16(96):159-160.doi:CNKI:SUN:WMIA.0.2016-96-131.
52. Liu Y, Wang RF, Zhou NG, Lu J. Clinical observation of Shuxuening in the treatment of acute cerebral infarction. Pharmaceutical Care and Research. 2003;3(03):146. doi:10.3969/j.issn.1671-2838.2003.03.027.
53. Ma YX. Clinical effect and safety analysis of Shuxuening in the treatment of cerebral infarction. China Health Care & Nutrition. 2016;26(29):49. doi:10.3969/j.issn.1004-7484.2016.29.057.
54. Mai Y, Qu XW. Clinical observation of Shuxuening injection in treatment of 34 cases of acute cerebral infarction. Chinese Journal of Integrative Medicine on Cardio-Cerebrovascular Disease. 2005;3(3):277. doi:10.3969/j.issn.1672-1349.2005.03.055.
55. Nie XP. The clinical effect of Shuxuening injection in the treatment of ischemic cerebrovascular diseases. Chinese Journal of Clinical Rational Drug Use. 2020;13(09):50-51. doi:10.15887/j.cnki.13-1389/r.2020.09.023.
56. Peng J, Li JQ, Xiong YY. Effect of Shuxuening Injection on hemorheology and quality of life in patients with acute cerebral infarction. Chinese Journal of Integrative Medicine on Cardio-Cerebrovascular Disease. 2021;19(11):1914-1916. doi:10.12102/j.issn.1672-1349.2021.11.032.
57. Qin DY, Kang JL, Cheng C, Zhou LY, Feng JL, Zeng AY. Clinical efficacy of Shuxuening and its influence on the serum levels of IL-6, IL-8 and CRP in patients with acute cerebral infarction. Hainan Medical Journal. 2014;25(12):1769-1771. doi:10.3969/j.issn.1003-6350.2014.12.0687.
58. Qin QA. Curative effect of Shuxuening on acute cerebral infarction and its influence on neurological function recovery. Chinese Journal of Practical Nervous Diseases. 2013;16(03):40-41. doi:10.3969/j.issn.1673-5110.2013.03.021.
59. Ren JM. Clinical evaluation on treatment of acute cerebral infarction with Shuxuening Injection. Journal of Clinical and Experimental Medicine. 2006;5(12):1993. doi:10.3969/j.issn.1671-4695.2006.12.074.
60. Shang S. Clinical study on Shuxuening in the treatment of cerebral infarction. Guangming Journal of Chinese Medicine. 2014;29(08):1764-1765. doi:10.3969/j.issn.1003-8914.2014.08.103.
61. Shen DD. Effect of shuxuening injection in the adjuvant treatment of cerebral infarction and its clinical nursing. Chinese Journal of Biochemical and Pharmaceuticals. 2017;37(9):84-85,88. doi:10.3969/j.issn.1005-1678.2017.09.033.
62. Shi JP. Shuxuening injection for treatment of acute cerebral infarction. Journal of Medical Forum. 2008;29(11):78-79. doi:10.3969/j.issn.1672-3422.2008.11.048.
63. Su HM. Curative effect of Shuxuening Injection on 36 cases of acute ischemic stroke. Western Journal of Traditional Chinese Medicine. 2007;20(05):26-27. doi:10.3969/j.issn.1004-6852.2007.05.015.
64. Sun BJ. Comparison of curative effect of Shuxuening and Shuxuetong in the treatment of acute cerebral infarction. Modern Journal of Integrated Traditional Chinese and Western Medicine. 2014;23(31):3486-3487. doi:10.3969/j.issn.1008-8849.2014.31.028.
65. Sun HY, Lin HY. Effect of Shuxuening Injection on Acute Cerebral Infarction. Herald of Medicine. 2005;24(9):778-779. doi:10.3870/j.issn.1004-0781.2005.09.012.
66. Sun T. Effect of Shuxuening injection on acute cerebral infarction. Contemporary Medical Symposium. 2019;17(9):143-145. doi:10.3969/j.issn.2095-7629.2019.09.109.
67. Tai SEGL, Lin Y, Li HJ. Curative effect of Shuxuening injection on 60 cases of cerebral infarction. New Medicine. 2011;21(3):224-225. doi:10.3969/j.issn.1004-5511.2011.03.024.
68. Tian ZC, Feng XX, Shen M. Observation of curative effect of Shuxuening Injection on cerebral thrombosis. China Modern Doctor. 2008;46(01):83. doi:10.3969/j.issn.1673-9701.2008.01.054.
69. Wang DH, Ma N, Tian HY. Curative effect of Shuxuening injection in the treatment of acute cerebral infarction. Liaoning Journal of Traditional Chinese Medicine. 2023;50(04):68-71. doi:10.13192/j.issn.1000-1719.2023.04.019.
70. Wang HL, Liu LJ, Zhao SQ. Effect of Shuxuening Injection on VEGF，vWF and MMP-9 in Patients with Acute Cerebral Infarction. Chinese Journal of Experimental Traditional Medical Formulae. 2013;19(10):343-345. doi: 10.11653/syfj2013100343.
71. Wang HR. Effect of Shuxuening Injection on cerebral embolism. China Medicine. 2010;05(12):1130-1131. doi:10.3760/cma.j.issn.1673-4777.2010.12.009.
72. Wang JF. Clinical effect and nursing care of Shuxuening injection in adjuvant treatment of cerebral infarction. Electronic Journal of Clinical Medical Literature. 2018;5(26):146-148. doi:10.16281/j.cnki.jocml.2018.26.081.
73. Wang MS, Lin JY. Researches of Shuxuening on the Clinical Effects and Quality of Life in Old Patients with Ischemic Stoke. Chinese Journal of Arteriosclerosis. 2012;20(10):923-926. doi:CNKI:SUN:KDYZ.0.2012-10-015.
74. Wang QY, Song J. Shuxuening injection in the treatment of acute cerebral infarction: A clinical observation. Shanxi Medical Journal. 2012;41(8):807-808. doi:10.3969/j.issn.0253-9926.2012.08.037.
75. Wang SW. Clinical Observation of Shuxuening Injection in the treatment of cerebral Infarction. Chinese Journal of Trauma and Disability Medicine. 2014;22(8):128-129. doi:10.13214/j.cnki.cjotadm.2014.08.118.
76. Wang WH, Xu XH, Hu J, Zhong JY, Xue YQ, He XY, Luo R. Clinical effect of Shuxuening injection in treating senile ischemic cerebrovascular disease. Journal of Military Surgeon in Southwest China. 2006;8(01):17-18. doi:10.3969/j.issn.1672-7193.2006.01.007.
77. Wang XH. Effect analysis of Shuxuening Injection in treatment of 51 cases of acute cerebral infarction. E-Journal of Translational Medicine. 2015;2(9):42-43 doi:CNKI:SUN:ZHDZ.0.2015-09-024.
78. Wu J, Xu L. Clinical Observation of Shuxuening Injection in the Treatment of Acute Cerebral Ischemia. Acta Chinese Medicine. 2011;26(11):1362-1363. doi:10.16368/j.issn.1674-8999.2011.11.019.
79. Wu XJ. Curative effect of Shuxuening Injection on cerebral infarction with hyperlipidemia. Journal of Clinical Research. 2006;23(04):591-592. doi:10.3969/j.issn.1671-7171.2006.04.056.
80. Wu Y, Ye CJ, Wu F, Chen M, Chen SH. Shuxuening injection in the treatment of 30 cases of acute ischemic stroke. Journal of Emergency in Traditional Chinese Medicine. 2011;20(08):1305-1306. doi:10.3969/j.issn.1004-745X.2011.08.055.
81. Wu ZX. Analysis of Shuxuening in treating 50 cases of ischemic cerebrovascular disease. Journal of Practical Traditional Chinese Internal Medicine. 2004;18(04):370. doi:10.3969/j.issn.1671-7813.2004.04.093.
82. Xiao DF. Effect of Shuxuening on the Recovery of Nerve Function in Patients with Acute Cerebral Infarction. Guide of China Medicine. 2022;20(17):123-126. doi:10.15912/j.cnki.gocm.2022.17.041.
83. Xie RP. Clinical observation of Shuxuening injection in the treatment of acute cerebral infarction. Proceeding of Clinical Medicine. 2010;19(10):758-759. doi:10.3969/j.issn.1671-8631.2010.10.020.
84. Xie YQ, Yang XS. Clinical evaluation of Shuxuening injection in the treatment of acute cerebral infarction. Journal of Clinical Research. 2004;21(02):185-187. doi:10.3969/j.issn.1671-7171.2004.02.035.
85. Xin FB, Deng XJ. Observation of curative effect of Shuxuening injection on ischemic cerebrovascular disease. Hubei Journal of Traditional Chinese Medicine. 2005;27(10):25-26. doi:10.3969/j.issn.1000-0704.2005.10.017.
86. Xiong SC. Observation of curative effect of Shuxuening Injection on cerebral infarction. China Health Industry. 2011;8(29):49. doi:10.16659/j.cnki.1672-5654.2011.29.015.
87. Xu CY. Observation and nursing of Shuxuening in the treatment of acute cerebral infarction. Chinese and Foreign Medical Research. 2012;10(02):134-135. doi:10.3969/j.issn.1674-6805. 2012.02.111.
88. Xu L. Clinical observation of 30 cases of acute cerebral infarction treated with Shuxuening needle. Guide of China Medicine. 2008;6(23):333-334. doi:10.3969/j.issn.1671-8194.2008.23.250.
89. Xu PF, Cai SE. Shuxuening injection treating 35 cases of acute cerebral infarction. Journal of Practical Traditional Chinese Medicine. 2011;27(04):258. doi:10.3969/j.issn.1004-2814.2011.04.037.
90. Xue JY. Observation on therapeutic effect of Shuxuening Injection on ischemic cerebrovascular disease. China & Foreign Medical Treatment. 2013;32(15):106-108. doi:10.3969/j.issn.1674-0742.2013.15.063.
91. Yan TQ. Effect of Shuxuening Injection on cerebral infarction and its influence on hemorheology. Guide of China Medicine. 2016;14(28):200. doi:10.15912/j.cnki.gocm.2016.28.165.
92. Yan XY, Tan SW. Effect of Shuxuening injection on hemorheology in patients with acute cerebral infarction. Clinical Journal of Traditional Chinese Medicine. 2010;22(11):987-988.
93. Yang JH. Clinical observation of Shuxuening injection in treating 48 cases of acute cerebral infarction. Guiding Journal of Traditional Chinese Medicine and Pharmacy. 2010;16(01):38-39. doi:10.13862/j.cnki.cn43-1446/r.2010.01.010.
94. Yang M.Curative effect of Shuxuening Injection on acute cerebral infarction. Journal of Medical Forum. 2009;30(14):78-79. doi:CNKI:SUN:HYYX.0.2009-14-044.
95. Yang XW, Gao M, Chen JL, He ML. Effect of Shuxuening Injection on ACTH, ET, CORT, TXB2 and PGI2 in acute cerebral infarction. Journal of Emergency in Traditional Chinese Medicine. 2006;15(06):568-569. doi:10.3969/j.issn.1004-745X.2006.06.002.
96. Yin HX, Wang ZY, Lin S, Luo ZP, Li W, Chen SR. Clinical study on Acute cerebral infarction treated by 999-Shuxuening Injection. Journal of Clinical Research. 2004;21(07):775-777. doi:10.3969/j.issn.1671-7171.2004.07.023.
97. Yin ZL. Analysis and evaluation of Shuxuening injection in the treatment of acute cerebral Infarction. Electronic Journal of Clinical Medical Literature. 2020;7(42):163. doi:10.16281/j.cnki.jocml.2020.42.143.
98. Yu BQ, Rui XD, Huang MZ. Clinical effect of Shuxuening injection on cerebral infarction. Journal of Nanjing Military Medical College. 2003;25(4):282-283. doi:CNKI:SUN:NJJB.0.2003-04-033.
99. Zang ZX, Liu ZQ. Effectof Shuxuening Injectionon Serum Lipid and Fibrinogen of the Acute Cerebral Infarction. 2010;19(8):1290-1292. doi:10.3969/j.issn.1004-745X.2010.08.013.
100. Zhang GJ. Curative effect of Shuxuening in the treatment of acute cerebral infarction. Chinese Journal of Modern Drug Application. 2010;4(07):132-133. doi:10.3969/j.issn.1673-9523.2010.07.122.
101. Zhang H, Zhou M, Zhang JJ. The therapeutic effect of Shuxuening injection on acute cerebral infarction and its mechanism. China Journal of Traditional Chinese Medicine and Pharmacy. 2009;24(01):81-84. doi:10.3969/j.issn.1004-7484(s).2013.09.542.
102. Zhang HM. Clinical observation of Shuxuening Injection in the treatment of 80 cases of ischemic stroke. Chinese General Practice. 2004;7(15):1092. doi:10.3969/j.issn.1007-9572.2004.15.045.
103. Zhang HX, Gu JQ, Zhao SQ, Wang HC, Hu L. The effect of Shuxuening Injection on plasma concentrations of lysophosphatidic acid and phosphatidic acid in patients with cerebral infarction. International Journal of Cerebrovascular Diseases. 2006;14(03):197-200. doi:10.3760/cma.j.issn.1673-4165.2006.03.004.
104. Zhang L, Shi MQ. Treating of 80 cases of acute cerebral infarction with Shuxuening injection. Clinical Journal of Chinese Medicine. 2010;2(10):57,61. doi:10.3969/j.issn.1674-7860.2010.10.036.
105. Zhang L. Clinical observation of Shuxuening in the treatment of acute cerebral infarction. Jilin Medical Journal. 2007;28(17):1879-1880. doi:10.3969/j.issn.1004-0412.2007.17.035.
106. Zhang MX. Clinical effect of Shuxuening injection in the treatment of acute cerebral infarction. Renowned Doctor. 2018;(03):18.
107. Zhang RL, Yan XL. Clinical effect of Shuxuening injection on 67 cases of acute cerebral infarction. Hainan Medical Journal. 2008;19(02):70-71. doi:10.3969/j.issn.1003-6350.2008.02.041.
108. Zhang XK. Effect of Shuxuening on therapeutic effect, degree of neurological impairment and recovery prognosis of patients with cerebral infarction. Systems Medicine. 2017;2(08):51-53. doi:10.19368/j.cnki.2096-1782.2017.08.051.
109. Zhang XZ. Effect of ShuXueNing on the Treatment Curative Effect, Nerve Function Defect Degree and Recovery Prognosis of Patients with Cerebral Infarction. Journal of North Pharmacy. 2018;15(02):93-94. doi:10.3969/j.issn.1672-8351.2018.02.080.
110. Zhang YP. Clinical effect of Shuxuening injection on acute cerebral infarction. Proceeding of Clinical Medicine. 2004;13(8):586-587. doi:10.3969/j.issn.1671-8631.2004.08.014.
111. Zhao YX. Clinical observation of Shuxuening in the treatment of cerebral infarction. Journal of Frontiers of Medicine. 2015;5(36):87-88. doi:10.3969/j.issn.2095-1752.2015.36.086.
112. Zheng YZ. Curative effect of Shuxuening injection in the treatment of cerebral infarction. Guide of China Medicine. 2014;12(1):183-184. doi:10.15912/j.cnki.gocm.2014.01.178.
113. Zhu L. Clinical efficacy and safety analysis of Shuxuening in the treatment of acute cerebral infarction. Chinese Journal of Control of Endemic Diseases. 2014;29(S2):201. doi:CNKI:SUN:DYBF.0.2014-S2-227
114. Zhu XJ. Effect of Shuxuening on hemorheology of acute cerebral infarction. China Health Care & Nutrition. 2013;2013(5):188-189.
115. Zhuang JS. Comparison of clinical efficacy of Shuxuening Injection in the treatment of 77 cases of acute cerebral infarction. The China Journal of Modern Medicine and Science and Technology. 2004;4(2):11.
116. Zi XH, Xu HQ, Guo K, Fan XJ, Yu H. Clinical observation of Shuxuening injection in the treatment of acute ischemic stroke. Chinese Journal of Geriatrics. 2004;23(09):67-68. doi:10.3760/j:issn:0254-9026.2004.09.022.

**Excluded studies (n=95):**

**Repeated article (n=1)**

1. Li HP, Chen JH, Yang QM. Study on the efficacy of Shuxuening injection in the treatment of cerebral infarction in the elderly. Chinese Journal of Arteriosclerosis. 2006;14(3):240-242. doi:10.3969/j.issn.1007-3949.2006.03.016.

**Retrospective study (n=1)**

1. Guo XL. Observation on the efficacy of Shuxuening injection in the treatment of acute cerebral infarction. Chinese Journal of Practical Nervous Diseases. 2013;16(16):61-62. doi:10.3969/j.issn.1673-5110.2013.16.039.

**Not meet intervention (n=5)**

1. Chen JB. Effects of Shuxuening injection on neurological function and haemorheology in patients with ischaemic stroke. Chinese Journal of Neuroimmunology and Neurology. 2010;17(06):453-454. doi:10.3969/j.issn.1006-2963.2010.06.021.
2. Chen JH, Zhang Y, Yi J, Xu X, Fu W. Clinical efficacy observation of Shuxuening injection in the treatment of acute cerebral infarction. Chinese Journal of Integrative Medicine on Cardio-Cerebrovascular Disease. 2008;6(09):1046-1047. doi :10.3969/j.issn.1672-1349.2008.09.021.
3. Huang ZA, Pan ZD. Influence and curative effect of Shuxuening on nerve recovery of ACR patients. Chinese Youjiang Medical Journal. 2012;40(2):171-173. doi:10.3969/j.issn.1003-1383.2012.02.010.
4. Wang RF. Observation on the effect of Shuxuening in treating acute cerebral infarction. Chinese Journal of Clinical Rational Drug Use. 2012;5(11):79-80. doi:10.15887/j.cnki.13-1389/r.2012.11.129.
5. Wei C. Observation on the effect of Shuxuening in the treatment of acute-phase cerebral thrombosis. Journal of Youjiang Medical University for Nationalities. 2004;26(1):40-41. doi:10.3969/j.issn.1001-5817.2004.01.019.

**Not meet outcomes (n=40)**

1. Cui XJ. Comparison of the efficacy of domestic and imported extract of Ginkgo in treating 60 cases of ischaemic stroke. Lishizhen Medicine and Materia Medica Research. 2006;17(05):810-811. doi:10.3969/j.issn.1008-0805.2006.05.090.
2. Duan WQ. Curative effect comparison of Shuxuetong injection and Shuxuening injection in the treatment of acute cerebral ischemic stroke. Clinical Research and Practice. 2017;2(14):119-120. doi:10.19347/j.cnki.2096-1413.201714061.
3. Fan Y, Zhao Y, Xu J. Observation on the therapeutic effect of applying Shuxuening injection in 38 elderly patients with cerebral infarction. Journal of Frontiers of Medicine. 2015;5(10):190-190. doi:10.3969/j.issn.2095-1752.2015.10.155.
4. Feng MH, Liu RX. Observation on the therapeutic effect of 60 cases of cerebral infarction treated with Shuxuening. Jilin Medical Journal. 2010;31(7):964. doi:10.3969/j.issn.1004-0412.2010.07.059.
5. Fu Y, Deng ZH. The effect of shuxuening on blood rheology and coagulation-fibrinolytic system in elderly ischaemic stroke. Chinese Journal of Gerontology. 2012;32(23):5253-5254. doi:10.3969/j.issn.1005-9202.2012.23.073.
6. Gao M, Yang XW, Chen DQ, Liu XJ, Chen JL, Huang NB. Effect of Shuxuening injection on serum soluble cell adhesion molecules in acute cerebral infarction. Journal of Emergency in Traditional Chinese Medicine. 2005;14(01):14-15. doi:10.3969/j.issn.1004-745X.2005.01.007.
7. Gao Y, Shi YQ, Hu YL. Study on effect and safety of treatment of acute ischemic stroke compared Shuxuening with Shuxuetong by in-travenous infusion. Chinese Journal of Practical Nervous Diseases. 2015;18(06):20-22. doi:10.3969/j.issn. 1673-5110.2015.06.011.
8. Gu P. Analysis of the clinical efficacy of Shuxuening Injection in the treatment of 60 cases of cerebral infarction. Chinese Journal of Modern Drug Application. 2009;3(21):88-89. doi:10.3969/j.issn.1673-9523.2009.21.077.
9. He XD. Application of 999 Shuxuening injection in the treatment of cerebral infarction. China Medical Herald. 2005;2(14):71,91. doi:10.3969/j.issn.1673-7210.2005.14.067.
10. Hou Y, Fan YL. Analysis of the efficacy of Shuxuening injection in the treatment of acute cerebral infarction. China Practical Medicine. 2008;3(14):102-103. doi:10.3969/j.issn.1673-7555.2008.14.074.
11. Huang NB, Long YJ, Yang XW. Clinical observation of Shuxuening for acute cerebral infarction. Journal of Practical Traditional Chinese Medicine. 2005;21(7):424-425. doi:10.3969/j.issn.1004-2814.2005.07.042.
12. Huang PH. Observation on the efficacy of Shuxuening injection in the treatment of elderly ischemic stroke. Practical Journal of Cardiac Cerebral Pneumal and Vascular Disease. 2011;19(4):589-590. doi:10.3969/j.issn.1008-5971.2011.04.040.
13. Huang YJ, Shi L, Liu YQ. Effect of shuxuening injection on plasma lysophosphatidic acid and phosphatidic acid content in patients with cerebral infarction. Hebei Medical Journal. 2011;33(2):216-218. doi:10.3969/j.issn.1002-7386.2011.02.029.
14. Lan XQ, Jin L, Zhang DZ, Zhang M, Yang Z, Chen B, Chen W, Xing ZG. Effects of Shuxuening Injection on the Treatment of Hematencephalon. Practical Journal of Cardiac Cerebral Pneumal and Vascular Disease. 2010;18(1):23-24. doi:10.3969/j.issn.1008-5971.2010.01.011.
15. Li HF, Liu CQ. Treatment of acute cerebral infarction with Shuxuening injection in 68 cases. Chinese Journal of Traditional Medical Science and Technology. 2010;17(5):460-461. doi:10.3969/j.issn.1005-7072.2010.05.070.
16. Li HP, Chen JH, Yang QM. Study on the efficacy of Shuxuening injection in the treatment of cerebral infarction in the elderly. Chinese Journal of Arteriosclerosis. 2006;14(3):240-242. doi:10.3969/j.issn.1007-3949.2006.03.016.
17. Li L, Sun LY. Clinical study of Shuxuening injection in the treatment of patients with acute cerebral infarction. Health Horizon. 2013;21(16):152-152.
18. Li L. Influence of Shuxuening on clinical efficacy, neurological functional recovery and hemorheology in acute cerebral infarction patients. Modern Preventive Medicine. 2012;39(9):2341-2343. doi:CNKI:SUN:XDYF.0.2012-09-094.
19. Li ZW. Effect of Shuxuening Injection on CRP and TXB 2 in plasma of acute cerebral infarction patients. Medicine World. 2007;(S1):68-70. doi:CNKI:SUN:YYSO.0.2007-S1-050.
20. Li ZY. Therapeutic efficacy of Shuxuening in the treatment of patients with cerebral infarction accompanied by hyperlipidemia. Chinese Journal of Practical Nervous Diseases. 2007;10(6):57-58. doi:10.3969/j.issn.1673-5110.2007.06.042.
21. Liu JS. Analysis of the clinical outcome of 60 cases of haemorrhagic cerebral infarction. Modern Diagnosis and Treatment. 2012;23(5):468. doi:10.3969/j.issn.1001-8174.2012.05.055.
22. Liu Y, Xu QY, Nie WX. Clinical analysis of Shuxuening injection in the treatment of cerebral infarction. Journal of Chinese Modern Medicine. 2006;3(1).
23. Ma XZ, Yang QK. Effects of Shuxuening injection in the treatment of cerebral infarction. Journal of Modern Medicine & Health. 2005;21(24):3410-3411. doi:10.3969/j.issn.1009-5519.2005.24.044.
24. Pan YW, Gao HJ. A comparative clinical study of Shuxuening injection in the treatment of cerebral infarction. Chinese Primary Health Care. 2005;19(11):87-88. doi:10.3969/j.issn.1001-568X.2005.11.049.
25. Sun XX. Shuxuening injection in patients with acute ischemic stroke. Journal of Medical Aesthetics and Cosmetology. 2015;24(5):30.
26. Wang GH, Wang HL. Observation on the efficacy of Shuxuening injection in the treatment of cerebral infarction. Chinese Community Doctors. 2011,13(24):170. doi:10.3969/j.issn.1007-614x.2011.24.167.
27. Wang HR, Li L. Effects of Shuxuening injection on coagulation function and blood rheology in patients with acute cerebral infarction. New Chinese Medicine. 2014;46(08):40-42. doi:10.13457/j.cnki.jncm.2014.08.020.
28. Wang JM, Jiang HR. Clinical study on acute cerebral infarction treated by shuxuening injection: a report of 56 cases. Anhui Medical and Pharmaceutical Journal. 2008;12(10):959-960. doi:10.3969/j.issn.1009-6469.2008.10.042.
29. Wang X. Observation on the effect of Shuxuening injection in the treatment of acute cerebral infarction. Journal of Medical Information. 2009;1(6):56.
30. Wang YJ. Exploration of the clinical value of cerebral infarction treated with Shuxuening injection. Adolescent Health. 2014;2014(22):36-37. doi:10.3969/j.issn.1672-6502.2014.22.021.
31. Wang YY. Observation on 36 cases of acute cerebral infarction treated with combination of Chinese and western medicines. Chinese Journal of Clinical Practical Medicine. 2007;8(4):78-79.
32. Wu AS. Observation on the effect of Shuxuening injection in the treatment of acute cerebral infarction. Journal of Hebei Medical University. 2007;28(5):367-368. doi:10.3969/j.issn.1007-3205.2007.05.016.
33. Wu CL. Effect of early application of Shuxuening injection on the status of neurological deficits in patients with acute cerebral infarction. Journal of Emergency in Traditional Chinese Medicine. 2013;22(03):468-469. doi:10.3969/j.issn.1004-745X.2013.03.
34. Xiao YP. Clinical analysis of Shuxuening for treating 96 cases of ischemic cerebrovascular disease. Journal of Modern Medicine & Health. 2012;28(09):1407-1408. doi:CNKI:SUN:XYWS.0.2012-09-091.
35. Xiong XD. Effects of Shuxuening injection on neurological function and blood rheology in patients with ischemic stroke. Chinese and Foreign Medical Research. 2012;10(07):25-26. doi:10.14033/j.cnki.cfmr.2012.07.012.
36. Yang YH. Observation on clinical effect of Shuxuening injection in the treatment of acute cerebral infarction. Shanxi Journal of Traditional Chinese Medicine. 2006;22(5):16-17. doi:10.3969/j.issn.1000-7156.2006.05.007.
37. Yu GP, Zhu HY, Yu JM. Clinical analysis of 58 cases of atherosclerotic cerebral infarction treated with Shuxuening injection. Cardio-cerebrovascular Disease Prevention and Treatment. 2006;6(5):334-335. doi:10.3969/j.issn.1009-816X.2006.05.030.
38. Zhang DM, Zhang GQ. Observation on clinical effect of Shuxuening injection in the treatment of acute cerebral infarction. Journal of Qiqihar Medical University. 2004;25(01):23. doi:10.3969/j.issn.1002-1256.2004.01.015.
39. Zhao XJ, Xu, LH. Observation on clinical effect of Shuxuening injection in the treatment of acute cerebral infarction. Contemporary Medicine. 2013;19(34):147+164. doi:10.3969/j.issn.1009-4393.2013.34.114.
40. Zhou YM. Shuxuening injection on the levels of inflammatory cytokines with acute cerebral hemorrhage. MEDICAL INFORMATION. 2011;24(07):2928-2929. doi:10.3969/j.issn.1006-1959.2011.07.061.

**Not meet the diagnostic criteria of AIS (n=15)**

1. Dong YX. The effect of Shuxuening injection on the rate of haematoma absorption in hypertensive cerebral haemorrhage. Journal of Clinical Research. 2012;29(11):2212-2213.
2. Li L. Clinical efficacy observation of Shuxuening in the treatment of haemorrhagic cerebral infarction. Contemporary Medicine. 2015;21(31):125-126. doi:10.3969/j.issn.1009-4393.2015.31.082.
3. Liu Q. Observations on the efficacy of Shuxuening injection in the treatment of hypertensive cerebral haemorrhage during the recovery period. Chinese Journal of Practical Nervous Diseases. 2007;10(5):101-102. doi:10.3969/j.issn.1673-5110.2007.05.075.
4. Liu T, Liu YH, He QC. Analysis of the therapeutic effect of 35 cases of hypertensive cerebral haemorrhage treated with the addition of Shuxuening Injection. Lishizhen Medicine and Materia Medica Research. 2010;21(3):689-690. doi:10.3969/j.issn.1008-0805.2010.03.087.
5. Ma SP, Zhang X. Clinical study on the treatment of hypertensive cerebral hemorrhage with Shuxuening injection. Chinese Community Doctors. 2005;(23):17.
6. Wang F. Efficacy of Shuxuening in the treatment of non-traumatic cerebral hemorrhage and its effect on blood rheology. Journal of China Prescription Drug. 2015;13(6):73-74. doi:10.3969/j.issn.1671-945X.2015.06.052.
7. Wang LX, Wang CJ. Observation on the efficacy of Shuxuening injection in the treatment of hypertensive cerebral hemorrhage. Guide of China Medicine. 2010;8(25):124-125. doi:10.15912/j.cnki.gocm.2010.25.097.
8. Wu GP. Clinical study of Shuxuening injection in the treatment of primary cerebral hemorrhage. Zhejiang Journal of Traditional Chinese Medicine. 2007;42(4):200-201. doi:10.3969/j.issn.0411-8421.2007.04.007.
9. Wu XL. Effects of shuxuening in the treatment of hypertensive cerebral hemorrhage. Guide of China Medicine. 2012;10(25):285-286. doi:10.15912/j.cnki.gocm.2012.25.247.
10. Yu CH. Clinical observation on 40 cases of patients with hypertensive cerebral haemorrhage treated with Shuxuening Injection. The Medical Forum. 2010;14(S1):25-26. doi:10.3969/j.issn.1672-1721.2010.z1.018.
11. Zhou J, Guo RL. Effects of Shuxuening injection on the treatment of Hematencephalon. Asia-PacificTraditional Medicine. 2009;5(11):61-63. doi:CNKI:SUN:YTCT.0.2009-11-032.
12. Bai ZH, Yan YM. Analysis of the efficacy of 48 cases of cerebral infarction in the acute stage treated by Shuxuening. Modern Chinese Medicine. 2006;26(03):17-18. doi:10.3969/j.issn.1672-0571.2006.03.010.
13. Gao YJ, Gao XR, Liu F. Clinical Effect of Shuxuening Injection in the Adjuvant Treatment of Cerebral Infarction. Practical Journal of Cardiac Cerebral Pneumal and Vascular Disease. 2017;25(01):125-126. doi:10.3969/j.issn.1008-5971. 2017.01.035.
14. Guo JY, Liu ML. Observation on the efficacy of Shuxuening Injection in the treatment of acute cerebral infarction. Modern Journal of Integrated Traditional Chinese and Western Medicine. 2009;18(24):2926-2927. doi:10.3969/j.issn.1008-8849.2009.24.027.
15. Zhang L, Ma BH. Clinical observation on 30 cases of cerebral infarction treated with Shuxuening injection. Hebei Medicine. 2006;12(07):681-682. doi:CNKI:SUN:HCYX.0.2006-07-055.

**Not meet the course of stroke (n=21)**

1. Gao YL. Efficacy of Shuxuening in the treatment of acute cerebral infarction. Electronic Journal of Clinical Medical Literature. 2015;2(09):1630. doi:10.16281/j.cnki.jocml.2015.09.035.
2. Ge XX. Efficacy of shuxuening in the treatment of acute cerebral infarction and its effect on the recovery of neurological functions. China Practical Medicine. 2014;9(04):135-136. doi:10.14163/j.cnki.11-5547/r.2014.04.183.
3. Guo JY, Liu ML. Observation on the efficacy of Shuxuening injection in the treatment of acute cerebral infarction. Modern Journal of Integrated Traditional Chinese and Western Medicine. 2009;18(24):2926-2927. doi:10.3969/j.issn.1008-8849.2009.24.027.
4. Hao JH. Observation on the efficacy of Shuxuening injection in the treatment of acute cerebral infarction. Chinese Journal of Practical Nervous Diseases. 2011;14(1):42-43. doi:10.3969/j.issn.1673-5110.2011.01.022.
5. Hou YF, Ding J, He JG, Xu ZQ, Zhao CX, Men M. Clinical evaluation on treatment of acute cerebral infarction with Shuxuening injection. Journal of Clinical Research. 2005;22(1):44-46. doi:10.3969/j.issn.1671-7171.2005.01.015.
6. Li QP, Liu YJ. Clinical efficacy observation of Shuxuening injection in the treatment of acute cerebral infarction. Chinese Journal of Modern Drug Application. 2009;3(14):134-135. doi:10.3969/j.issn.1673-9523.2009.14.103.
7. Li YR, Li HS. Clinical efficacy of Shuxuening injection in the treatment of ischaemic stroke during the recovery period. Tianjin Pharmacy. 2015;27(5):44-45. doi:10.3969/j.issn.1006-5687.2015.05.017.
8. Ma SY, Shen PH. Observation on the efficacy of Shuxuening injection in treating 55 cases of acute cerebral infarction. China Medical Herald. 2008;5(33):52-53. doi:10.3969/j.issn.1673-7210.2008.33.035.
9. Ning ZX. Effects of Shuxuening injection in the treatment of acute cerebral infarction. Psychological Doctor. 2019;25(4):64-65.
10. Qiao YC. Observation on effect of Shuxuening injection in treating 126 cases of cerebral infarction. Heilongjiang Medicine and Pharmacy. 2007;30(01):120. doi:10.3969/j.issn.1008-0104.2007.01.072.
11. Qiu JM. Observation on the effect of Shuxuening in the treatment of cerebral infarction. Chinese Medicine of Factory and Mine. 2008;21(2):224. doi:10.3969/j.issn.1674-8182.2008.02.072.
12. Shao WX. The difference between therapeutic effect of danshen chuanxiongqin injection and Shuxuening injection on acute cerebral infarction. Chinese Medicine Modern Distance Education of China. 2014;12(11):15-16. doi:10.3969/j.issn.1672-2779.2014.11.008.
13. Wang JM. Comparative study on the effectiveness and safety of Shuxuening injection versus Shuxuetong injection for the treatment of acute ischemic stroke. Psychological Doctor. 2016;22(17):45-46.
14. Wang JQ. Observation on effect of Shuxuening injection in the treatment of acute cerebral infarction. Chinese Journal of Practical Medicine. 2014;41(23):121-122. doi:10.3760/cma.j.issn.1674-4756.2014.23.063.
15. Yu K, Wu ZS, Gong SJ. Clinical observation on 70 cases of acute cerebral infarction treated with 999 Shuxuening injection. China Medical Herald. 2005;2(14):70. doi:CNKI:SUN:YYCY.0.2005-14-067.
16. Zeng HH, Wang Y. Clinical observation on the effect of Shuxuening injection on acute infarction patients. China Tropical Medicine. 2005;5(05):1063-1074. doi:10.3969/j.issn.1009-9727.2005.05.069.
17. Zhang AH. Observation on the efficacy of Shuxuening in the treatment of acute cerebral infarction. Journal of Chinese Practical Diagnosis and Therapy. 2007;21(09):714-715. doi:10.3969/j.issn.1674-3474.2007.09.047.
18. Zhang CL, Sun XP. Clinical observation on 34 cases of acute cerebral infarction treated with Shuxuening. China Practical Medicine. 2010;5(30):130-131. doi:10.3969/j.issn.1673-7555.2010.30.104.
19. Zhang Y, Luo BY. Effect of Ginkgo biloba extract on the platelet membrane glycoprotein in patients with cerebral infarction. Zhejiang Medical Journal. 2005;27(02):99-100+135. doi:10.3969/j.issn.1006-2785.2005.02.008.
20. Zhang YP, Xiao CN, Xie JC. Efficacy of Shuxuening injection in the treatment of acute cerebral infarction and its effect on blood rheology. Jiangxi Medical Journal. 2007;42(10):896-897. doi:10.3969/j.issn.1006-2238.2007.10.020.
21. Zhou FQ. Effects of Shuxuening in the treatment of cerebral infarction. Chinese Journal of Modern Drug Application. 2011;5(09):54-55. doi:10.3969/j.issn.1673-9523.2011.09.048.

**Unclear about the dose of SXNI (n=3)**

1. Li CP. Clinic observation of Shuxuening injection on patients with acute cerebral infarction. Journal of Shanxi University of Chinese Medicine. 2007;8(1):37-38. doi:10.3969/j.issn.1671-0258.2007.01.020.
2. Li N, Chen JH, Zhao JR, Yang R, Gao JS, Wang TJ. Effect of Shuxuening injection on hemorheological and biochemical indexes in patients with ischemic stroke. China Pharmaceuticals. 2016;25(04):62-64.doi:CNKI:SUN:YYGZ.0.2016-04-024.
3. Ma X, Qu XX. Effect of Shuxuening Injection on Plasma Endothelin and Hemorheologic Indexes in Patients with Acute Cerebral Infarction. China Pharmaceuticals. 2014;23(16):39-41. doi:CNKI:SUN:YYGZ.0.2014-16-021.

**Unclear about the intervention duration of SXNI (n=6)**

1. Li M, Li KW, Cui LX, He JQ, Xiao YL. Analysis of the clinical effect of Shuxuening injection in the treatment of cerebral infarction. Journal of North Pharmacy. 2020;17(2):36-37. doi:10.3969/j.issn.1672-8351.2020.02.024.
2. Lin LY, Li L, Tang L, Zhang Y. Effect of Shuxuening for 60 cases of acute cerebral infarction with syndrome of static blood blocking collaterals. Western Journal of Traditional Chinese Medicine. 2011;24(5):57. doi:10.3969/j.issn.1004-6852.2011.05.027.
3. Sun JS, Qiao CY, Dong K. Clinical observation on the treatment of acute cerebral infarction with Shuxuening injection. China Medical Herald. 2007;4(22):107-108. doi:10.3969/j.issn.1673-7210.2007.22.077.
4. Wang HR, Yang XP. Effects of shuxuening on LPA in patients with acute cerebral infarction. Chinese Journal of Practical Nervous Diseases. 2006;9(3):83-84. doi:10.3969/j.issn.1673-5110.2006.03.054.
5. Xu MC, Xu JJ, Shao YM. Effect of Shuxuening injection on neurological function defect and cognitive function after acute cerebral infarction. New Chinese Medicine. 2021;53(05):63-65. doi:10.13457/j.cnki.jncm.2021.05.016.
6. Xue Q, Chen XC. Clinical observation of Shuxuening cooperating in the treatment of acute cerebral infarction. Acta Neuropharmacologica. 2004,21(2):11-13. DOI:10.3969/j.issn.2095-1396.2004.02.006.

**Data error (n=3)**

1. Hong JH, Luo HT, Xue CH. Observation on clinical effect of Shuxuening in the treatment of cerebral infarction. Chinese Community Doctors. 2008;10(20):28. doi:CNKI:SUN:ZGSQ.0.2008-20-031.
2. Wan SQ, Wang SJ, Ding BH. Efficacy of the Shuxuening injection on acute cerebral infarction and plasma endothelin, hemorheologic indexes. Clinical Journal of Chinese Medicine. 2018;10(13):13-15. doi:CNKI:SUN:ZYLY.0.2018-13-007.
3. Wang GZ. Observation on the efficacy of Shuxuening injection in treating 46 cases of acute cerebral infarction. The Medical Forum. 2017;21(32):4588-4589. doi:10.19435/j.1672-1721.2017.32.117.
